# The post-cranial anatomy and functional morphology of *Conoryctes comma* (Mammalia: Taeniodonta) from the Paleocene of North America

**DOI:** 10.1371/journal.pone.0311053

**Published:** 2024-10-25

**Authors:** Zoi Kynigopoulou, Sarah L. Shelley, Thomas E. Williamson, Stephen L. Brusatte

**Affiliations:** 1 School of GeoSciences, University of Edinburgh, Edinburgh, Scotland, United Kingdom; 2 New Mexico Museum of Natural History and Science, Albuquerque, New Mexico, United States of America; Brunel University London, UNITED KINGDOM OF GREAT BRITAIN AND NORTHERN IRELAND

## Abstract

*Conoryctes comma* is a member of the enigmatic group Taeniodonta, Paleogene mammals that have been found only in North America. Taeniodonts were part of the first wave of placental mammal diversification after the end-Cretaceous extinction. The lack of postcranial elements has limited the understanding of the anatomy and locomotion of *Conoryctes*, and how it compared to other taeniodonts. We here describe the postcranial anatomy and functional morphology of *Conoryctes*, based largely on nine new specimens found in the San Juan Basin, New Mexico, USA. The specimens include elements of the axial column, such as the axis, sacrum, and ribs; the humerus, ulna, radius, and part of the manus; the innominate, femur, tibia, and part of the pes, including the tarsals. *Conoryctes* was a medium-sized mammal, with a robust humerus, radius, and femur, and with anatomical similarities to other conoryctid taeniodonts and *Onychodectes*. The tarsal elements of *Conoryctes* show characteristics of the “leptictimorph astragalocalcaneal morphology” as seen in other Paleogene mammals, such as *Escavadodon*, *Palaeanodon*, and *Procerberus*. Anatomical features of the forelimb and hindlimb of *Conoryctes* indicate that it was a scratch-digging animal with powerful forearm muscles and well-stabilized digits, features that may have helped it adapt to the subtropical forests of the San Juan Basin, approximately 63 million years ago. This corroborates the previous hypothesis that digging adaptations are seen in all members of Taeniodonta for which the postcranial elements are known, and that digging ability was present in the common ancestor of the clade and potentially central to their radiation after the environmental destruction of the end-Cretaceous extinction.

## Introduction

The diversification of mammals after the end-Cretaceous mass extinction still raises many questions, for example on the ecological niches these mammals were occupying. Mammals that managed to survive and proliferate immediately after the extinction can provide valuable information on how organisms are affected by extreme environmental change, and how the diversity of modern-day mammals was assembled. The term “archaic” mammals is used to describe the groups of Paleocene-Eocene mammals whose relationship with modern-day mammals is uncertain, making it unclear whether they left any obvious modern descendants [1]. Taeniodonta [2] is an enigmatic group of “archaic” mammals known from Paleogene localities of North America. Their highly worn teeth (some with complete tooth rows of ever-growing teeth) and robust bodies are distinctive among other animals of their time.

There are nine currently recognized genera of taeniodonts, traditionally arranged into two families: the smaller Conoryctidae with the genera *Conoryctella*, *Huerfanodon*, and *Conoryctes*, and the more robust Stylinodontidae, which includes *Wortmania*, *Psittacotherium*, *Ectoganus*, and *Stylinodon* [3, 6]. *Onychodectes* is a basal taeniodont possibly outside of the two main families [3], while other studies find it to be basal within Stylinodontidae [4]. The lowest putative taeniodont stratigraphically is *Schowalteria* found in the Upper Cretaceous Scollard Formation of Alberta, Canada [5]. Therefore, taeniodonts are proposed to have originated before the Cretaceous-Paleogene boundary, placing them among the animals that survived the extinction.

Postcranial elements are generally rare for taeniodonts. However, the robust postcranial elements of *Psittacotherium*, *Ectoganus* and *Stylinodon* show clear adaptations to fossoriality [6, 7]. Previous studies suggest that earlier taeniodonts, like *Onychodectes*, *Conoryctes*, and *Conoryctella* had generalised postcranial skeletons [1, 6, 8, 9]. Many Paleocene mammals have been proposed as having a “generalised body plan”, but the study by Shelley *et al*. [10] showed a high diversity of tarsal morphology, indicating a broader range of locomotory habits and functional morphologies than is often assumed.

Williamson and Brusatte [3] described new postcranial elements of *Wortmania* and thoroughly explained the anatomical features that point to digging adaptations for the genus. In that study, they hypothesised that even the basal *Onychodectes* had anatomical characteristics indicative of digging behaviour. They also emphasised the need for new fossils to assess whether conoryctids were also able to dig at least to some degree. The goal of the present study is to understand the anatomy and functional morphology of *Conoryctes*, an early Paleocene conoryctid, and to address its postcranial adaptations, particularly to determine whether it had features of the bones and muscular attachments that were indicative of digging behaviour.

*Conoryctes* is known from the early Paleocene of the San Juan Basin in New Mexico, USA, mainly from dental specimens. Only a partial humerus and a radius of the specimen AMNH 3396 have previously been assigned to *Conoryctes* [6]. The lack of postcranial specimens led to unanswered questions regarding the anatomy of *Conoryctes*, and only vague understanding of its locomotion. We here describe nine new specimens of *Conoryctes* from the San Juan Basin, consisting mostly of postcranial elements, which help illuminate the hitherto enigmatic postcranial skeleton. The present study includes a detailed description of the vertebrae, forelimb and hindlimb of *Conoryctes*, as well as comparisons with other taeniodonts and Paleogene mammals. Lastly, using the new specimens, and anatomical observations of the skeleton, we evaluate the locomotor behaviour of *Conoryctes*.

### Institutional abbreviations

AMNH: American Museum of Natural History, New York City, New York, USA

FMNH, P or PM: Field Museum of Natural History, Chicago, Illinois, USA

NMMNH: New Mexico Museum of Natural History and Science, Albuquerque, New Mexico, USA

TMM: Texas Memorial Museum, University of Texas, Austin, Texas, USA

UM: Museum of Paleontology, University of Michigan, Ann Arbor, Michigan, USA

USGS: U.S. Geological Survey, Paleontology and Stratigraphy Branch, Denver, Colorado, USA

USNM: National Museum of Natural History, Washington, D. C., USA

UW: University of Wyoming, Laramie, Wyoming, USA

YPM (PU): Peabody Museum of Natural History, Yale University, New Haven, Connecticut, USA.

### Historical background

**Fig 1:**
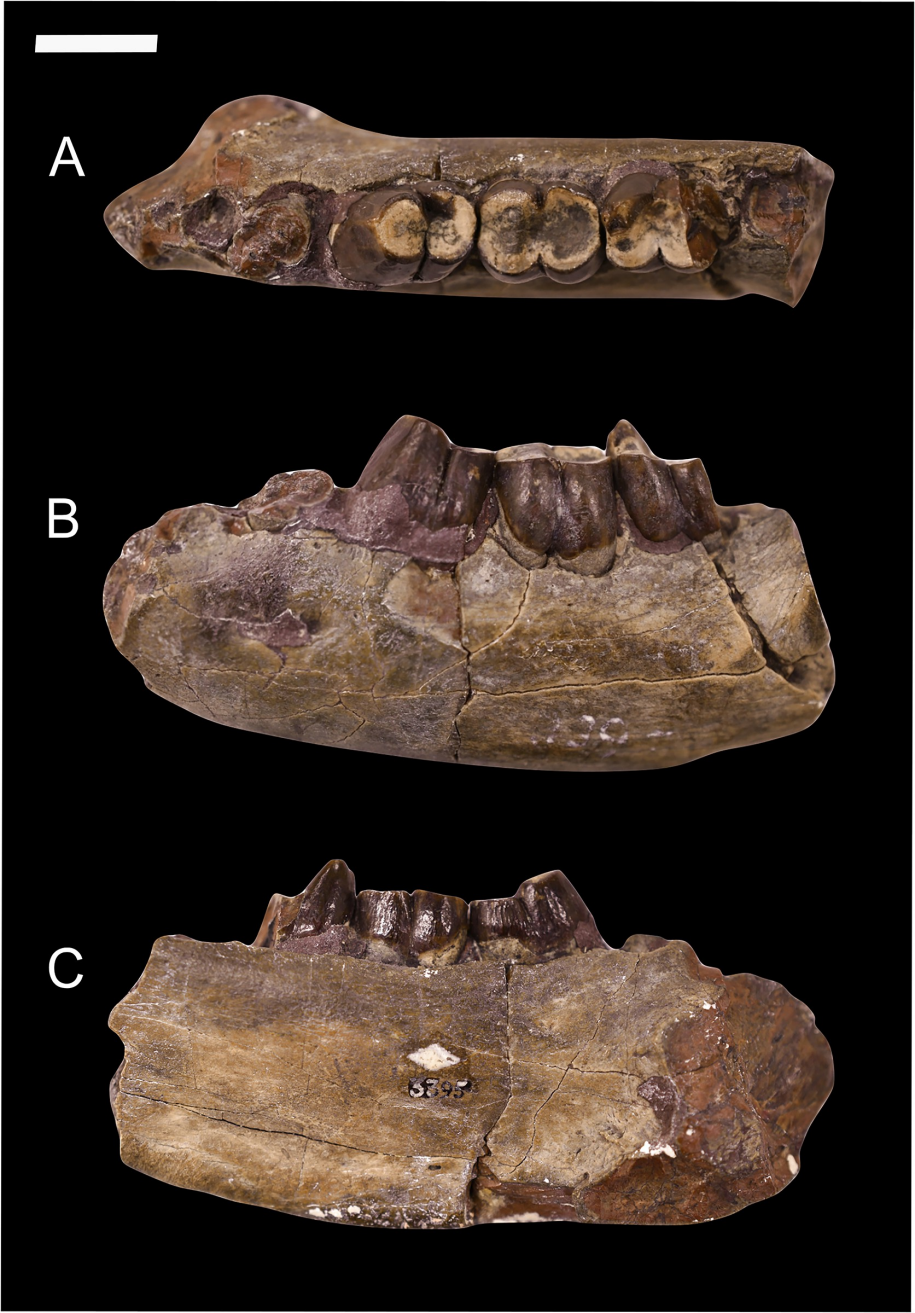
Type specimen of *Conoryctes comma* (AMNH 3395) including a partial left mandible with worn p5, m1 and m2, in occlusal (A), buccal (B) and lingual (C) views. Scale bar is 1cm.

The genus *Conoryctes* was first established and diagnosed by Cope in 1881 [11] based on a partial lower jaw (AMNH 3395) preserving a damaged and worn p5, m1 and m2 (Fig 1). In 1884, Cope [12] described the specimen AMNH 3396 as a new taxon named *Hexodon molestus*. This specimen consists of a partial skull, with a left upper canine, right P4, left and right P5––M3, an almost complete mandible with lower canines, right p4, left p5, left and right m1, left m2, right and left m3, and a partial humerus and radius, which Cope thought was part of the tibia. In the years that followed, Cope described *Onychodectes* [13] and *Hemiganus* (= *Wortmania*) [14]. In that publication [14], Cope synonymised the taxon *Hexodon molestus* with *Conoryctes comma* and assigned both specimens (AMNH 3395, AMNH 3396) to *Conoryctes*. He also said that *Conoryctes* might belong to Creodonta because it had few similarities with Condylarthra, but its dentition is more similar to that of *Onychodectes* and *Hemiganus*. Later Wortman [15] erected Ganodonta, including the then-known Taeniodonta (*Calamodon* and *Ectoganus*), *Psittacotherium* and *Hemiganus* (= *Wortmania*), *Onychodectes* and *Conoryctes*. Wortman also introduced the families Stylinodontidae and Conoryctidae, with the latter including *Onychodectes* and *Conoryctes*.

A more detailed description by Matthew [16] followed, where he considered Ganodonta to be a junior synonym of Taeniodonta. In that study, Matthew also described in detail the anatomy of the teeth, skull, mandible and the limited postcranial material of *Conoryctes* and pointed out anatomical features that showed *Conoryctes* was more specialised than *Onychodectes*. The similarities between *Conoryctes*, *Onychodectes* and *Wortmania* noticed by Cope [13] were also discussed by Matthew [16] and Patterson [8]. The latter also proposed that *Conoryctes* is more closely related to *Onychodectes* than to *Wortmania* and Stylinodontidae.

A detailed study on all taeniodonts was published by Schoch [6], who reported all known specimens that at the time could be assigned to *Conoryctes* and gave a detailed description of the dentition and postcranium while comparing *Conoryctes* to other taeniodonts. Because of similarities between *Conoryctes* and the newly established taeniodont *Huerfanodon* [17], three specimens are referred to as “Conoryctid Genus Indeterminate” (AMNH 832, AMNH 15939, USNM 9597) by Schoch [6].

The phylogenetic affinities of *Conoryctes* have been discussed in a few studies. Patterson [8] investigated the evolutionary rates of taeniodonts and illustrated the relationship between *Conoryctes*, *Conoryctella* and *Onychodectes*. Schoch [6] discussed the phylogeny and evolution of taeniodonts, but he did not perform a numerical phylogenetic analysis. Recent phylogenetic analyses [3, 4, 18] find *Conoryctes* within Conoryctidae, as a sister taxon to *Huerfanodon*.

### Geological setting

*Conoryctes comma* is known from the Torrejonian age (~64 to 61.7 million years ago) [19] deposits of the Nacimiento Formation of the San Juan Basin, New Mexico, USA. The San Juan Basin has a well-dated record of Paleocene animals [e.g., 20, 21].

The formation primarily consists of fluvial deposits of mudstones and sandstones, as well as moderately well-developed palaeosols and carbonaceous shales [19, 21]. The flora of the Nacimiento Formation indicates a subtropical climate, with warm and humid conditions, and dense vegetation [22, 23]. The fauna consists of a variety of mammals, reptiles, birds, fish, and molluscs [16, 21, 24]. The abundance of turtles and crocodiles, as well as the geology and the fossil record, indicates that the San Juan Basin had a warm climate, with humid forests in the early Paleocene [25, 26]. The stratigraphy of the San Juan Basin is well-studied and many of the localities are precisely dated based on lithostratigraphy, magnetostratigraphy, biostratigraphy and radioisotopic dating [20, 24]. However, some specimens of *Conoryctes* collected by Cope cannot be accurately placed stratigraphically, due to limited metadata associated with the specimens.

## Materials and methods

### New specimens

**Fig 2:**
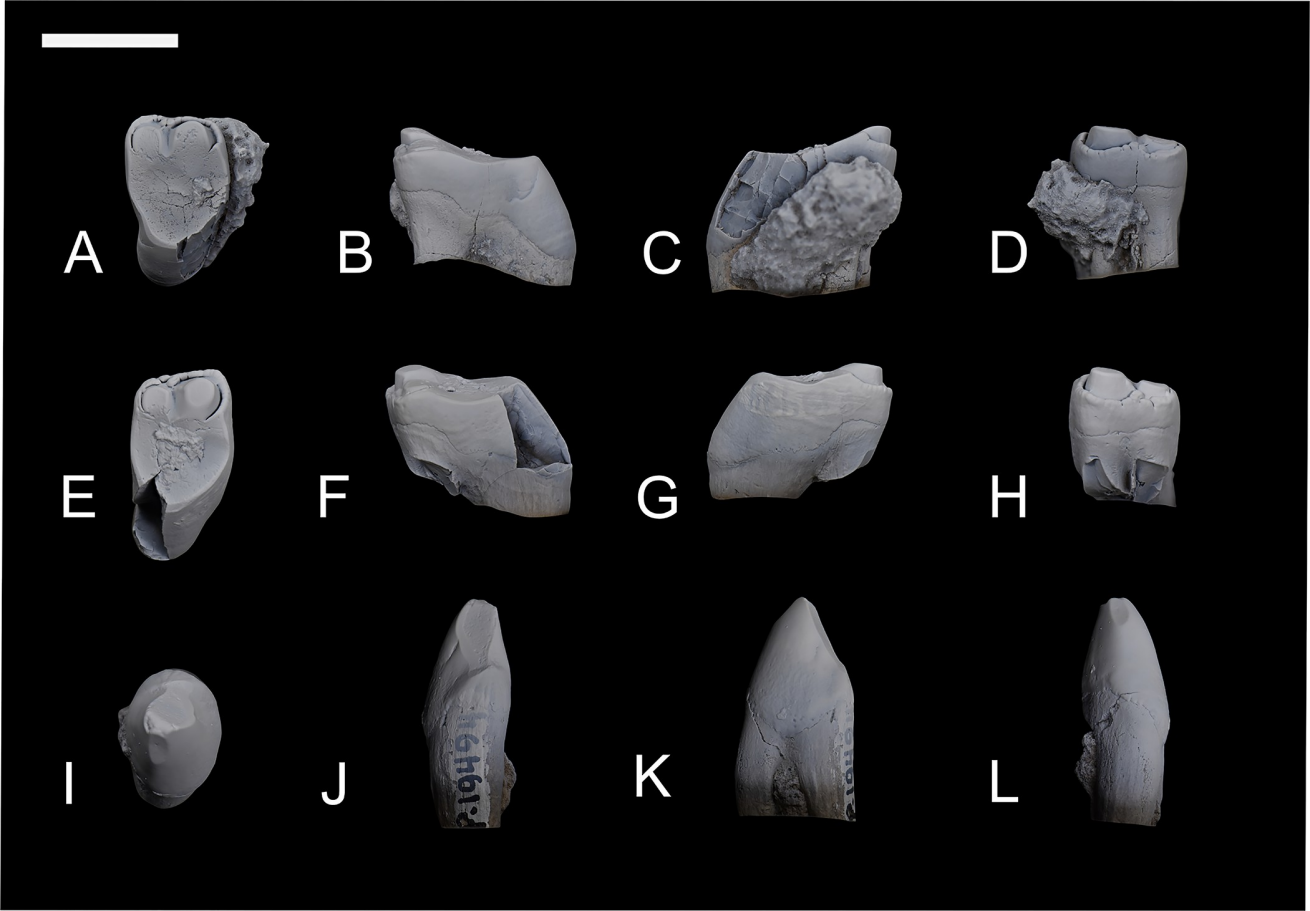
Associated teeth of *Conoryctes comma* (NMMNH P-19494). Upper M1 (A–D), upper M2 (E–H) in occlusal (A, E), distal (B, F), mesial (C, G) and buccal views (D, H). On the third row is the lower p4 (I–L) in occlusal (I), lingual (J), mesial (K) and buccal (L) views. Scale bar is 1cm.

Among the studied specimens, only NMMNH P-19494 has associated teeth (Fig 2) that can be firmly assigned to *Conoryctes comma*. After comparing the associated postcranial elements of NMMNH P-19494 to the other specimens, anatomical similarities allowed the referral of additional specimens without associated dentition (S1 Table). Therefore, NMMNH P-48198, NMMNH P-48052, NMMNH P-21509, NMMNH P-79457, NMMNH P-47700 and NMMNH P-47866 are also referred to *Conoryctes comma*. Two more specimens, NMMNH P-61789 and NMMNH P-77896, include postcranial elements but were not associated with diagnostic teeth or postcranial bones connected through a chain of association. However, they resemble the highly distinctive postcranial bones of the taeniodonts *Onychodectes* and *Psittacotherium*, but are intermediate in size and robusticity, strongly suggesting that they are referrable to a conoryctid, and most likely *Conoryctes*. They were found in strata that contain both *Conoryctes* and *Psittacotherium*, but their size most closely matches the former, because the latter is considerably larger.

Specimens that are found in the Nacimiento Formation (Torrejonian) from fossiliferous zone Tj6 [25] are: NMMNH P-19494, NMMNH P-48198, NMMNH P-48052, NMMNH P-61789 NMMNH P-77896 and NMMNH P-21509. NMMNH P-19494 consists of three associated teeth, M1, M2 and p4, and the proximal part of a femoral diaphysis, and an almost complete left tibia that is broken proximally. NMMNH P-48198 consists of eight vertebrae, an almost complete left innominate, an almost complete left tibia, a left astragalus, and a left and right calcaneum. NMMNH P-48052 includes numerous postcranial elements, most of which are fragmented. There are vertebrae, including the axis, and other postcranial elements such as a left ulna, a proximal right humerus, metacarpal and phalanges, parts of the proximal and distal femur, a complete patella, proximal and distal parts of the left tibia, and the astragalus and calcaneum. Specimens NMMNH P-61789, and NMMNH P-77896, both have a partial distal humerus and a partial innominate. NMMNH P-21509 consists of one caudal vertebra, the proximal part of a left tibia, and a left astragalus.

Specimen NMMNH P-79457 was collected from Red Mesa, Nacimiento Formation, from fossiliferous zone Tj5. It consists of two vertebrae, a sacrum, a complete right radius, two metacarpals, seven phalanges and three curved unguals, as well as the proximal part of a right femur.

Both NMMNH P-47700 and NMMNH P-47866 were found in the Angel Peak area, horizon Tj4. NMMNH P-47700 consists of numerous postcranial elements, including seven almost complete vertebrae, partial humerus, a left innominate, a partial tibia, metatarsals, phalanges and unguals. NMMNH P-47866 consists of a right calcaneum.

### Description and comparison

The skull and mandible of *Conoryctes comma* have been described in detail by Matthew [15, AMNH 15939] and Schoch [6]. Using the method of Kirk *et al*. [27] and the cranial length of these skulls, it can be determined that *Conoryctes comma* was a medium-sized animal, approximately 12–14kg, similar in weight to a beagle dog. We compared these specimens to other taeniodonts that are known from postcranial elements (i.e., *Onychodectes* [6, 15, 16], *Wortmania* [3, 6, 16], *Conoryctella* [17], *Psittacotherium* [6, 15, 16], *Ectoganus* [6, 15], and *Stylinodon* [6, 7, 15]). Moreover, we also made comparisons with other key Paleogene mammal taxa.

We used *Procerberus* as a comparative taxon because it has been proposed as closely related to Taeniodonta in many studies [3, 4, 6, 18, 28]. Based on Szalay [29], taeniodonts and other taxa like *Procerberus*, *Escavadodon*, palaeanodonts and leptictids [29–34] have a typical “leptictimorph astragalocalcaneal morphology”. These taxa exhibit features associated with extreme plantar flexion in the tibial-astragalar joint because of the increased astragalar-trochlear arch and the lack of a dorsal astragalar foramen, increased lateral stability due to a well-developed lateral border of the lateral astragalar facet on the distal tibia, expansion of the navicular facet of the astragalar head and a reduced fibular facet in the calcaneum [29].

We also compared *Conoryctes* with other archaic mammals from the San Juan Basin known from well-preserved and described postcranial skeletons: *Periptychus* and *Pantolambda* [16, 35, 36]. *Periptychus* is a well-known ‘condylarth’ placental mammal and is representative of a medium-sized terrestrial early Paleocene mammal. *Pantolambda* has been considered to be within the ‘Cimolesta’ cluster of early placental mammals (or close relatives) that may also include taeniodonts [37].

Our study protocol was as follows: measurements were made using digital callipers to the nearest two decimals, and digital measurements were taken using the software ImageJ 1.6.0, when needed [38]. Photographs of all the studied specimens were taken in the standard anatomical views using a Nikon D3500 camera and an 18 –55mm lens or a 105mm macro lens when needed. Helicon Focus 8.0.4 was used to photostack images where necessary to increase the depth of field. For the osteological and myological nomenclature, Miller’s Anatomy of the Dog [39] was mostly used, as well as the descriptive publication of *Periptychus* [35] and references therein.

## Systematic palaeontology

MAMMALIA Linnaeus 1758 [40]

EUTHERIA Gill 1872 [41]

TAENIODONTA Cope 1876 [2]

CONORYCTIDAE Wortman 1896b [42]

*Conoryctes comma* Cope, 1881 [11]

*Conoryctes comma* Cope, 1881 [43]

*Hexodon molestus* Cope, 1884 [12]

*Conoryctes comma* Cope, 1884 [44]

*Conoryctes comma* (= *Hexodon molestus*) Cope, 1888 [13]

*Conoryctes comma* Wortman, 1897 [15]

*Conoryctes comma* Matthew, 1937 [16]

*Conoryctes comma* R. W. Wilson, 1956 [45]

non *Conoryctes comma* Van Valen, 1978 [46]

non *Conoryctes comma* L. H. Taylor, 1981 [47]

Type and only known species: *Conoryctes comma* Cope 1881 [11].

### Age and locality

Middle to late Torrejonian, Danian, early Paleocene (62.8 to 61 Ma). Known from the Nacimiento Formation, San Juan Basin, New Mexico, USA.

### Etymology

Cope [11] did not provide an etymology for *Conoryctes comma*. The generic name *Conoryctes* derives from the ancient Greek words κῶνος (= cone) and ὀρύσσω (= to dig). The “cone digger” is likely referring to the conical morphology of the teeth. However, it is unclear how Cope decided upon ‘-oryctes’ given the genus was known only from dental specimens. The species name derives from the ancient Greek word κόμμα (= a part of), which is used today as the word for the punctuation mark, comma. When looking at the p5 of the type AMNH 3395 in occlusal view, it resembles a comma.

### Emended diagnosis

Medium-sized taeniodont, upper and lower canines with internal groove; lacking P1; P4 bears a minuscule to small metacone, a minuscule to well-formed lingual cingulum and an absent or small protocone; P5 is molariform with a paracone, a metacone, a small parastyle and metastyle, lacking a mesostyle and a stylar shelf; upper molars are longer buccally than lingually with a paracone, a metacone, a stylar shelf cuspidated with prominent parastyle, metastyle and mesostyle, which vary from absent to well-developed, a large protocone and no postcingulum and no hypocone; p5 bears a protoconid, lacking a paraconid and metaconid, and a cuspidate talonid; lower molars bear a mesoconid, a large hypoconid and an entoconid, one entoconulid mesial to the entoconid (= pre-entoconulid) and one distal to the entoconid (= post-entoconulid), and a cuspidated hypoconulid.

### Differential diagnosis

*Conoryctes* is larger than *Onychodectes*, *Wortmania* and *Conoryctella*, as big as *Huerfanodon*, but smaller than the other taeniodonts based on the cranial, dental and postcranial measurements. *Conoryctes* lacks an upper P1, unlike other taeniodonts apart from *Huerfanodon torrejonius*. The upper premolar P4 has a variable lingual cingulum and a protocone in *Conoryctes comma* whereas *H*. *torrejonius* has a more prominent metacone and protocone. On the upper molars, the stylar shelf is less buccally extended in *Conoryctes* than in *Onychodectes* and *Conoryctella*. In *Conoryctes* the enamel of the upper teeth extends more towards the root of the tooth lingually than buccally. This uneven distribution of the enamel in the upper and lower teeth is similar in the teeth of *Huerfanodon*, and is seen less so in *Wortmania*, *Onychodectes* and *Conoryctella*. As for the lower molars, *Conoryctes* has more cuspids on the talonid than *Onychodectes* and *Conoryctella*. These cuspids include one proximally (pre-entoconulid) and one distally (post-entoconulid) relative to the entoconid, and small cuspids in the hypoconulid position. *Conoryctes* is different from *Psittacotherium* because the latter is bigger, lacks a continuous stylar shelf on the upper molars, has more cuspids in the talonid of lower molars, and has a higher level of hypsodonty. *Conoryctes* is different from *Ectoganus* because the latter has more hypsodont canines, a distolingual cusp on the upper molars, lacks a continuous stylar shelf and the lower molars are almost square. *Conoryctes* differs from *Stylinodon* since it does not have square-shaped ever-growing teeth and the most anterior premolars are not larger than the posterior premolars. *Conoryctes* differs from *Conoryctella* and *Onychodectes* in having a relatively more robust humerus. The pronator crest on the distal epiphysis of the radius extends anteriorly in *Conoryctes*, whereas it is less protruding in *Wortmania*.

### Species

*Conoryctes comma* Cope 1881 (= *Hexodon molestus* Cope, 1884) [11].

### Type

AMNH 3395, partial left mandible with worn p5, m1 and worn m2, alveolus for p1, roots for p4 and m3, isolated lower canine (Fig 1).

### Type locality

Torrejonian strata of the Nacimiento Formation, San Juan Basin, New Mexico.

### Diagnosis

Same as for genus.

## Comparative description

### Vertebrae

Many of the newly studied specimens (NMMNH P-48052, NMMNH P-79457, NMMNH P-47700, NMMNH P-21509 and NMMNH P-48198) have vertebrae, but most of them are damaged and incomplete.

**Fig 3:**
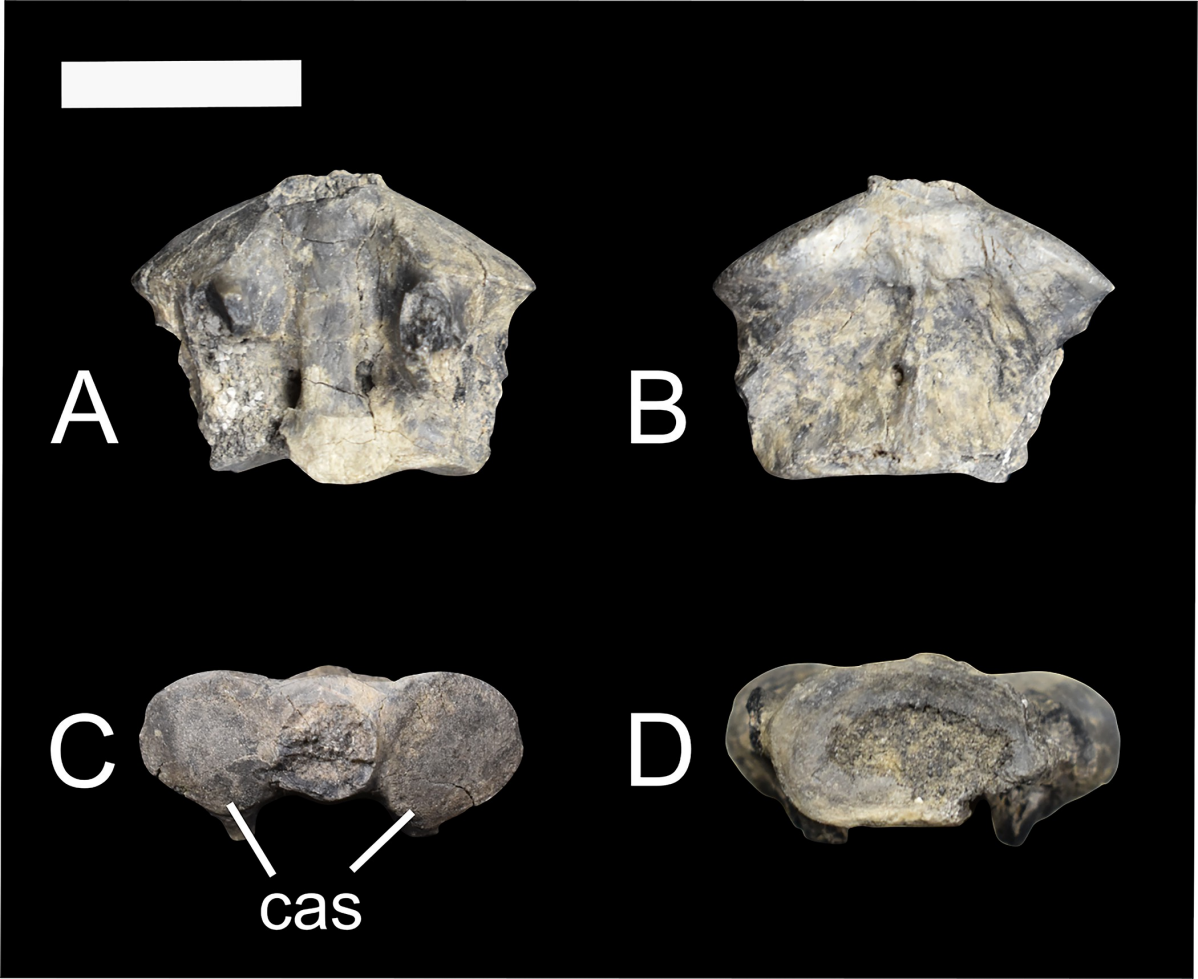
The axis of *Conoryctes comma* (NMMNH P-48052) in dorsal (A), ventral (B), cranial (C) and caudal (D) views. cas: cranial articular surface. Scale bar is 1cm.

The body of the axis (NMMNH P-48052) is short anteroposteriorly; the anteroposterior length is 12.57mm, while the mediolateral width is 16.33mm. Cranially, the two articular processes that connect the axis to the atlas are circular and convex (Fig 3). Between these two processes the dens extension is broken off in NMMNH P-48052. In dorsal view, there are a pair of transverse foramina posteriorly.

**Fig 4:**
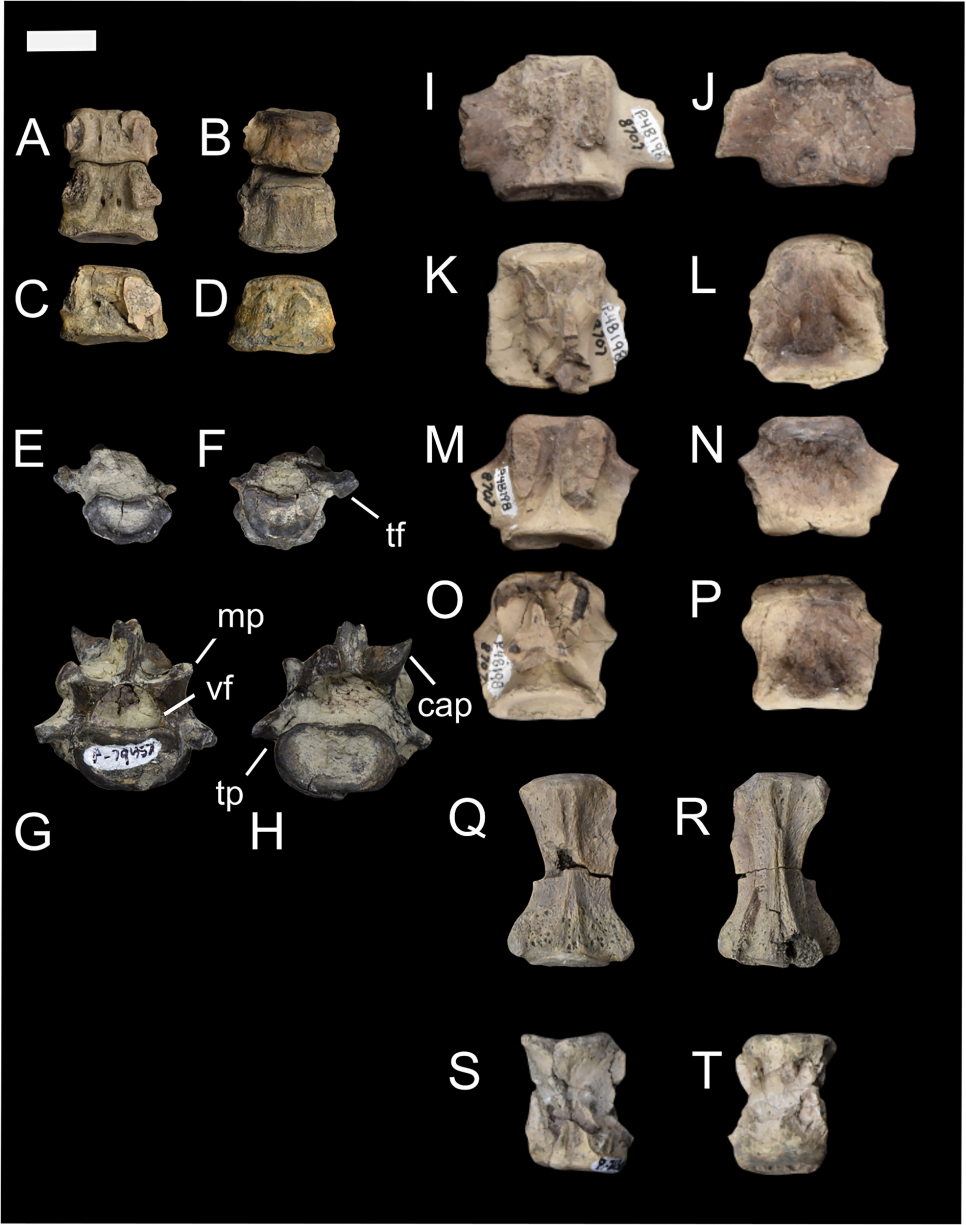
Vertebrae of *Conoryctes comma* [NMMNH P-48052 (A–D, Q, R), NMMNH P-79457 (E–H), NMMNH P-48198 (I–P), NMMNH P-21509 (S, T)]. Part of the neck with cervical vertebrae (A–D), a thoracic vertebra in anterior and posterior views (E, F), a lumbar vertebra in posterior and anterior views (G, H), proximal caudal vertebrae (I–P) in dorsal (I, K, M, O) and ventral (J, L, N, P) views, and distal caudal vertebrae (Q–T) in dorsal (Q, S) and ventral (R, T) views. cap: caudal articular process, mp: mamillary process, tf: transverse fovea, tp: transverse process, vf: vertebral foramen. Scale bar is 1cm.

**Fig 5:**
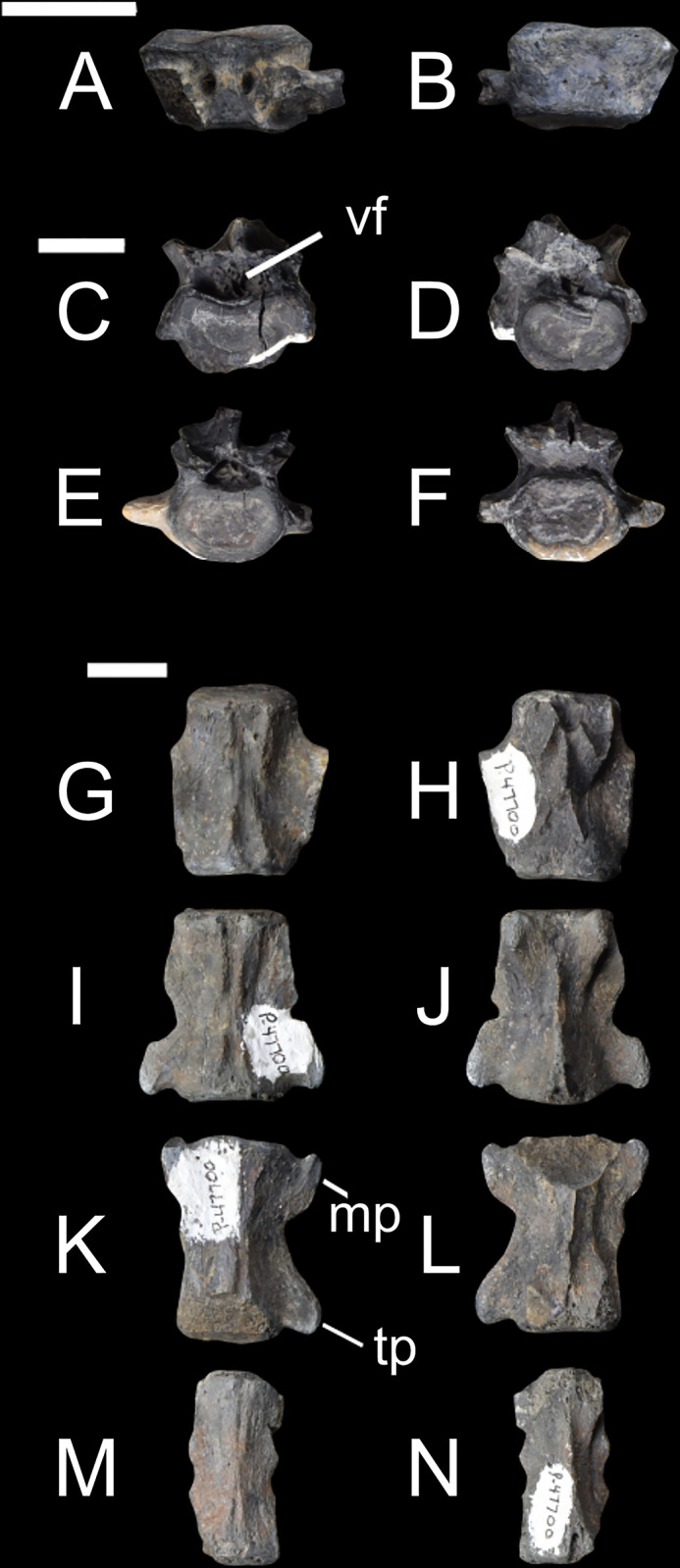
Vertebrae of *Conoryctes comma* (NMMNH P-47700). Cervical vertebra (A–B) in dorsal (A) and ventral (B) views, two lumbar vertebrae (C–F) in anterior (C, E) and posterior (D, F) views, and caudal vertebrae (G–N) from the most proximal to the most distal in dorsal (G, I, K, M) and ventral (H, J, L, N) views. mp: mamillary process, tp: transverse process, vf: vertebral foramen. Scale bar is 1cm.

The posterior cervical vertebrae, as seen in Figs 4 and 5 (NMMNH P-48052, NMMNH P-79457, NMMNH P-47700) have a shorter anteroposterior length compared to mediolateral width (S3 Table). Most of the body of the cervical vertebra is preserved, and it is more mediolaterally wide than dorsoventrally long. Based on these specimens, and particularly the individual NMMNH P-47700, *Conoryctes* had a short neck relative to the rest of its body (Fig 4). The anteroposterior length of the cervical vertebrae is almost half as long as the thoracic and lumbar vertebrae (S3 Table).

NMMNH P-79457 has two thoracic vertebrae (Fig 4E and 4F) with a well-distinguished saddle-shaped body. In anterior and posterior views, there is a small anterior and posterior costal fovea in the body. In lateral view, there is a mammillary process with a circular transverse fovea ventrally, for attachments with the tubercle of the rib. Although the transverse process is missing, and these two thoracic vertebrae are not complete, there is no evidence of an extra articulation between the vertebrae. This extra bony interlocking of the posterior thoracic and lumbar vertebrae is a signature feature in Xenarthra [48]. Specimens NMMNH P-79457 and NMMNH P-48052 have many fragments of ribs, with no sign of pachyostosis. NMMNH P-48052 also has a partial body of the rib that was attached to the costal arch towards the sternum.

NMMNH P-47700 has a lumbar vertebra (Fig 5C–5F), with a less saddle-shaped body and a large vertebral foramen. The base of the mamillary processes extends more dorsally than mediolaterally. The base of the spinous process is also preserved while the transverse processes are incomplete. More posterior lumbar vertebrae are known from NMMNH P-79457 (Fig 4F–4H). The lumbar vertebra of NMMNH P-79457 has anterolaterally-extended transverse processes. In lateral view, the mamillary processes and the spinous process are broken, but the posterior articular process is present. The articular surface is convex and is ventrolaterally-oriented compared to the body.

**Fig 6:**
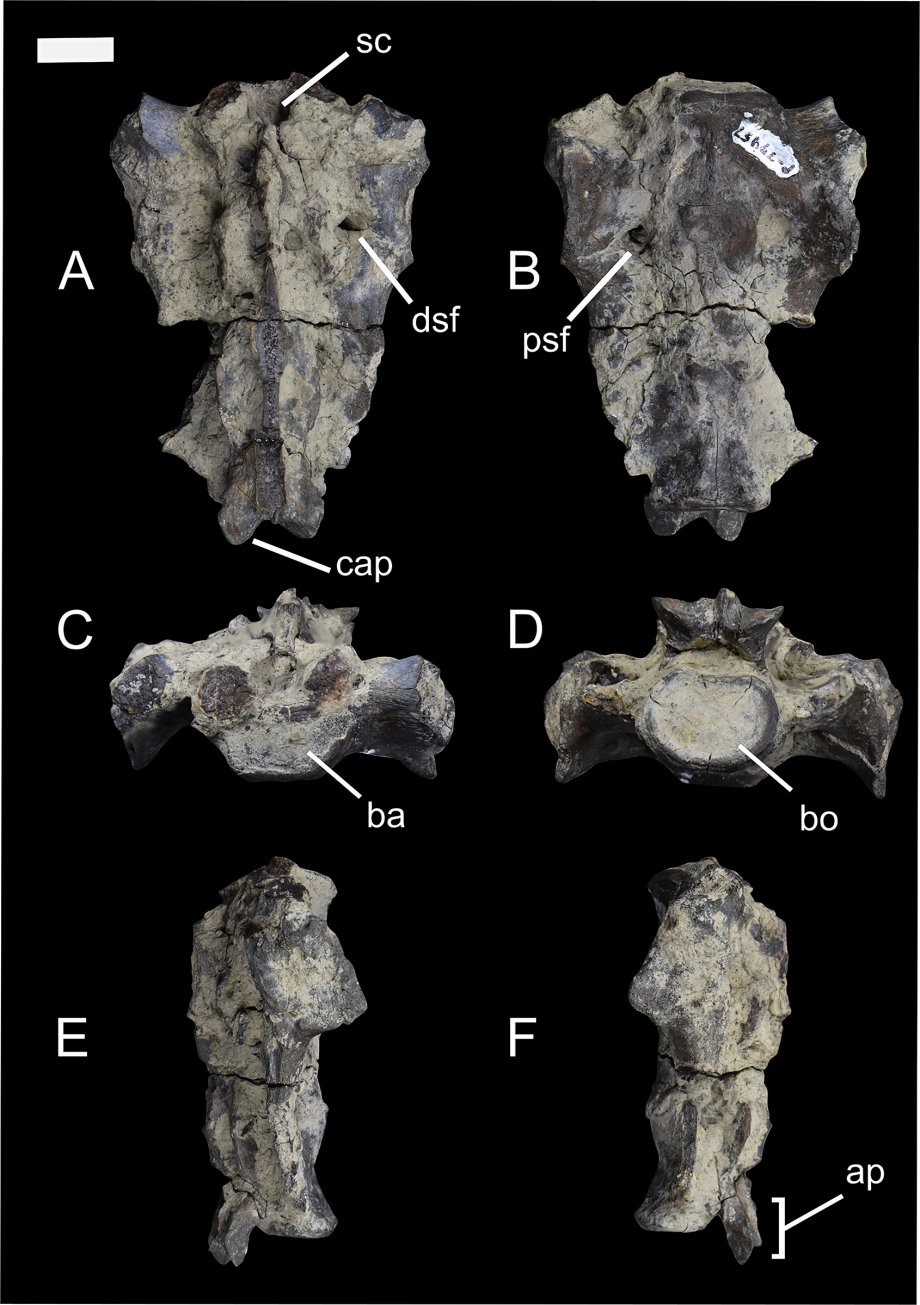
The sacrum of *Conoryctes comma* (NMMNH P-79457) in dorsal (A), ventral (B), anterior (C), posterior (D), medial (E) and lateral (F) views. ap: apex, ba: base, bo: body, cap: caudal articular process, dsf: dorsal sacral foramina, psf: pelvic sacral foramina, sc: sacral canal. Scale bar is 1cm.

The sacrum of *Conoryctes* is known from NMMNH P-79457 (Fig 6). It is composed of three fused sacral vertebrae; the most anterior is short anteroposteriorly, while the second and third sacrals are comparatively longer. In cranial view, the base of the sacrum is almost flat and is more mediolaterally-elongate than circular. Dorsal to the base is the sacral canal and ventrally the promontory. In anterior aspect, the two cranial articular processes, mediolateral to the sacral canal, are broken. The sacral canal is large and circular. There are well-preserved dorsal and ventral sacral foramina through which the spinal nerves and vessels passed. The wings of the sacrum are broken, but in medial view there is evidence for an articular surface for the attachment with the ilium (Fig 6E). In posterior view (Fig 6D) the sacral body is almost circular.

Some caudal vertebrae (NMMNH P-48198, NMMNH P-47700, NMMNH P-48052 and NMMNH P-21509) are preserved (Figs 4I–4T and 5G–5N). The body of the caudal vertebrae are cylindrical, and the transverse processes extend posterolaterally. In dorsal view, there is a similar triangular bulging near the caudal epiphysis of the body, as seen in the cervical vertebrae. The vertebral arch becomes smaller posteriorly and in more distal caudal vertebrae the mamillary processes are smaller. The more distal caudal vertebrae (NMMNH P-48198 and NMMNH P-48052) have a smaller foramen posteriorly for the nerves and blood vessels.

The total number of vertebrae of *Conoryctes* remains unknown; however, based upon the morphology of the preserved vertebrae (Figs 4 and 5), it had a short and robust neck and probably a long tail. This structure has been proposed for *Onychodectes* by Schoch [6] and is also seen in the FMNH PM 3895 specimen of *Stylinodon*. *Ectoganus* (USGS 3838) has also been proposed as having a long tail [6]. There are only two other sacral elements known for taeniodonts, including specimens of *Onychodectes* (AMNH 16410) and *Stylinodon* (FMNH PM 3895). Both specimens have partially preserved sacra, but the number of fused sacral vertebrae is unknown. The specimen of *Onychodectes* (AMNH 16410) is incomplete; only part of the first sacral and part of the wings are preserved. The sacrum of *Conoryctes* (NMMNH P-79457) is larger than *Onychodectes*, while both have a mediolaterally-elongate base of the sacrum and wide wings. Turnbull [7] described the incomplete sacrum of *Stylinodon* (FMNH PM 3895) and hypothesised it consisted of three sacrals.

*Conoryctes*, as well as *Escavadodon* (NMMNH P-22051), had a relatively long tail [34]. The sacrum of *Conoryctes* has three fused vertebrae, distinct from the four fused sacral vertebrae of leptictids [30]. The lumbar vertebrae of *Conoryctes* have anterolaterally-extended transverse processes, which is similar to *Leptictis* [33] and *Periptychus* (NMMNH P-47693), but unlike the posterolaterally-projecting transverse processes of *Pantolambda* (AMNH 16663) [16, 35, 36, 49].

### Humerus

**Fig 7:**
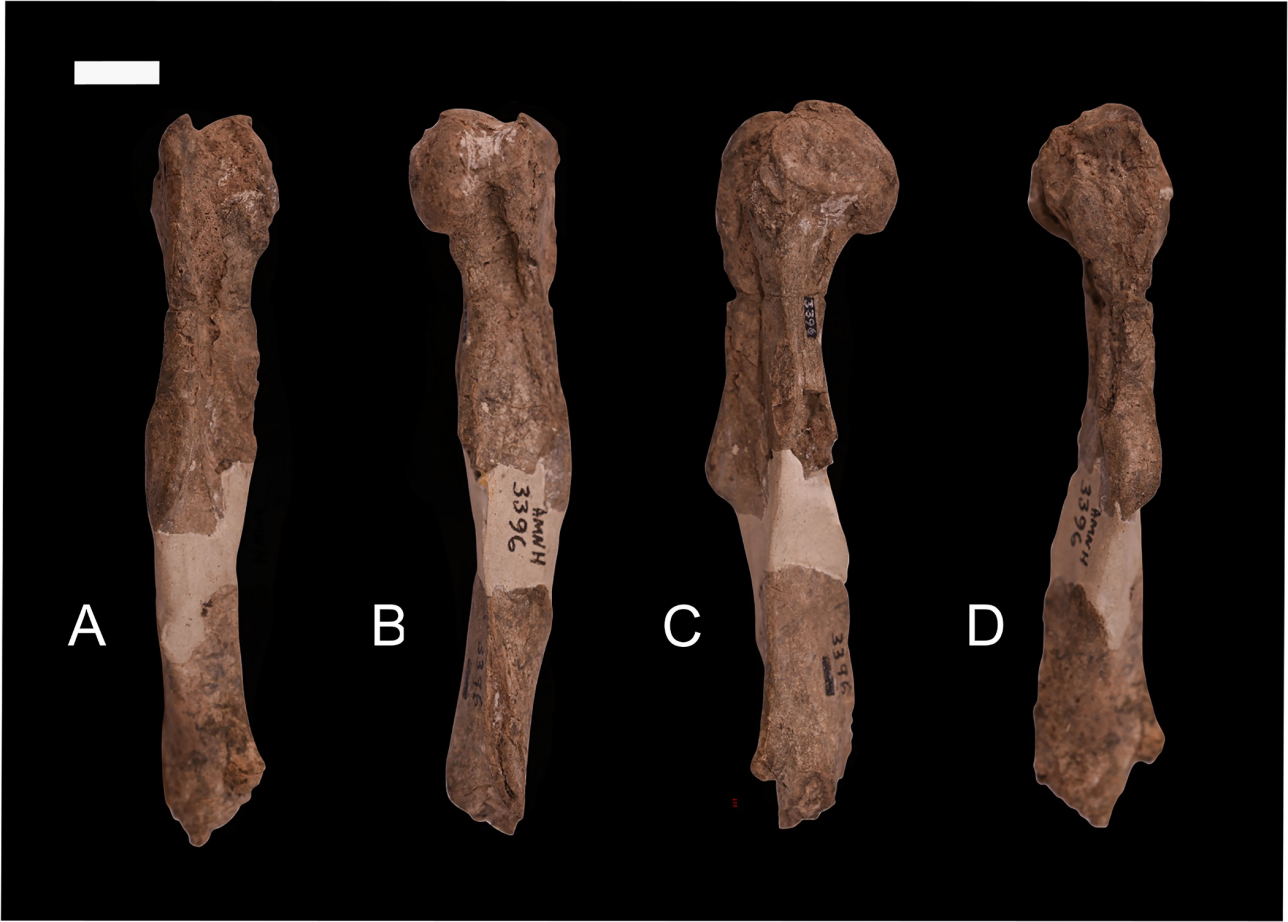
A humerus previously associated with *Conoryctes comma* (AMNH 3396), but which probably represents a different taxon, in anterior (A), posterior (B), medial (C) and lateral (D) views. Scale bar is 1cm.

Schoch [6] described and referred a partial humerus and radius associated with AMNH 3396 to *Conoryctes comma*. AMNH 3396 includes upper and lower jaws and teeth collected by David Baldwin from an unspecified location within the San Juan Basin for Cope in the early 1880s. It became the lectotype of *Hexodon molestus* [11] but was later synonymized with *Conoryctes comma* [13]. The partial right humerus of AMNH 3396 (Fig 7) is comprised of two fragments; a proximal end that extends to the end of the deltopectoral crest and a more distal portion of the shaft. The two portions are joined by a significant bridge of plaster. Schoch [6] described the humerus as being nearly identical in size and morphology to that of *Oncychodectes* (AMNH 16410) “… except that in *Conoryctes* the deltoid ridge is flattened and slightly broadened in the middle of the shaft rather than coming to a high anterior point as in *Onychodectes*” [6, p. 40].

Schoch [6] accepted the association of the humerus with the dentition of AMNH 3396 as sufficient to show that they belonged to the same taxon, and this undoubtedly was influential in his interpretation of *Conoryctes* as being “primitive with few specializations” [6, p. 159]. However, we here argue that this partial humerus probably does not belong to *Conoryctes* and that *Conoryctes* is much more derived in its postcranial adaptations than has been previously described.

First, elements of the forearm associated with other specimens of *Conoryctes*, such as the ulna, metacarpal II, and unguals as we describe below have a morphology more consistent with it having a forelimb adapted for powerful motions consistent with scratch-digging as is seen in stylinodontid taeniodonts. Secondly, two partial humeri recovered from strata of the Nacimiento Formation of the San Juan Basin that also contain *Conoryctes* are similar in terms of robusticity, size and development of the deltopectoral crest, and morphology of the distal end to that of the stylinodontid taeniodont *Psittacotherium* but are much smaller and of a size expected for *Conoryctes*. Although these specimens were not associated with diagnostic dental remains, the humeri are comparable with *Conoryctes*. Moreover, no other early Paleocene mammal of this size is hypothesized to have such extreme adaptations of the forelimb.

**Fig 8:**
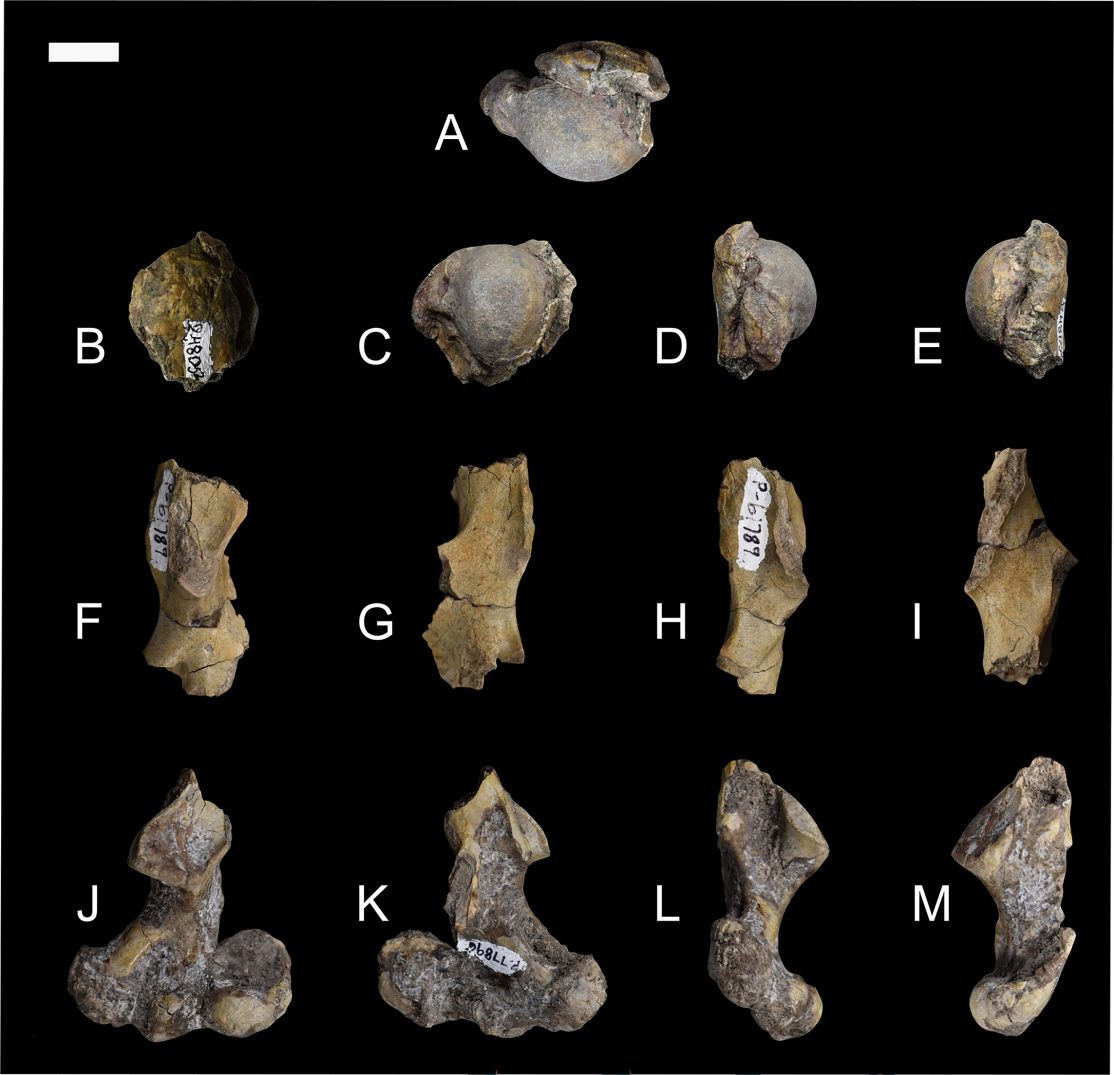
The humerus of *Conoryctes comma* [NMMNH P-48052 (A–E), NMMNH P-61789 (F–I) and NMMNH P-77896 (J–M)] in proximal (A), anterior (B, F, J), posterior (C, G, K), medial (D, H, L) and lateral (E, I, M) views. Scale bar is 1cm.

**Fig 9:**
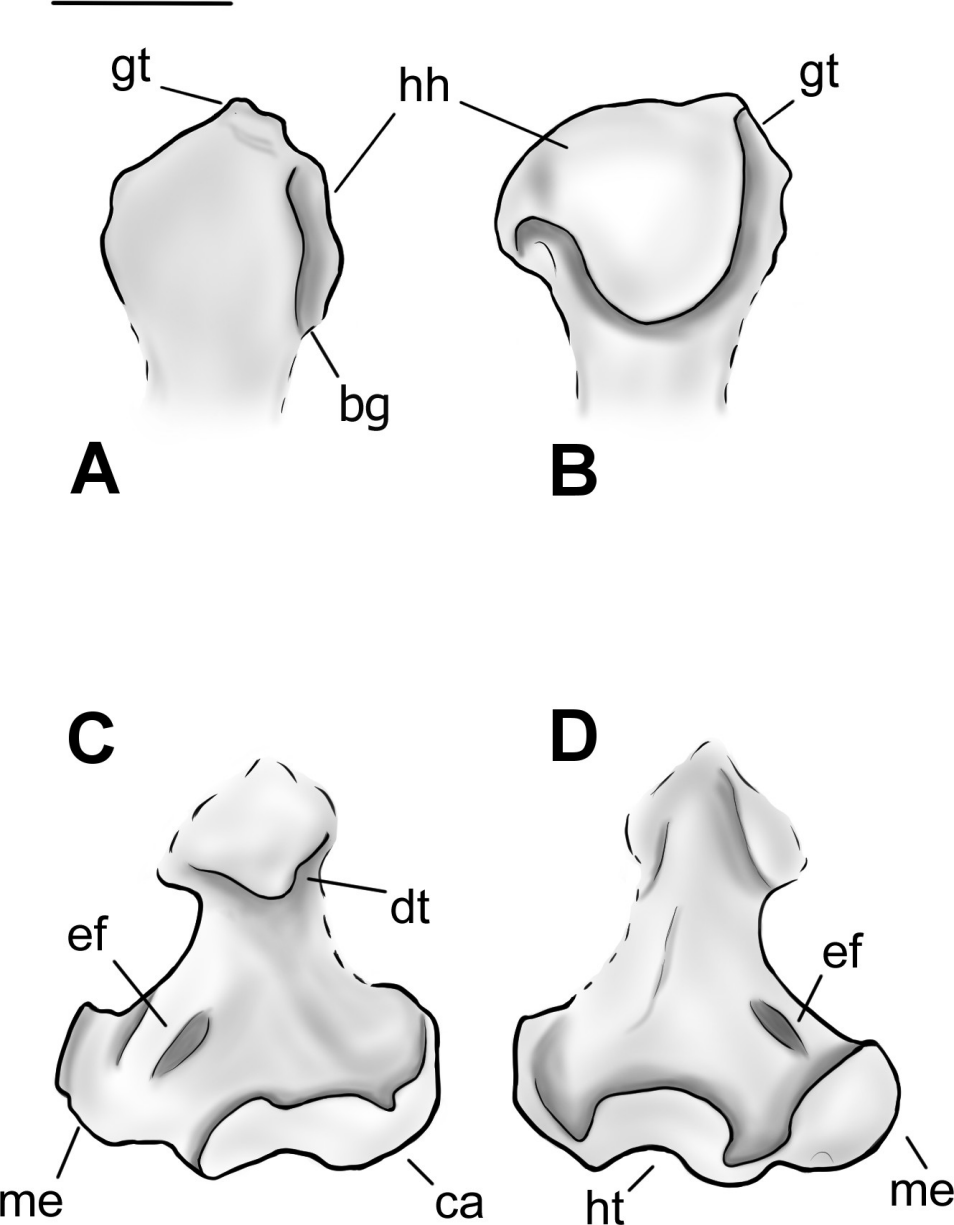
Drawing of the left humerus of *Conoryctes comma* based on NMMNH P-48052 and NMMNH P-77896 in anterior (A, C), and posterior (B, D) views. bg: bicipital groove, ca: capitulum, dt: deltopectoral crest, ef: entepicondylar foramen, gt: greater tubercle, hh: humeral head, ht: humeral trochlea, me: medial epicondyle. Scale bar is 1cm.

NMMNH P-48052, NMMNH P-61789, and NMMNH P-77896 provide new information about the humerus of *Conoryctes comma* (Fig 8). Proximally the humerus of *Conoryctes* has a distinguished greater tubercle which is approximately at the same level as the humeral head (NMMNH P-48052, Figs 8 and 9). There is a lip for the bicipital groove separating the head from the greater tubercle and extending anterodistally (Fig 9A). The humeral head is complete, with an almost hemispherical shape, being longer proximodistally than mediolaterally wide. The lesser tubercle is close to the anteromedial surface of the humeral head. In anterior view, the lesser tubercle is separated from the greater tubercle with a deep bicipital groove.

The shaft of the humerus is robust with prominent crests and flanges. Proximally, there is a well-defined deltopectoral crest. The edges of the crest are strong, forming a U-shaped deltopectoral region and a prominent deltopectoral tuberosity that protrudes from the shaft to a large degree (Fig 8F–8M). The deltopectoral tuberosity extends to the level of the epicondylar crest (NMMNH P-61789 and NMMNH P-77896). In anterior view, medial to the deltopectoral region, there is a crest for the insertion of the teres major and the latissimus dorsi muscles. The teres major muscles were attached to the lateral border of the scapula and were responsible for the extension and medial rotation of the humerus [39]. The latissimus dorsi also assisted in the rotation and extension of the shoulder [39]. This prominent crest in *Conoryctes* indicates a strong shoulder joint. The lateral epicondylar crest, where the extensor carpi radialis muscles were attached, is lateromedially broad, giving a robust shape of the humerus. Distally, on the medial side, the supracondylar crest is mediolaterally-expanded and there is a large entepicondylar crest (Fig 8J). The proximodistal distance between the deltopectoral tuberosity and the proximal border of the entepicondylar foramen is short.

The distal epiphysis of *Conoryctes* is mediolaterally broad (NMMNH P-77896, Figs 8J–8M and 9C, 9D). The distal extent of the epicondylar crest extends laterally and it reaches distally near the distal level of the deltopectoral tuberosity. The large area of the epicondylar crest is where the extensor muscles, the radial collateral ligament and tendons attached, providing stability to the elbow joint [39]. The olecranon fossa is deep but lacks an opening. The humeral trochlea is deep, and its mediolateral width is equal to the width of the capitulum, in anterior view. The capitulum is more spherical in anterior view and flatter in posterior view. In posterior view, the trochlear keels are subequal in size.

The humerus is known for other taeniodonts including *Onychodectes* (AMNH 16410), *Ectoganus* (FMNH P 26090), *Psittacotherium* (TMM 41364–1, NMMNH P-48358) and *Stylinodon* (YPM 11096) [6, 7]. There are many similarities between the humerus of *Conoryctes* and *Onychodectes*. Both animals have broader distal than proximal epiphyses and the greater tubercle extends more anteriorly. However, *Onychodectes* has a more slender diaphysis than *Conoryctes*. The deltopectoral region is broader in *Conoryctes* than in *Onychodectes* and the deltopectoral tuberosity is more robust. The deltoid crest extends more distally in *Conoryctes* than in *Onychodectes*. The humerus of *Conoryctes* more closely resembles the humeri of the more derived taeniodont *Psittacotherium*, known also from the San Juan Basin, in being robust with a broad deltopectoral region and mediolaterally-broad epicondylar crests. *Ectoganus* and *Stylinodon* have an even broader humeri with more mediolaterally-extending deltopectoral regions than *Conoryctes*. In addition to being almost six times larger, the humerus of *Stylinodon* has a deltopectoral crest that expands more laterally than in *Conoryctes*.

*Conoryctes* shares similarities with the humerus of *Escavadodon* (NMMNH P-22051) [34]. The greater tuberosity is almost on the same level as the humeral head and the deltopectoral crest and tuberosity are similar in both taxa, extending distally near the level of the epicondylar crest [34]. However, *Conoryctes* has a relatively more slender humerus than *Escavadodon*. The teres major tubercle is less prominent in *Conoryctes* than in *Escavadodon* and the humeral trochlea is equal in mediolateral width to the capitulum. The humeral trochlea is placed central to the mediolateral width of the distal epiphysis in *Conoryctes*, whereas it is more laterally-placed in *Escavadodon*.

The humerus of *Conoryctes* shares some similarities, but also exhibits differences, to *Periptychus* (NMMNH P-47693) and *Pantolambda* (AMNH 16663) [16, 35, 49]. In all three taxa, the greater tubercle is on the same level as the humeral head. The humeral head is proximodistally shorter relative to the humeral shaft in *Conoryctes* than in *Periptychus* and *Pantolambda*. The deltopectoral region is more prominent in *Conoryctes* and *Pantolambda* than in *Periptychus* [16, 35, 49]. All three taxa have a moderately deep humeral trochlea that is medially-expanded in *Conoryctes* and *Periptychus* and less so in *Pantolambda*.

### Ulna

**Fig 10:**
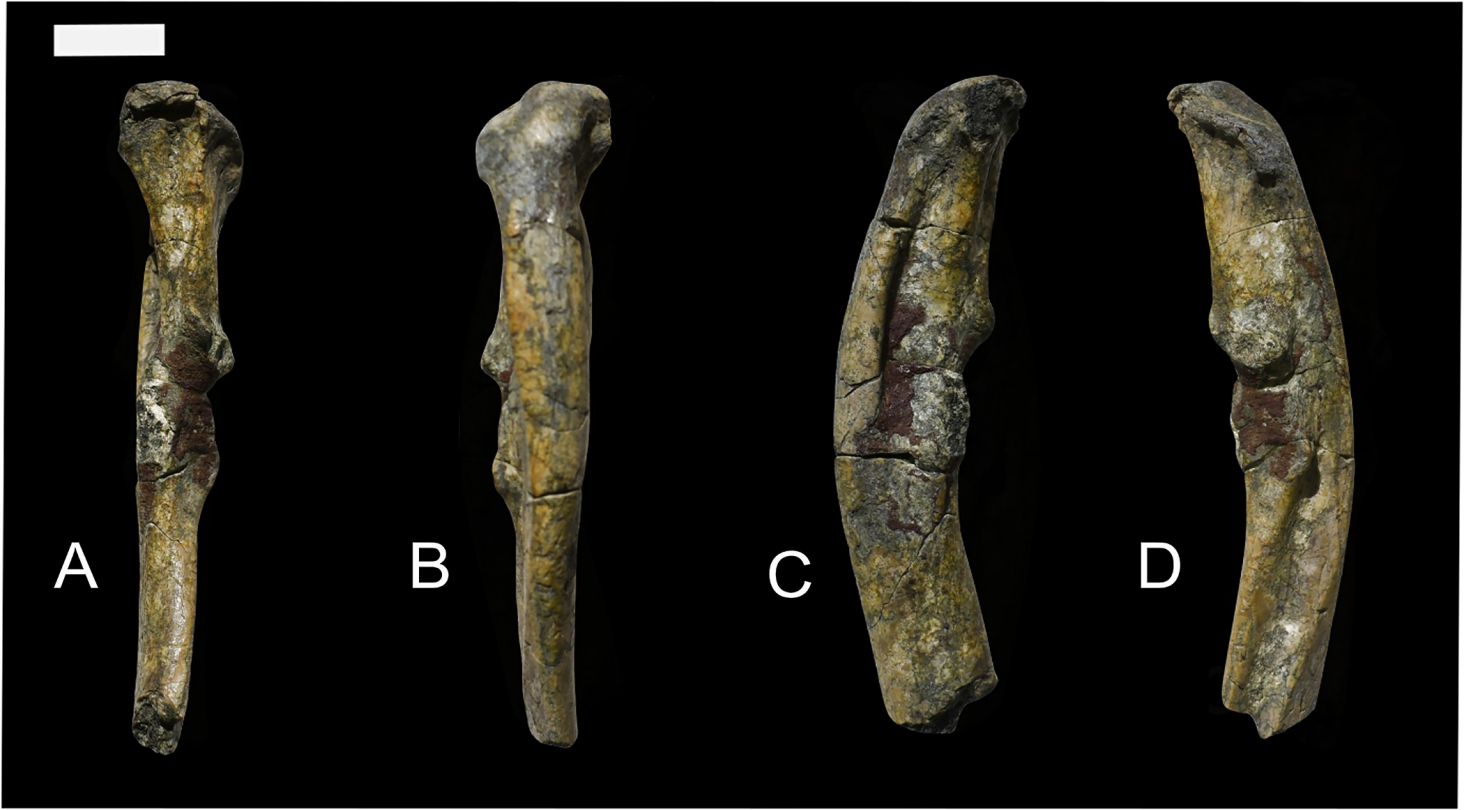
The left ulna of *Conoryctes comma* (NMMNH P-48052), in anterior (A), posterior (B), medial (C) and lateral (D) views. Scale bar is 1cm.

**Fig 11:**
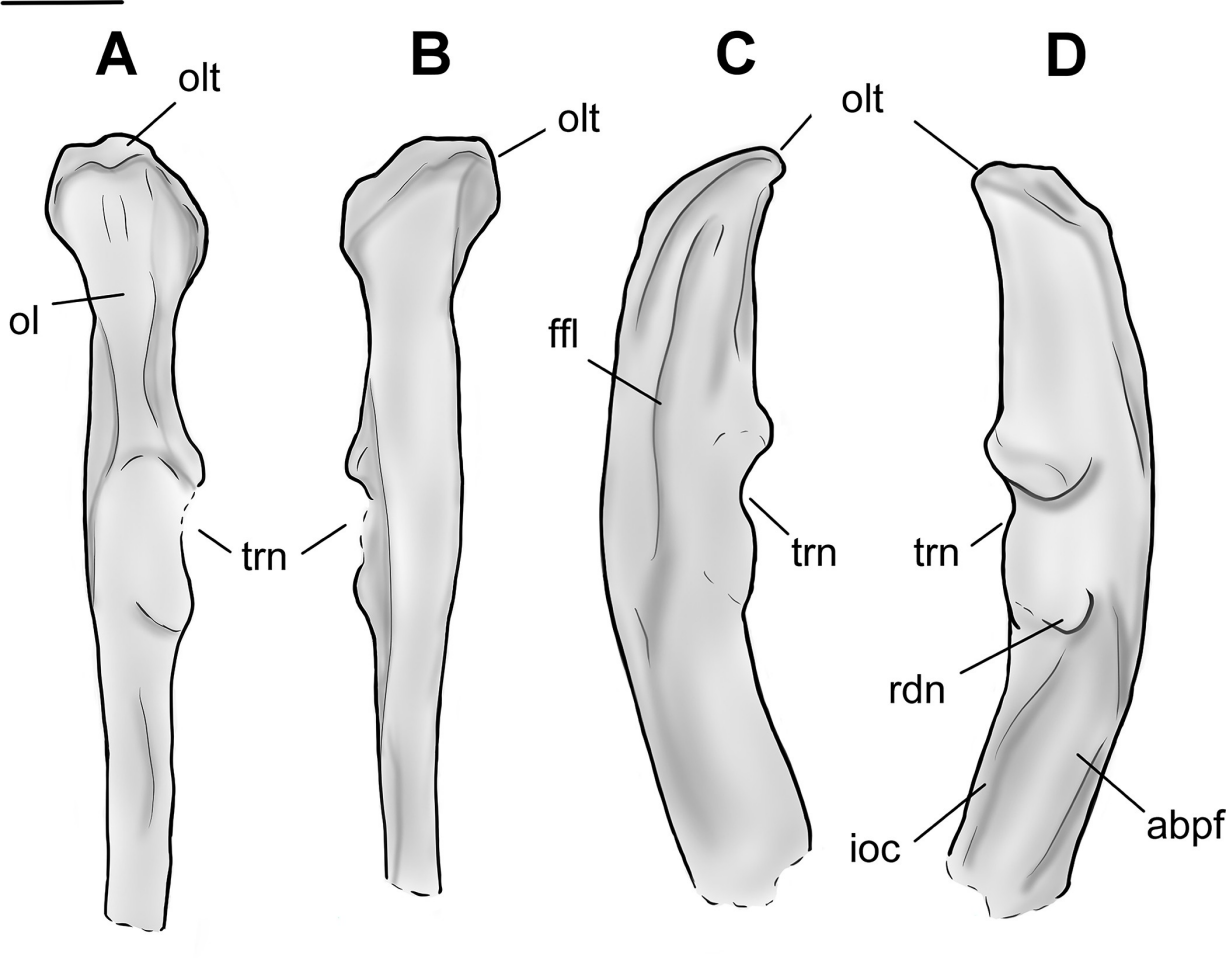
Drawing of the left ulna of *Conoryctes comma* based on NMMNH P-48052, in anterior (A), posterior (B), medial (C) and lateral (D) views. adpf: abductor pollicis longus fossa, ffl: flexor fossa, ioc: interosseous crest, ol: olecranon, olt: olecranon tuber, rdn: radial notch, trn: trochlear notch. Scale bar is 1cm.

Specimen NMMNH P-48052 has fragments of a proximal ulna (Figs 10 and 11). The olecranon of *Conoryctes* is almost complete, missing only a small part of the anteromedial end. In anterior view, the olecranon is wider mediolaterally than the rest of the shaft and is oriented laterally (Fig 10). There is a longitudinal ridge dividing the olecranon in half, creating medial and lateral fossae. The medial fossa is shallower but subequal in size to the lateral fossa. In the medial fossa of the olecranon attached the medial head of the triceps brachii, the tensor fasciae antebrachia, the flexor carpi ulnaris and the medial flexor digitorum profundus muscles [39]. In the lateral fossa, the anconaeus muscle, the lateral flexor digitorum profundus and the lateral head of the triceps brachii were attached [39].

In lateral view, distal to the olecranon process, there is the elongate anconeal fossa that extends distally, forming a ridge posteriorly (Fig 10D). Similarly, in medial view there is an even deeper elongate flexor fossa. The posterior ridge of the flexor fossa is strong and continues distally until the proximodistal end of the humerus-ulna articular region, meeting the proximal ulnar diaphysis.

In NMMNH P-48052, the ulnar trochlear and the articulation surfaces for the humerus and the radius are damaged. Part of the anconeal process is preserved, showing that it might have been asymmetrical and more elevated medially. The trochlear notch is concave, and the radial notch forms a concave and large area for articulation with the radius. The radial notch was adjacent to the coronoid process laterally. Almost at the distal end of the radial notch, in lateral view, there is a prominent longitudinal fossa for the flexor digitorum profundis and lateral triceps brachii muscles [39]. Alongside this fossa there is another, more shallow, longitudinal fossa, providing attachment for the abductor pollicis longus muscle [39] (Fig 11).

There are many ulnae known for almost all the genera of Taeniodonta, i.e., *Onychodectes* (AMNH 16410), *Conoryctella* (UNM B-1258, new label NMMNH P-25056), *Wortmania* (NMMNH P-19460), *Psittacotherium* (AMNH 2453 and AMNH 16560), *Ectoganus* (USGS 3838 and FMNH P 26083) and *Stylinodon* (YPM 11096, cf. *Stylinodon mirus* USNM 18425, UW 2270 and FMNH PM 3895) [6, 7]. The ulna of *Conoryctes* is similar to those of *Conoryctella* and *Wortmania*, having a concave posterior surface. The olecranon protrudes medially and is posterolaterally inclined in *Conoryctes*, *Onychodectes* and *Conoryctella*. The radial notch is flatter in *Conoryctes* and *Conoryctella* than in *Onychodectes*. In these three species, the radial notch is more laterally placed than in *Wortmania*, which has a more concave radial notch. In medial view, the flexor fossa on the olecranon is deep and well-defined in *Conoryctes*, *Conoryctella* and *Wortmania*, and less so in *Onychodectes*. In lateral view, the two longitudinal fossae in *Conoryctes* are similar to those of *Onychodectes*, with the most anterior one starting from the middle of the articular region and the posterior one more distally.

The ulna of *Conoryctes* is similarly curved posteriorly as in *Ectoganus* (USGS 3838) whereas in *Stylinodon* (YPM 11096) the ulna is more significantly curved [6, 7]. The proximal olecranon in *Conoryctes* protrudes less medially than in *Stylinodon*; both animals have a strong longitudinal crest on the olecranon of the ulna. *Conoryctes* has a shorter olecranon than the articular surface, opposite to *Stylinodon*. The radial notch is more laterally-placed in *Conoryctes*. In *Psittacotherium*, *Ectoganus* and *Stylinodon* the radial notch is almost completely anteriorly-placed. In medial view, the flexor fossa is not as prominent in *Conoryctes* and *Psittacotherium*, while the fossa and posterior ridge are more robust in *Stylinodon*.

The ulna of *Conoryctes* has a different shape than that of *Escavadodon* (NMMNH P-22051), as the olecranon of the latter is strongly medially-inflected [34]. The olecranon of *Conoryctes* is shorter proximodistally than in *Escavadodon*. The radial notch of *Conoryctes* is placed more laterally, like in palaeanodonts and unlike *Escavadodon*. In lateral view, the most anterior fossa of the shaft is very prominent in *Conoryctes*, similar to *Escavadodon* and leptictids. In medial view, the flexor fossa is deep with a prominent ridge in *Conoryctes*, similarly so in *Escavadodon* [34].

Compared to *Periptychus* (NMMMNH P-53998, NMMNH P-35194, NMMNH P-47693) and *Pantolambda* (AMNH 16663), the ulna of *Conoryctes* is less robust [16, 35, 49]. On the olecranon, *Conoryctes* and *Periptychus* have a well-defined ridge anteriorly that continues proximally creating two fossae for muscle attachment; this feature is not as pronounced in *Pantolambda*. Unlike *Conoryctes*, the olecranon of *Periptychus* is almost subequal in proximodistal length to the articular surface. The radial notch of *Conoryctes* is more laterally-placed, while *Periptychus* and *Pantolambda* have an anteromedially-placed radial notch. In medial view, the flexor fossa of *Periptychus* is deep with a larger ridge than in *Conoryctes*, and shallow in *Pantolambda*. In lateral view, the two longitudinal fossae are similar in size and shape in *Conoryctes* and *Periptychus*. In *Pantolambda* the lateral fossae are less-defined and extend less distally.

### Radius

**Fig 12:**
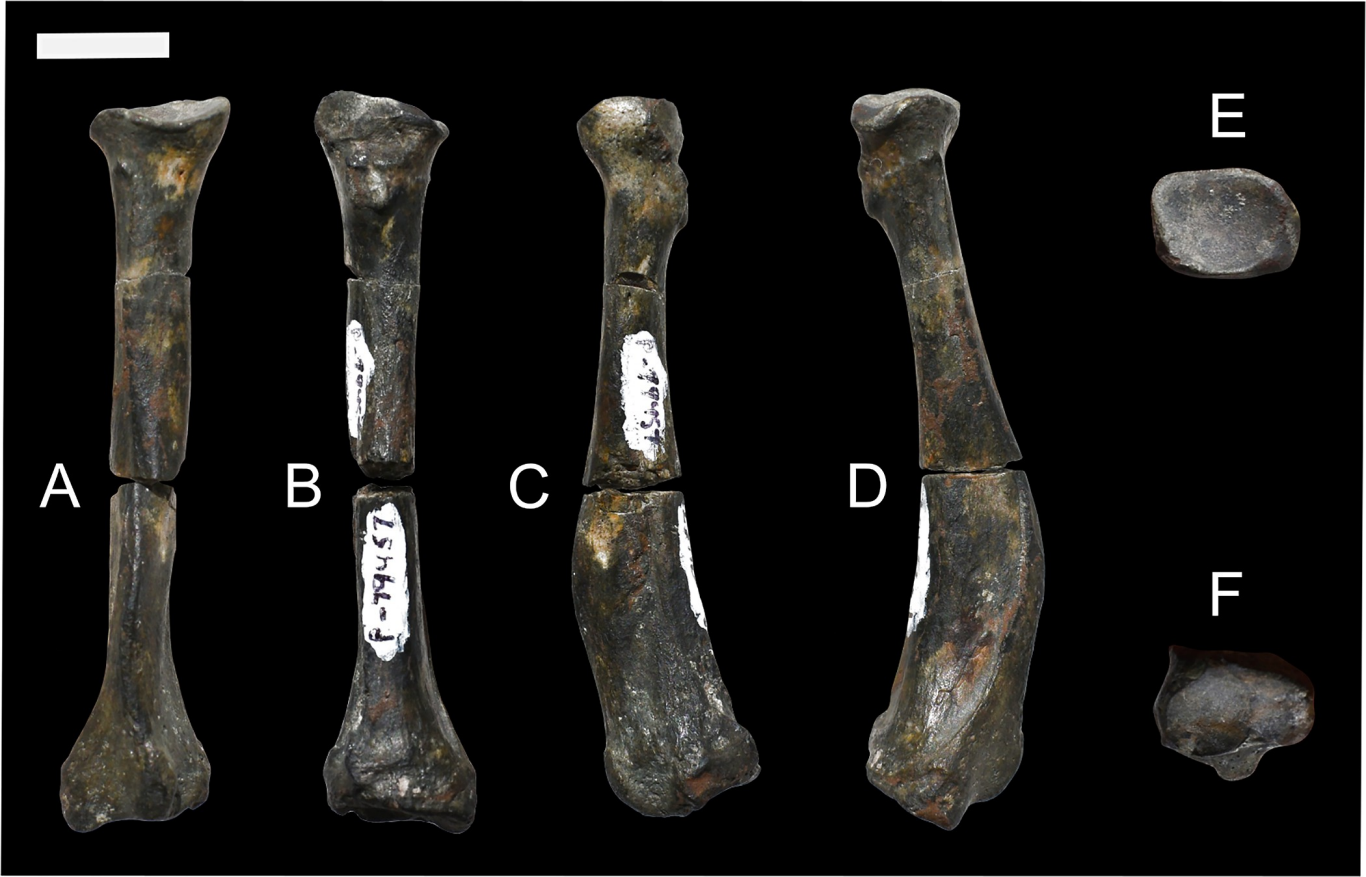
Radius of *Conoryctes comma* (NMMN P-79457) in anterior (A), posterior (B), medial (C), lateral (D), proximal (E) and distal (F) views. Scale bar is 1cm.

**Fig 13:**
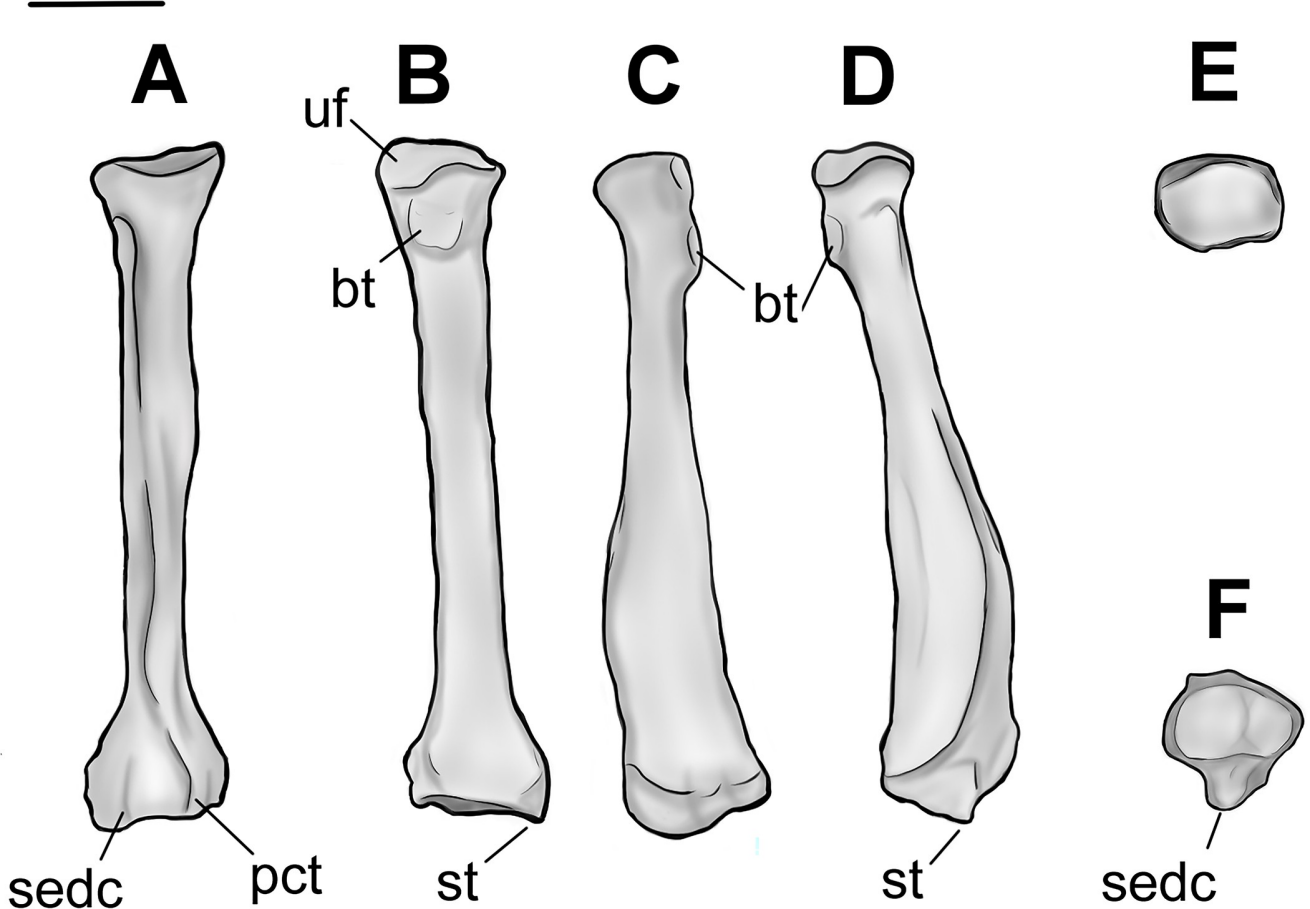
Drawing of the left radius (NMMN P-79457), *Conoryctes comma*, in anterior (A), posterior (B), medial (C), lateral (D), proximal (E) and distal (F) views. bt: bicipital tuberosity, pct: pronator crest, sedc: sulcus for the extensor digitorum communis, st: styloid process, uf: ulnar facet. Scale bar is 1cm.

Specimen NMMNH P-79457 has a complete, well preserved left radius of *Conoryctes* (Figs 12 and 13). The proximal diaphysis of the radius is broader mediolaterally than anteroposteriorly deep. In proximal view, the head of the radius is almost square (Fig 12E). The mediolateral width of the proximal epiphysis is greater than the anteroposterior depth (S6 Table). The deepest point of the capitular articular surface is in the middle of the fossa and the rims are continuous. In anterior view, the radial head bulges over the neck, while the posterior and lateral parts of the capitular articular surface reach more proximally than the rest of the circumference of the fossa. Therefore, the radial head of *Conoryctes* is medially inclined. The capitular eminence, in medial view, is oriented posteromedially, creating a large lip over the radial neck. In posterior view, there is a flat ulnar facet that is broad both mediolaterally and proximodistally. Distal to this facet is the prominent bulging area of the bicipital tuberosity (Fig 13). The bicipital tuberosity is almost circular and was responsible for the insertion of the tendon of the biceps brachii muscle [39]. In lateral aspect, the radial head and neck are almost continuous.

The proximal radial diaphysis of *Conoryctes* is cylindrical and broadens more anteroposteriorly. In lateral aspect, there is a longitudinal large fossa for the abductor pollicis longus muscle (Fig 13D). The distal diaphysis is flattened mediolaterally and anteriorly forms the pronator crest for the attachment of the pronator teres and the pronator quadratus muscles, giving an anteriorly-curved shape to the radius [39]. In anterior view, the pronator crest is strong, sharp and slightly curved laterally, extending almost 40% of the total length of the radius. Near the distal radial epiphysis, the pronator crest is raised into a proximodistally elongate, prominent tuberosity. Lateral to this tuberosity is a broad and deep sulcus for the extensor digitorum communis and the digitorum lateralis muscles. Medial to the tuberosity is a smaller and shallower sulcus for the tendon of the extensor carpi radialis muscle (Fig 13A).

The distal radial epiphysis of *Conoryctes* is mediolaterally broader with a moderate styloid process (S6 Table). In distal view, there are two fossae that are poorly separated (Fig 12F). The lateral fossa is larger, more than half the distal radial epiphysis, deeper and almost circular. This is the area where the lunate articulated with the radius. The medial fossa is smaller and shallower but also circular for articulation with the scaphoid.

The radii are known for other taeniodonts, such as *Onychodectes* (AMNH 16410) *Wortmania* (AMNH 3394, NMMNH P-19460), *Psittacotherium* (AMNH 16560, NMMNH P-77785), *Ectoganus* (USNM 1001) and *Stylinodon* (YPM 11096 and FMNH PM 3895) [3, 6, 7]. The distal end of the radius is similar in *Conoryctes* and *Onychodectes*. The capitular articular surface, in proximal view, is mediolaterally less elongate in *Conoryctes* and *Onychodectes*, having a slender neck relative to the radial head, than in *Wortmania*. The capitular eminence is more proximally-protruding in the lateral area of the ridge in *Conoryctes* as well as in *Onychodectes* and *Wortmania*. The bicipital tuberosity is more circular in *Conoryctes*, but it is proximodistally-elongate in *Onychodectes* and *Wortmania*. The ulnar facet is strong and proximodistally-broad in *Conoryctes* and *Wortmania* and very small in *Onychodectes*. On the lateral shaft of the radius, the fossa for the abductor pollicis longus muscle in *Wortmania* is deeper and more longitudinally broad than in *Conoryctes*. Moreover, the pronator crest starts more distally relative to the whole radius in *Conoryctes* but proximally in *Wortmania*.

Compared to *Psittacotherium* (AMNH 16560), *Ectoganus* (YPM 39805) and *Stylinodon* (YPM 11096 and FMNH PM 3895), *Conoryctes* has a very slender radius [6, 7]. The radial head is almost circular in *Conoryctes* but is elongate mediolaterally in *Ectoganus* and *Stylinodon*. In anterior aspect, the pronator crest of *Ectoganus* and *Stylinodon* is not as strong as in *Conoryctes* and starts more proximal to the shaft. There are two sulci lateral and medial to the pronator crest, but these originate more proximally in *Ectoganus* and *Stylinodon* than in *Conoryctes*. The lateral fossa for the abductor pollicis longus muscle is shallower in *Conoryctes* compared to *Stylinodon*. The styloid process is more mediolaterally broad in *Stylinodon* than in *Conoryctes*. The lateral fossa for the articulation with the lunate is more prominent, deeper and almost circular, both in *Conoryctes* and *Stylinodon*.

*Conoryctes*, similar to leptictids and *Escavadodon* (NMMNH P-22051), has a relatively more slender radius than palaeanodonts [1, 30, 34]. *Conoryctes* has an almost circular radial head, unlike *Escavadodon* that has an elliptical radial head, with the latter being broader mediolaterally than anteroposteriorly deep. *Conoryctes* has a similarly flat, gently curved ulnar facet compared to that of *Escavadodon* and *Leptictis*. The distal shaft of *Conoryctes* is similar to *Escavadodon* and primitive palaeanodonts; the pronator crest forms after half the proximodistal length ending in a tubercle. The pronator crest of *Conoryctes* is sharp and strong, potentially like in palaeanodonts. *Conoryctes* and *Escavadodon* have a distinct styloid process and, in distal view, these two genera, as well as leptictids and palaeanodonts, have two carpal facets that are poorly-defined.

*Conoryctes*, *Periptychus* (NMMNH P-47693) and *Pantolambda* (AMNH 16663) [16, 35, 49] all have radii that are mediolaterally- broader distally than proximally, and the capitular eminence is positioned more laterally. The ulnar facet is less curved in *Conoryctes* than in *Periptychus*. The pronator crest is large in *Periptychus* leading to a round tuberosity, whereas the crest and the tuberosity are stronger in *Conoryctes* and *Pantolambda*. In distal aspect, the carpal facets are well distinguished in *Periptychus*, unlike *Conoryctes*.

### Manus elements

**Fig 14:**
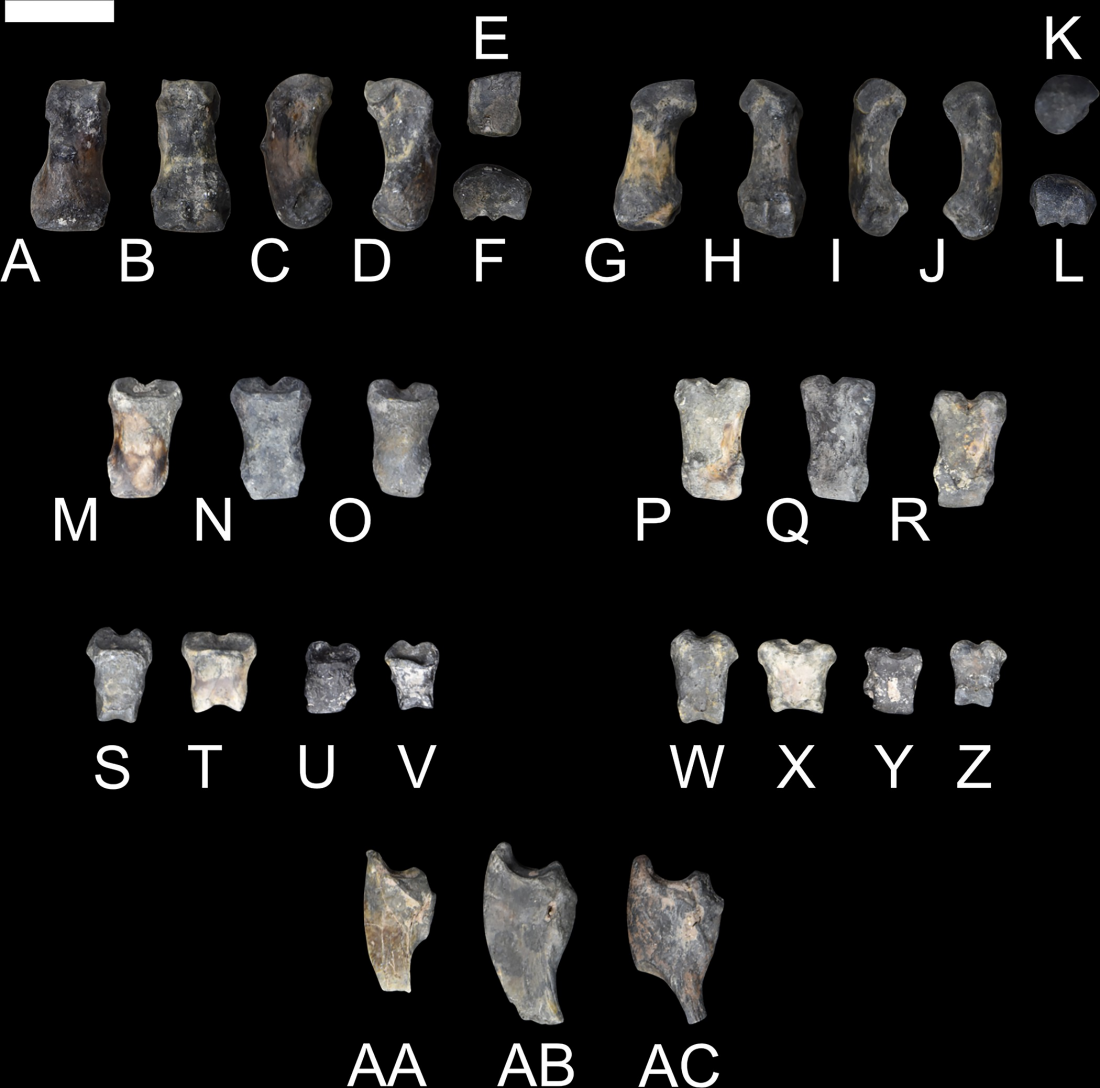
Part of the manus of *Conoryctes comma* (NMMNH P-79457). Metacarpal II (A–F) in dorsal (A), ventral (B), medial (C), lateral (D), proximal (E), and distal (F) views; metacarpal IV (G–L) in dorsal (G), ventral (H), medial (I), lateral (J), proximal (K), and distal (L) views; phalanges in dorsal (M–O, S–V) and plantar (P–R, W–Z) views; and unguals in medial view (AA–AC). Scale bar is 1cm.

**Fig 15:**
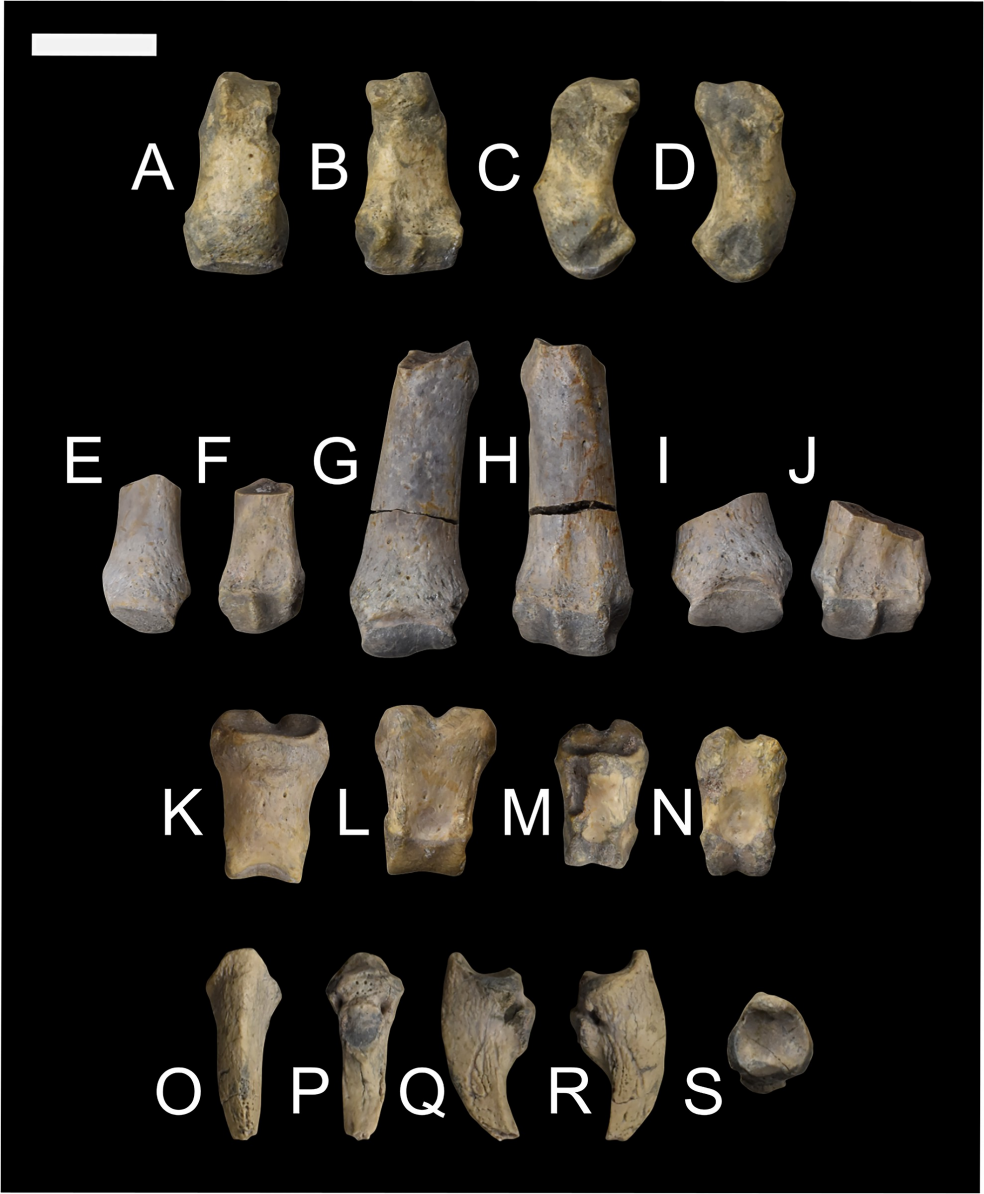
Part of the manus of *Conoryctes comma* (NMMNH P-48052). Metacarpal II (A–D) in dorsal (A), ventral (B), medial (C), and lateral (D) views; phalanges (E–N) in dorsal (E, G, I, K, M) and ventral (F, H, J, L, N) views; and ungual (O–S) in dorsal (O), ventral (P), medial (Q), lateral (R), proximal (S) views. Scale bar is 1cm.

The new specimens of *Conoryctes* illustrate part of the anatomy of the manus. NMMMNH P-79457 has two metacarpals (Mc II, Mc IV), seven phalanges, and three distal unguals (Fig 14), and NMMNH P-48052 has one complete metacarpal (Mc II), two complete phalanges, one complete distal phalanx, and an ungual (Fig 15).

The proximal end of the Mc II has a distinctive mediolaterally concave and dorsoventrally convex articular process, giving it a saddle-like shape (NMMMNH P-79457, NMMNH P-48052). This articular process is for the trapezoid and trapezium. The Mc IV is also short and stout (S7 Table) with a convex articular surface proximally for articulation with the unciform. Posteriorly both metacarpals are robust and mediolaterally-broad leading to a well-defined distal articulation for the phalanges. In dorsal aspect, there are extensor tubercles in the metacarpals of *Conoryctes*. As seen in Fig 14F and 14L, the distal end of the metacarpals is flat plantarly and convex dorsally. Distally the metacarpals have a dorsoventral ridge at the centre (Fig 15F, 15H, 15J). This ridge, or spine [50], forms a strong connection of the metacarpal with the first phalanx, which also has a small groove on the proximal end.

The phalanges are also relatively dorsoventrally flat and mediolaterally broad with prominent articular surfaces (NMMMNH P-79457, NMMNH P-48052, Figs 14 and 15). The terminal phalanges proximally have a deep curvature for the articulation with the distal phalanges. Dorsally the unguals have a “bony stop” [49] that prevented the dislocation with the phalanges, and plantarly there are large flexor tubercles preventing dislocations during flexion. Distally, the unguals are laterally compressed and well-curved. Functionally, the flat phalanges, the spine of the metacarpals providing internal stabilization between metacarpals and proximal phalanges, and the recurved unguals with the large flexor tubercle, show that *Conoryctes* was a plantigrade animal with digging adaptations, as we discuss in more detail below.

Comparing NMMNH P-79457 of *Conoryctes* to the right manus of *Onychodectes* (AMNH 16528) reveals similarities; in both, the metacarpals are long, wide proximally and distally and slender in the middle [6]. The metacarpals of *Conoryctes* are more robust than in *Onychodectes*, but similar to those of *Wortmania* (NMMNH P-19460) [3]. The phalanges of *Conoryctes* are longer and the unguals are shorter when compared to those of *Psittacotherium* (AMNH 2453) [6] and *Stylinodon* (FMNH PM 3895) [7].

A preserved ungual of *Escavadodon* (NMMNH P-22051) [34] is not complete but is curved in lateral view. The metacarpal has a similar flexor tubercle as seen in *Conoryctes* but is less prominent. The manus of *Conoryctes* differs from that of *Periptychus* (AMNH 17075) and *Pantolambda* (AMNH 16663). The metacarpals are concave on the plantar aspect in *Pantolambda*, less so in *Conoryctes* and even less in *Periptychus* [16, 35], indicative of their different locomotion styles.

### Innominate

**Fig 16:**
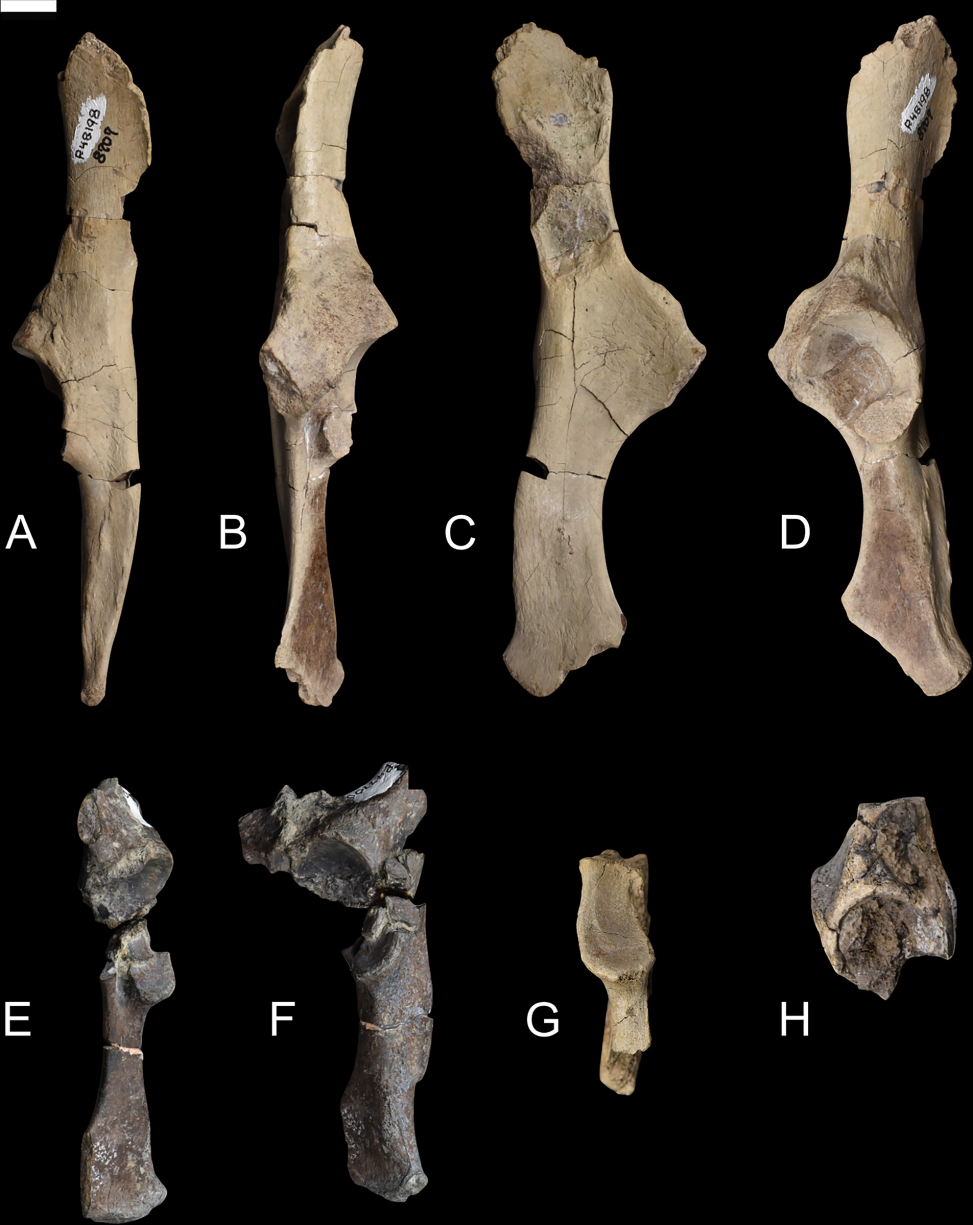
Innominate of *Conoryctes comma* [NMMNH P-48198 (A–D), NMMNH P-4700 (E–F), NMMNH P-61789 (G), NMMNH P-77896 (H)]. Views of NMMNH P-48198 are dorsal (A), ventral (B), medial (C) and lateral (D), NMMNH P-47700 in ventral (E) and lateral (F) views, and NMMNH P-61789 (G) and NMMNH P-77896 (H) in lateral view only. Scale bar is 1cm.

**Fig 17:**
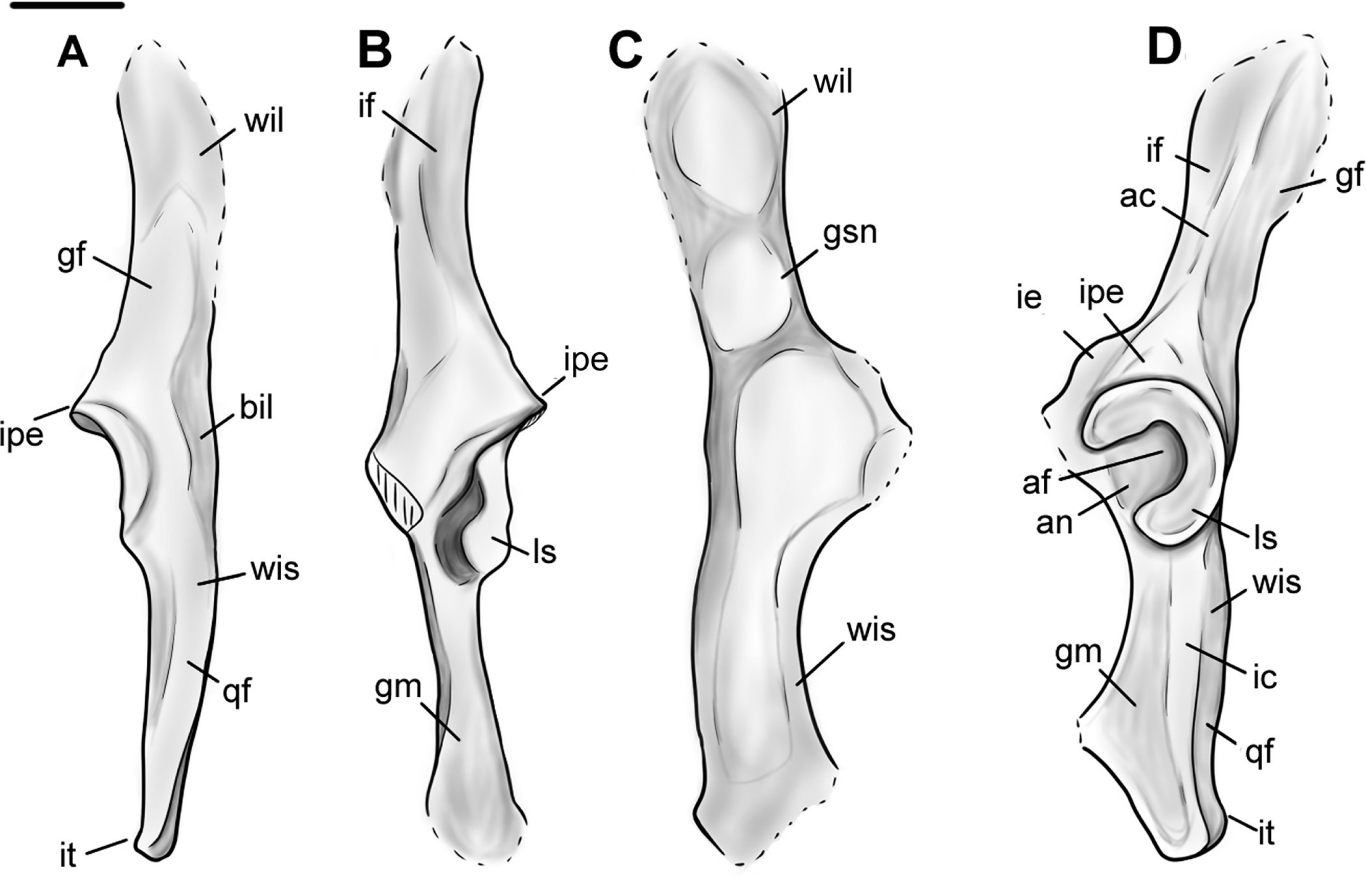
Drawing of the innominate of *Conoryctes comma*, based on NMMNH P-48198, in dorsal (A), ventral (B), medial (C) and lateral (D) views. ac: acetabular crest, af: acetabular fossa, an: acetabular notch, bil: body of the ilium, gf: gluteal fossa, gm: attachment for gemelli muscle, gsn: greater ischiatic notch, ic: ischiatic crest, ie: illiopubic eminence, if: iliacus fossa, ipe: iliopectineal eminence, it: ischiatic tuberosity, qf: quadratus femoris, ls: lunate surface, wil: wing of the ilium, wis: wing of the ischium. Scale bar is 1cm.

As seen in specimens NMMNH P-48198, NMMNH P-47700, NMMNH P-61789, and NMMNH P-77896 the innominate of *Conoryctes* is slender and elongate (Figs 16 and 17). The ilium was dorsoventrally-broad based on its preserved posterior end. In dorsal view, there is a well-defined gluteal fossa for the attachment of the gluteus maximus and gluteus medius muscles on the wing of the ilium [39]. The iliac neck is slender and expands more mediolaterally towards the body of the ilium. In ventral aspect, there is the posterior origin of a small and narrow iliac fossa. In lateral view, the iliac fossa and the gluteal fossa are separated by the acetabular crest. In *Conoryctes*, the acetabular crest is relatively sharp and prominent. Posterior to the acetabular crest is a protruding iliopectineal eminence. In medial view there are two attachment areas for the wings of the sacrum, forming the sacropelvic surface and the greater ischiatic notch.

The acetabulum, where the ilium, ischium and pubis meet and are fused in adults, is complete in most studied specimens (Fig 16). In dorsal aspect, the area of the acetabulum is convex and the rim of the acetabular fossa is parallel to the ischium and at approximately 45° with the ilium. In ventral view, there is the illiopubic eminence starting at the anterior border of the acetabulum. The illiopubic eminence, which provided attachment for the psoas minor muscle [39] of *Conoryctes*, is well-defined yet does not protrude far ventromedially. In lateral aspect, the rim of the acetabulum is continuous for at least three-fourths of the perimeter; it starts from the ischium and ends with a posterior opening, where the obturator foramen begins anteriorly. In *Conoryctes*, the lunate surface is continuous and only broader ventromedially, whereas the acetabulum extends more laterally from the ilium. The acetabular fossa is deep and roughly circular. Posteriorly towards the ischium, the acetabular fossa opens, forming the acetabular notch, which borders with the obturator foramen. The transverse acetabular ligament would have bridged this opening created by the acetabular notch. The lunate surface overhangs the acetabular fossa, creating a foramen, possibly for the obturator artery or nerves. In medial view, there is a well-defined large fossa with a very robust rim towards the dorsal aspect of the innominate (Fig 16). This fossa was the attachment surface for the coccygeus muscle, which would have expanded to the lateral end of the tail of *Conoryctes*.

The ischium is shorter than the ilium and more robust dorsoventrally than mediolaterally. In dorsal view, the ramus of the ischium becomes slender posteriorly. The ischiatic spine is more prominent anteriorly and medially transverse. In lateral aspect, the ischium has a well-defined crest (Fig 17D). On the dorsal side of this crest is a narrow, anteroposteriorly elongate fossa, for the attachment of the quadratus femoris muscle [39]. Ventral to this crest is a flat, broad, posteromedially-inclined area. In the posterior part of this area is a shallow broad fossa, where the quadratus femoris muscle originated. This muscle was responsible for the extension and lateral rotation of the joint between the femoral head and the acetabulum of the pelvis. In medial view, the ischiatic spine is more prominent anteriorly, and is not adjacent to the ischiatic tuberosity, which is dorsally-inclined (Fig 16C). Near the obturator foramen, the ischium of *Conoryctes* forms a continuous semicircular ridge.

The pubis is not complete in any studied specimen; however, based on NMMNH P-48198, the pubis probably extends from the acetabulum with an almost 70° angle from the ischium-ilium plane. The symphysis of the pelvis is not preserved, so the pubis-ischium attachment remains unknown for *Conoryctes*.

The innominate is known in some other taeniodonts including: *Onychodectes* (AMNH 3405) and *Stylinodon* (USNM 16664) [6, 7, 13]. *Conoryctes* has a slender ilium like *Onychodectes* and a well-defined gluteal fossa. The illiopubic eminence is more posteriorly placed in *Conoryctes* than *Onychodectes*. The acetabulum is more elongate anteroposteriorly in *Onychodectes*, less in *Conoryctes*, whereas it is circular in *Stylinodon*. In all three taxa though, the anterior border of the acetabulum that extends from the ilium protrudes more laterally. *Conoryctes* has a continuous lunate surface that is subequal in width as seen in *Stylinodon*. There is a foramen in the pubic area of the acetabular fossa in *Stylinodon* where the ligamentum teres attached; *Conoryctes* lacks this foramen. The ischium of *Stylinodon* expands more posteriorly, like in *Conoryctes*.

Comparing the innominate of *Conoryctes* to *Escavadodon*, *Prodiacodon* and *Leptictis* [1, 30, 34] reveals a few differences. The acetabular crest on the ilium in *Conoryctes* is similar to *Escavadodon*, and sharper than in leptictids. The illiopubic eminence of *Conoryctes* is similar to leptictids and not as prominent as in palaeanodonts. The iliopectineal eminence is placed less anteriorly in *Conoryctes* than in *Escavadodon*. The shape of the acetabulum and the ischium of both *Conoryctes* and *Escavadodon* are similar; however, the obturator foramen of *Escavadodon* is more elongate and oblique near the pubis-ilium connection.

The innominate of *Conoryctes* is distinct from those of *Periptychus* (NMMNH P-47693) [33] and *Pantolambda* (AMNH 16663) [16, 35, 49]. In dorsal view, the innominate of *Conoryctes* is less concave medially than *Periptychus*. Both *Conoryctes* and *Pantolambda* have an acetabular crest that is relatively sharp and prominent, unlike the more rounded one in *Periptychus*. The illiopubic eminence is more anteriorly placed in *Periptychus* than in *Conoryctes*. The ilium and ischium in *Conoryctes* and *Pantolambda* are approximately on the same plane, whereas they are angled in *Periptychus*.

### Femur

**Fig 18:**
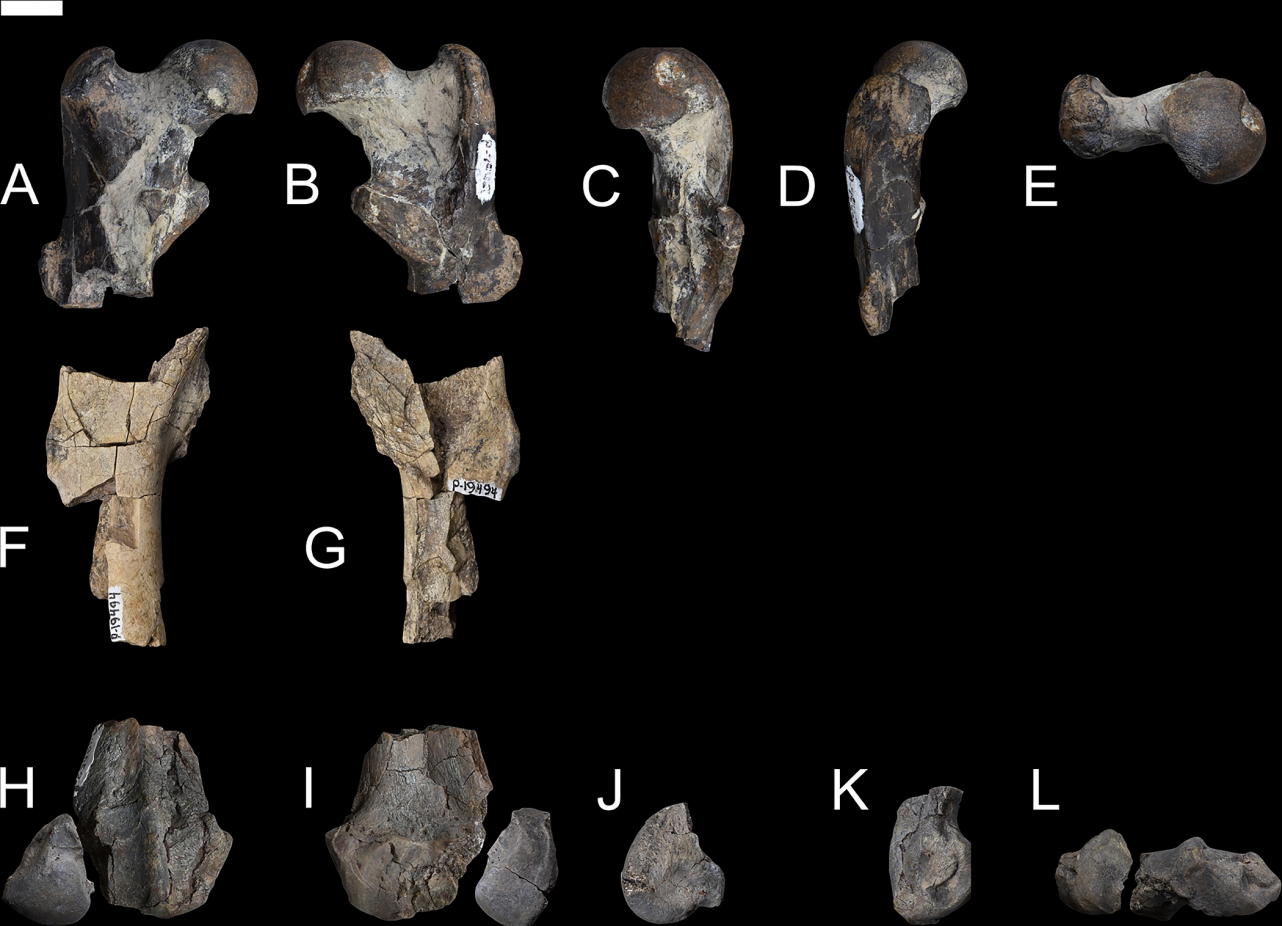
Femur of *Conoryctes comma* [NMMNH P-79457 (A–E), NMMNH P-19494 (F, G) and NMMNH P-48052 (H–L)] in anterior (A, F, H), posterior (B, G, I), medial (C, J), lateral (D, K), anterior (E) and posterior (L) views. Scale bar is 1cm.

**Fig 19:**
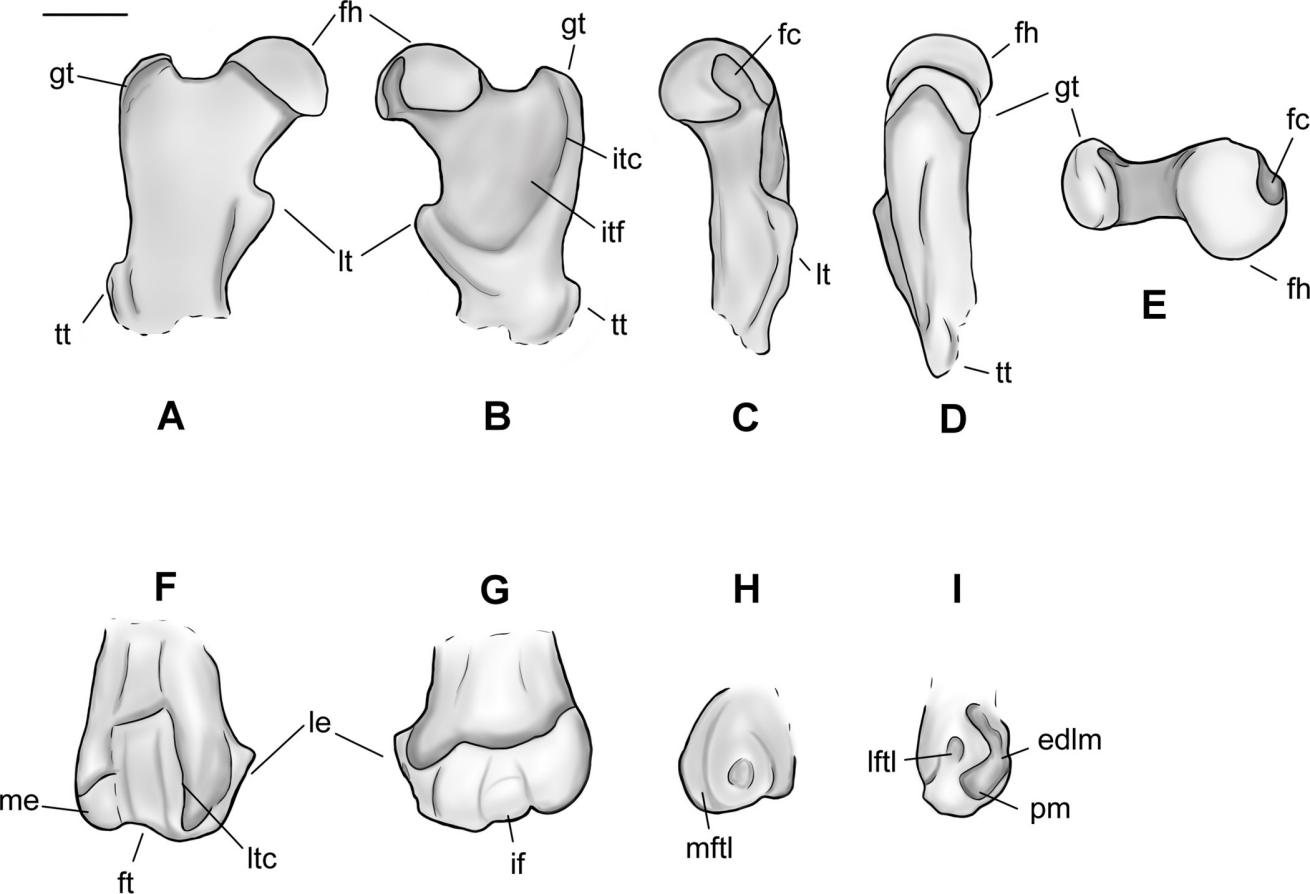
Drawing of the right femur of *Conoryctes comma*, based on specimens NMMNH P-79457 and NMMNH P-48052, in anterior (A, F), posterior (B, G), medial (C, H), lateral (D, I) and proximal (E) views. edlm: fossa for attachment of extensor digitorum longus muscle, fc: fovea capitis, fh: femoral head, ft: femoral trochlea, gt: greater trochanter, if: intercondylar fossa, itc: intertrochanteric crest, itf: trochanteric fossa, le: lateral epicondyle, lftl: fossa for attachment of lateral femorotibial ligament, lt: lesser trochanter, ltc: lateral trochlear crest, me: medial epicondyle, mftl: fossa for attachment of the medial femorotibial ligament, pm: fossa for attachment of popliteus muscle, tt: third trochanter. Scale bar is 1cm.

Based on several new specimens, including NMMNH P-79457, NMMNH P-19494 and NMMNH P-48052, the femur of *Conoryctes* is relatively robust and has well-developed areas for muscle attachments (Figs 18 and 19, S9 Table). The proximal epiphysis is broad mediolaterally, with a well-distinguished femoral head and a shorter greater trochanter (Fig 18A–18E). The femoral head is robust, hemispherical and extends medially. In anterior aspect, the articular surface of the femoral head is highly convex. The articular surface continues laterally, connecting smoothly to the femoral neck, whereas medially there is a rim formed between the articular surface of the femoral head and the femoral neck. In medial view, there is a deep fovea capitis starting almost from the top of the femoral head and continuing distally, interrupting the posteromedial articular surface of the femoral head (Fig 18C). The femoral neck is more slender than the femoral head, forming an angle with the proximodistal long axis of the femur. The greater trochanter is well separated from the femoral head and forms a rim with the intertrochanteric fossa in posterior view. The head of the greater trochanter is anterolaterally-inclined, and the apex of the greater trochanter has no marks for the attachment of the gluteus medius muscle [39]. In lateral aspect, there are two well-defined surfaces for the attachment of the gluteus profundus and the pyriformis muscles [39]. The intertrochanteric fossa of *Conoryctes* is deeply excavated and forms a continuum with the femoral head medially and a well-developed intertrochanteric crest laterally (NMMNH P-79457, NMMNH P-19494, Fig 18B). The distal end of the intertrochanteric fossa is well-defined and the internal and external obturators and the superior and inferior gemelli muscles were attached. The intertrochanteric fossa does not reach distally the same level as the third trochanter.

On the proximal diaphysis of the femur, the lesser trochanter extends medially forming an almost triangular flange for the attachment of the iliopsoas muscle [39]. In posterior view, the lesser trochanter makes contact with the intertrochanteric fossa, forming a large pectineal line for the attachment of the pectineus muscle (NMMNH P-19494) [39]. In *Conoryctes* the third trochanter is semi-circular, shorter proximodistally than the lesser trochanter and curved anteriorly. In anterior view, the third trochanter extends more laterally from the femoral shaft than the greater trochanter. On the distal diaphysis the femoral trochlea is shallow, mediolaterally broad and does not extend proximal to the diaphysis. The femoral trochlea terminates proximally at the same level as the proximal end of the femoral condyles.

On the distal epiphysis of *Conoryctes* the medial condyle is larger than the lateral condyle and probably extended more posteriorly (NMMNH P-48052, Figs 18 and 19). The femoral medial condyle has a large shallow fossa, in medial aspect, for the medial collateral ligament attachment (Fig 19H). The lateral epicondyle has three fossae in lateral aspect (Fig 19I). The largest fossa is shallow and anteroproximally-positioned for the attachment of the lateral collateral ligament [39]. Posterodistally, there is a large, deep and elongate fossa where the popliteus muscle originated. Anterior to this fossa, there is a small fossa, for the insertion of the extensor digitorum longus muscle. In posterior view, both medial and the lateral femoral condyles are smooth and rounded. The two condyles are divided by a deep intercondyloid fossa for the attachment of the cruciate ligament (Fig 19) [39].

The femur of *Conoryctes* shares resemblances with other genera of Taeniodonta. *Conoryctes*, as well as *Onychodectes* (AMNH 3405) [6, 13] and *Wortmania* (AMNH 3394) [3, 6, 15], have slender femora, with robust lesser and third trochanters protruding from the shaft. In anterior aspect, the femoral head of *Conoryctes* extends farther proximally than the greater trochanter. Whereas the femoral head is more extended in *Wortmania*. The femoral neck of *Conoryctes* is less robust than in *Wortmania*. The shape and robustness of the lesser and third trochanters are similar between *Conoryctes* and *Wortmania*. In both genera the lesser trochanter is more triangular, protruding more from the shaft than the more semi-circular third trochanter. In *Conoryctes*, *Onychodectes* and *Wortmania*, the lesser trochanter ends distally, at the proximodistal middle of the third trochanter, and the third trochanter has a flat apex. In posterior aspect, the intertrochanteric fossa of *Onychodectes* is shallower than in *Conoryctes* and *Wortmania*. The greater trochanter and the intertrochanteric fossa form a deep ridge in *Conoryctes* and *Wortmania*. Distally the femur of *Conoryctes*, *Onychodectes* and *Wortmania* are slender.

The femur of *Conoryctes* has some differences with other taeniodonts such as *Psittacotherium* (TMM 41364–1, AMNH 16560, NMMNH P-19713), *Ectoganus* (USNM 175531) and *Stylinodon* (cf. *Stylinodon mirus* USNM 18425, UW 2270) [6, 7]. The femoral neck of *Conoryctes* is less robust compared to *Psittacotherium* and *Stylinodon*. The femoral head extends farther proximally than the greater trochanter in *Conoryctes*, and even more so in *Psittacotherium* and *Stylinodon*, making that an anatomical feature shared in all studied taeniodonts. The lesser trochanter in *Conoryctes* is prominent, extending medially from the shaft, and is triangular, similar to *Ectoganus*. On the contrary, the lesser trochanter of *Psittacotherium* and *Stylinodon* is less prominent and more proximally-placed. The intertrochanteric fossa of *Psittacotherium* and *Ectoganus* is relatively shallow compared to *Conoryctes*. In *Stylinodon* (UW 2270), the intertrochanteric fossa is deep and there is a well-defined ridge with the greater trochanter; however, unlike *Conoryctes*, the fossa does not extend far distally. The third trochanter is more distally-positioned in *Onychodectes*, *Conoryctes* and *Wortmania*, than in *Psittacotherium*, *Ectoganus* and *Stylinodon*. On the distal epiphysis, the femoral trochlea ends at the same level as the most posterior parts of the femoral condyles, a characteristic seen in *Conoryctes*, as well as in *Psittacotherium* (TMM 41364–1), *Ectoganus* and *Stylinodon*. The femoral fossa is shallower in *Conoryctes* and *Ectoganus*, whereas it is deeper in *Psittacotherium* and *Stylinodon*. The lateral and medial fossae for the attachment of the collateral ligaments are well excavated in *Conoryctes*, as well as in *Psittacotherium* and *Ectoganus*.

The femur of *Conoryctes* shares only a few similarities with *Escavadodon*, *Prodiacodon* and *Leptictis* [1, 30, 34]. The femoral head of *Escavadodon* has a “mushroom” shape as described by Rose and Lucas [34] which could also apply to the femoral head of *Conoryctes*. *Conoryctes* and palaeanodonts have a large fovea capitis with a posteromedial groove, whereas *Escavadodon* and leptictids have a small fovea capitis. The greater trochanter projects less proximally in *Conoryctes*, whereas in *Prodiacodon* the femoral head and greater trochanter are almost on the same level, with the later extending more proximally. The lesser trochanter, as seen in *Escavadodon*, leptictids and palaeanodonts, extends posteromedially, less so than in *Conoryctes*. The third trochanter of *Conoryctes* has a crest-like shape, similar to *Escavadodon*. However, the third trochanter protrudes more laterally in *Conoryctes* and palaeanodonts compared to *Escavadodon*. On the distal epiphysis, the femoral trochlea of *Conoryctes*, and potentially all taeniodonts, does not extend proximally more than the proximal end of the distal condyles, unlike in *Prodiacodon* (UM 88105).

*Conoryctes*, *Periptychus* (NMMNH P-47693) [35] and *Pantolambda* (AMNH 16663) [16, 35, 49] have a robust femoral head with a deep fovea capitis. However, only in *Conoryctes* and *Periptychus* does the fovea capitis have a groove that excavates the articular surface of the femoral head posteromedially. In *Conoryctes* the greater trochanter does not extend more proximally than the femoral head, unlike in *Periptychus* and *Pantolambda*. In posterior view, the intertrochanteric fossa is deep in all three genera, with the intertrochanteric crest being more prominent in *Conoryctes* than in *Periptychus* and *Pantolambda*. The lesser trochanter is triangular, protruding posteromedially in *Conoryctes* and *Periptychus*. The third trochanter of *Conoryctes* and *Pantolambda* is less prominent than in *Periptychus*. In *Conoryctes* the third trochanter is more proximal and positioned near the lesser trochanter, whereas in *Periptychus* and *Pantolambda* the third trochanter is more distally placed on the femoral shaft. Distally, the femoral trochlea of *Conoryctes* and *Pantolambda* end approximately at the most proximal end of the condyles. However, in *Periptychus* the femoral trochlea continues more proximally than the femoral condyles. In lateral aspect, the lateral epicondyle of *Conoryctes* has three well-defined fossae for muscle attachments, as also seen in *Periptychus*.

### Patella

**Fig 20:**
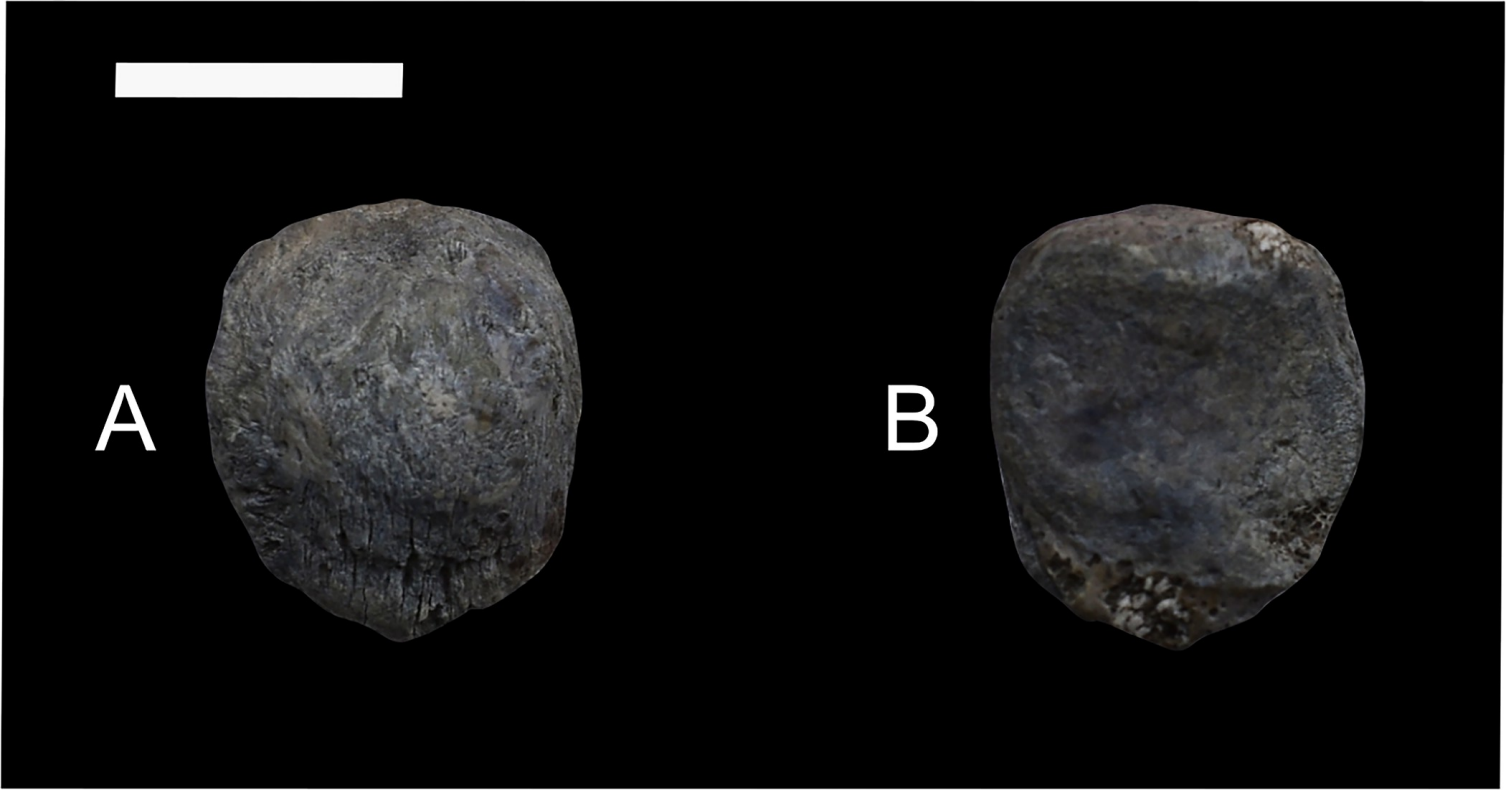
Patella of *Conoryctes comma* (NMMNH P-48052) in anterior (A) and posterior (B) views. Scale bar is 1cm.

*Conoryctes* has an ossified patella (NMMNH P-48052, (Fig 20). In anterior aspect, the patella is almost circular in profile, with a small distal apex for attachment of the patellar ligament [39]. Proximally, the base of the patella extends from the medial to lateral side, for insertion of the quadriceps tendon. Distally, there are proximodistally-oriented striations where the patellar tendon originated, connecting the patella with the tibia. In posterior view, there are two facets, one medially and the other laterally placed, divided by a weak ridge that is proximodistally-oriented.

The patella of *Conoryctes* is similar to that of *Onychodectes* (AMNH 3576a) and *Stylinodon* (USNM 16664), as they are almost circular in profile and have two facets in posterior view [6, 7]. The patella of *Conoryctes* is different than that of *Periptychus* since the latter has a more well-formed distal apex and a pyriform patella [35].

### Tibia

**Fig 21:**
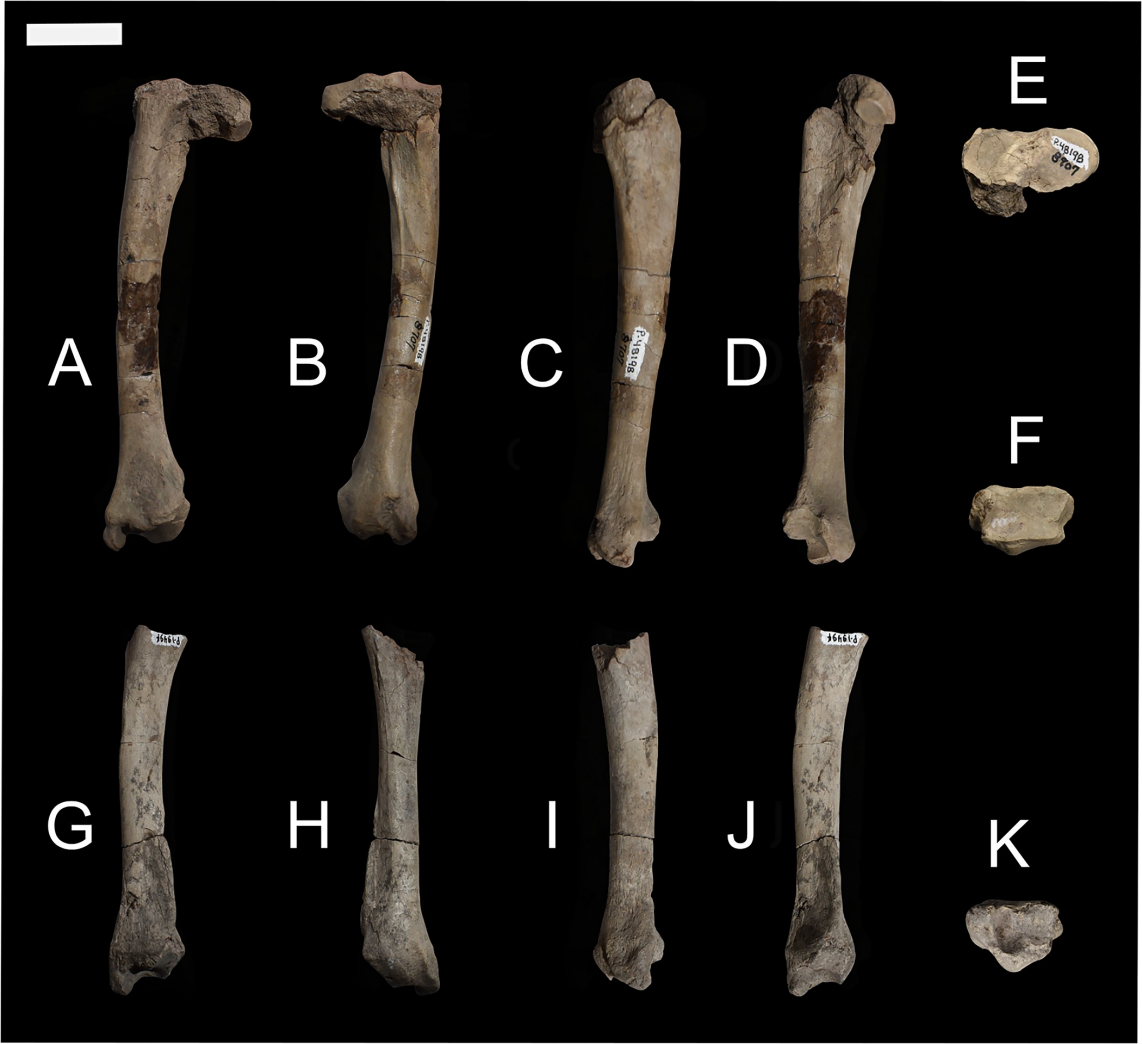
Tibiae of *Conoryctes comma* [NMMNH P-48198 (A–F) and NMMNH P-19494 (G–K)] in anterior (A, G), posterior (B, H), medial (C, I), lateral (D, J), proximal (E) and distal (F, K) views. Scale bar is 1cm.

**Fig 22:**
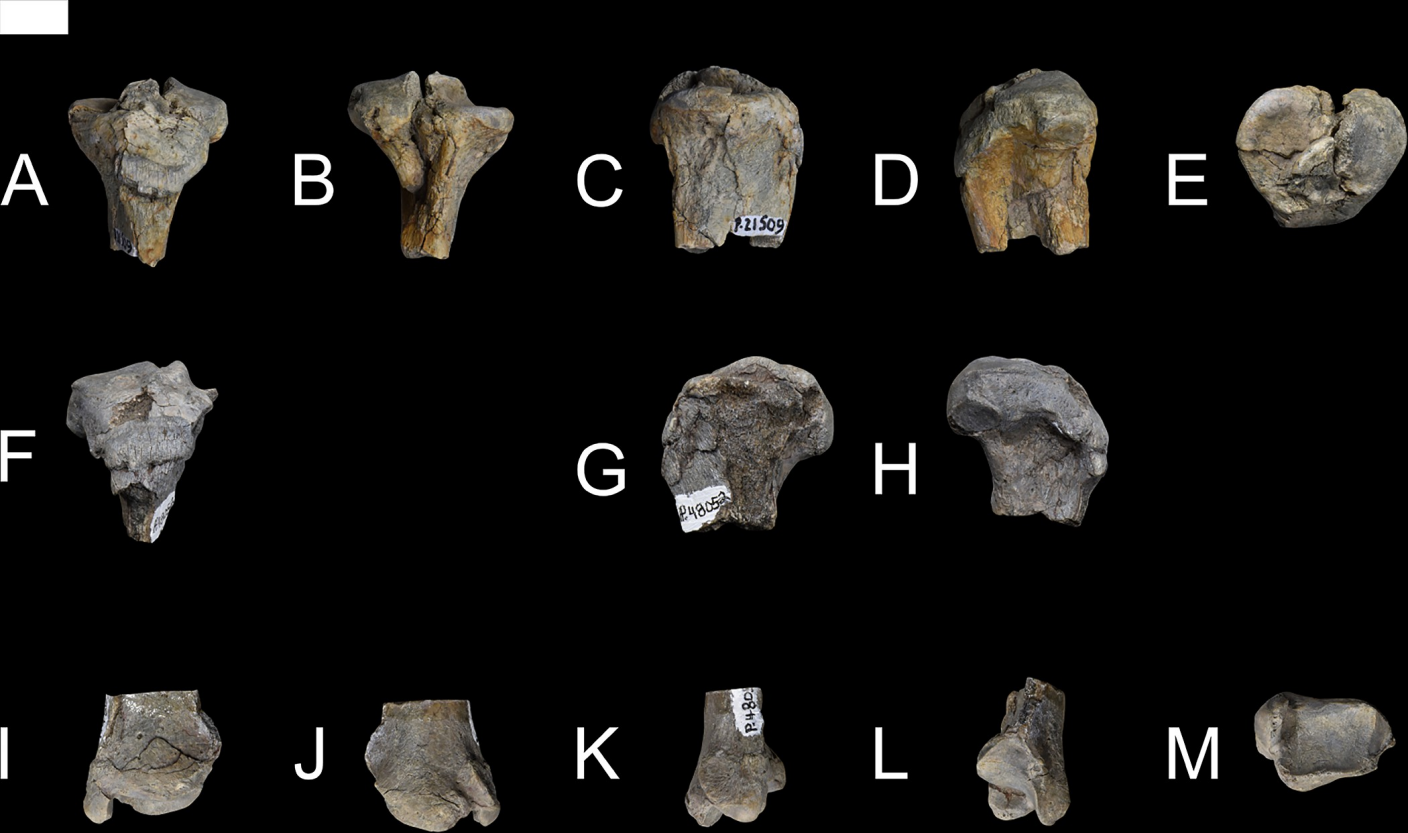
Tibiae of *Conoryctes comma* [NMMNH P-21509 (A–E) and NMMNH P-48052 (F–M)] in anterior (A, F, I), posterior (B, J), medial (C, G, K), lateral (D, H, L), proximal (E) and distal (M) views. Scale bar is 1cm.

**Fig 23:**
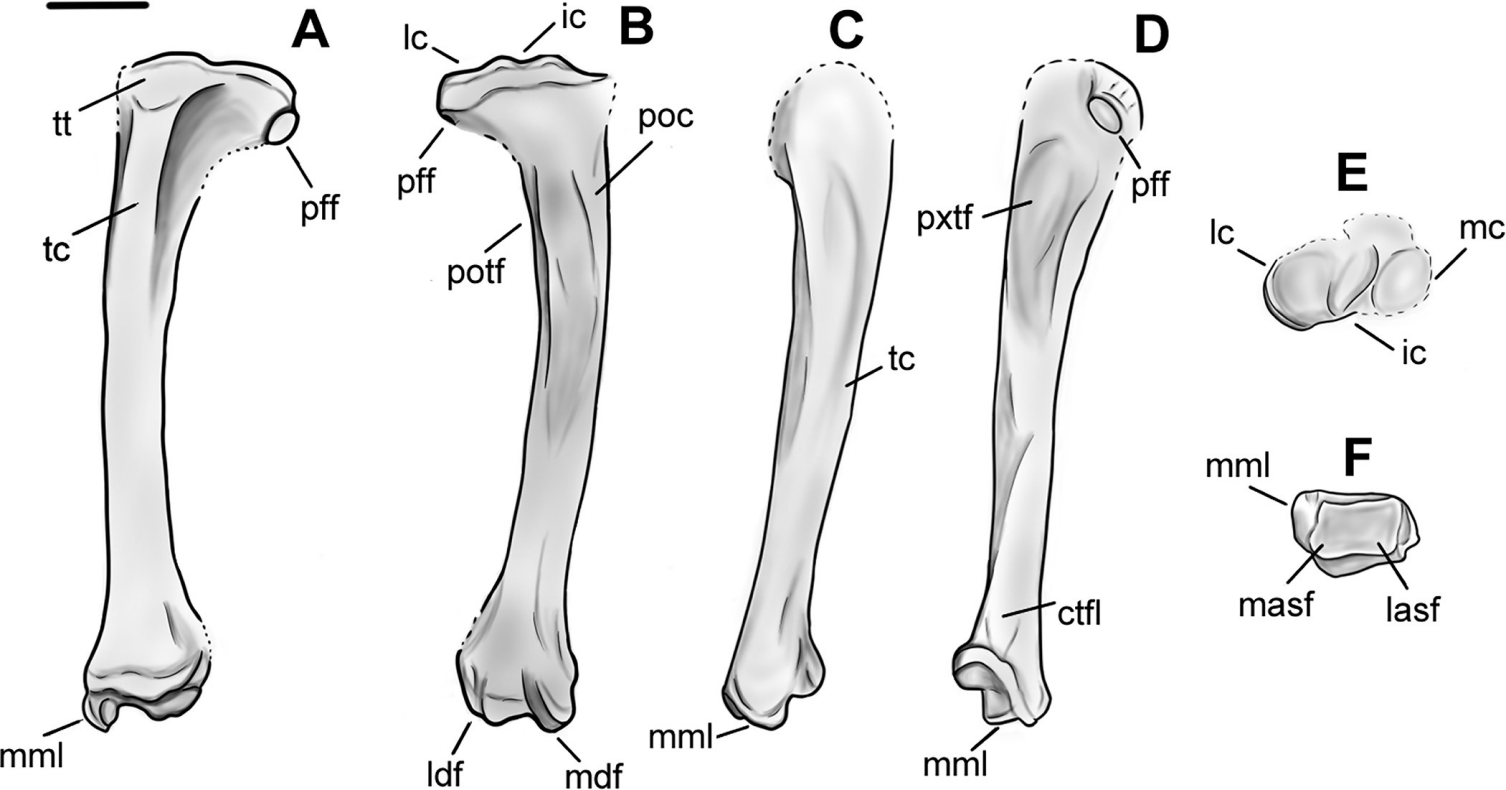
Drawing of the left tibia of *Conoryctes comma*, based on NMMNH P-48198, in anterior (A), posterior (B), medial (C) lateral (D), proximal (E) and distal (F) views. ctfl: crest of the tibiofibular ligament, ic: intercondyloid eminence, lasf: lateral astragalar facet, lc: lateral condyle, ldf: lateral digital flexor, masf: medial astragalar facet, mc: medial condyle, mdf: medial digital flexor, mml: medial malleolus, pff: proximal fibular facet, poc: popliteus crest, potf: posterior tibial fossa, pxtf: proximal tibial fossa, tc: tibial crest, tt: tibial tuberosity. Scale bar is 1cm.

The tibia of *Conoryctes* is slender and extends mediolaterally more on the proximal end than the distal end (NMMNH P-48198, NMMNH P-19494, NMMNH P-21509, NMMNH P-48052, Figs 21–23, S11 Table). On the proximal epiphysis are the lateral and medial condyles for the tibia, which are anteroposteriorly subequal in depth and are divided by a mediolaterally-wide intercondylar area (NMMNH P-48198, NMMNH P-21509, NMMNH P-48052). In proximal view, the medial condyle is wider mediolaterally and more concave than the lateral condyle. The medial condyle is oval and anteroposteriorly elongate. Medially, the medial condyle is almost flat and there is a thick neck around the condyle for the medial collateral ligament. Laterally, the medial condyle rises more steeply and leads to the medial intercondylar eminence for attachment of the medial meniscus. In proximal view, the lateral condyle is circular and almost flat with a convex medial border leading to the lateral intercondylar eminence. On the lateral side is a lateral eminence for the attachment of the lateral meniscus. The lateral intercondylar eminence is flatter, less prominent and more posteriorly placed than the medial. Both the medial and lateral tibial condyles are roughly equally elevated proximally.

On the proximal diaphysis is a prominent tibial tuberosity which expands mediolaterally, providing attachment for the patellar tendon (Fig 22A and 22F). The tuberosity originates proximally at the level of the lateral and medial condyles, but does not contact them, and continues distally. The proximal fibular facet is flat and circular, and so the tibia and fibula were not fused proximally in *Conoryctes*.

The shaft of the tibia is slender and, in anterior view, is straight medially and concave laterally (NMMNH P-48198, NMMNH P-19494). The tibial crest originates proximally and continues distally, extending for almost 70% of the tibial shaft. The tibialis anterior muscle passed lateral to the tibial crest, following along the crest. In lateral view, the proximal tibial fossa is large and elongates distally, continuing down 30% of the tibial shaft. It forms part of the area where the tibialis cranialis muscle originated. In posterior aspect, the popliteal line extends from the proximal diaphysis to almost the middle of the shaft. The proximal diaphysis is broader mediolaterally than the distal diaphysis.

In anterior view, the distal diaphysis of *Conoryctes* has a groove for the crural extensor retinaculum ligament (Fig 21A and 21G) [39]. In posterior aspect, two noticeable grooves extend more distally to the epiphysis of the tibia (Fig 21B and 21H). The lateral groove is adjacent to the crest of the tibiofibular ligaments and is deep and narrow. The short lateral collateral ligaments passed through this groove, connecting the tibia with the proximal calcaneum, as well as the lateral digital flexor muscle [39]. The medial groove is deep and narrow for the medial digital flexor muscle. The medial collateral ligaments were attached to the flexor sulci, connecting the tibia with the distal calcaneum (Fig 21B). In lateral view, there is the small, distal, semi-circular fibular facet. In *Conoryctes* the tibia and fibula are unfused distally and thus form a synovial joint both proximally and distally.

The distal epiphysis is mediolaterally broad and strongly concave (NMMNH P-48198, NMMNH P-19494, NMMNH P-48052). A robust medial malleolus, with a deep flexor sulcus, seen in posterior view, extends distally (Figs 21B, 21H and 22J). In distal view, the articular surface with the astragalus consists of medial and lateral astragalar facets. These are continuous in *Conoryctes* and the medial astragalar facet is deeper than the lateral (Figs 21K and 22M). The posterior border of the distal epiphysis ends more distally than the anterior border. This was probably to prevent dislocation of the astragalus and the tibia in extreme dorsiflexion of the pes of *Conoryctes*.

There are many resemblances between the tibiae of *Conoryctes* and other taeniodonts, including *Onychodectes tisonensis* (AMNH 3405), *Wortmania otariidens* (AMNH 3394), *Psittacotherium multifragum* (TMM 41364–1, AMNH 15938, and NMMNH P-19713) and cf. *Stylinodon mirus* (USNM 18425) [3, 6, 7, 13]. Apart from *Onychodectes*, which is known only from the distal end of the tibia, in all other studied genera the posterior diaphysis of the tibia is more slender than the anterior diaphysis, with a less prominent tibial crest and no tuberosity. Another common feature is the prominent medial malleolus extending distally, even in more robust taxa like cf. *Stylinodon mirus* (USNM 18425). In all specimens of *Conoryctes*, the distal epiphysis extends distally more posteriorly than anteriorly. The presence of a well-distinguished flexor sulcus on the medial malleolus can be seen in the specimens of *Conoryctes* and *Wortmania*. Another common feature within Taeniodonta, based on the studied specimens, is the unfused tibia and fibula both proximally and distally. Moreover, even the smaller taxa have a well-defined crest of the tibiofibular ligament, which is even more prominent in larger taxa. However, there are a few differences between *Conoryctes* and other taeniodonts. *Conoryctes* has a shallower posterior tibial fossa than *Stylinodon*, *Wortmania* and *Psittacotherium*. On the medial side of the shaft, *Conoryctes* lacks a medial protuberance that is near the distal border of the posterior tibial fossa, a feature seen in *Wortmania* and more protruding in *Stylinodon*. The proximal fibular facet is well distinguished at the laterodistal end of the proximal epiphysis in *Conoryctes*. However, the facet is not as eminent and is placed more on the lateral aspect of the epiphysis than distally in *Wortmania*, *Psittacotherium* and *Stylinodon*.

The tibia of *Conoryctes* was also compared to *Escavadodon*, *Prodiacodon* and *Leptictis* [1, 30, 34]. The shaft in all these taxa is slender; however, *Conoryctes* has a straight shaft, compared to the curved and posteriorly concave shaft in *Escavadodon*, *Prodiacodon* and *Leptictis*. The tibial crest is large and flat in *Conoryctes*, whereas in *Escavadodon* and leptictids the crest is sharp, and in palaeanodonts it is more rounded. The tibia and fibula are unfused both proximally and distally in *Conoryctes*, whereas the two bones are fused distally in *Escavadodon* and *Prodiacodon*. In leptictids, the tibia and fibula are also fused distally and continue being fused for nearly half the length of the shaft. In *Conoryctes* the distal fibular facet is distally-restricted, whereas in *Escavadodon* and leptictids the articulation extends more proximally [34]. The medial malleolus is prominent in *Conoryctes* and leptictids, but less so in *Escavadodon* and palaeanodonts. All these four taxa have a well-developed flexor sulcus posteriorly [30, 34]. On the distal epiphysis, the medial and lateral astragalar facets in *Conoryctes* are subequal mediolaterally and anteroposteriorly and the medial facet is deeper. However, *Escavadodon* and leptictids have a deeper groove for the lateral astragalar rim.

Relative to *Periptychus* (NMMNH P-47693) [35], and especially *Pantolambda bathmodon* (AMNH 16663) [16, 35, 49], the tibia of *Conoryctes* is more slender and the shaft is less curved. *Conoryctes* has a smaller tibial crest and a less prominent tuberosity of the tibial crest compared to *Periptychus* and *Pantolambda*. In proximal view, the medial condyle is oval, wider mediolaterally and more concave than the lateral condyle, both in *Conoryctes* and *Periptychus*. Distally, *Conoryctes* and *Pantolambda* have a more prominent medial malleolus than *Periptychus*. The medial flexor sulcus is not noticeable in either *Periptychus* or *Pantolambda*. In all three genera, the tibia and fibula are unfused both posteriorly and distally. The distal fibular facet is very close to the lateral astragalar facet and laterally-oriented both in *Conoryctes* and *Periptychus*. In distal view, both *Conoryctes* and *Periptychus* have a deeper medial than lateral astragalar facet.

### Astragalus

**Fig 24:**
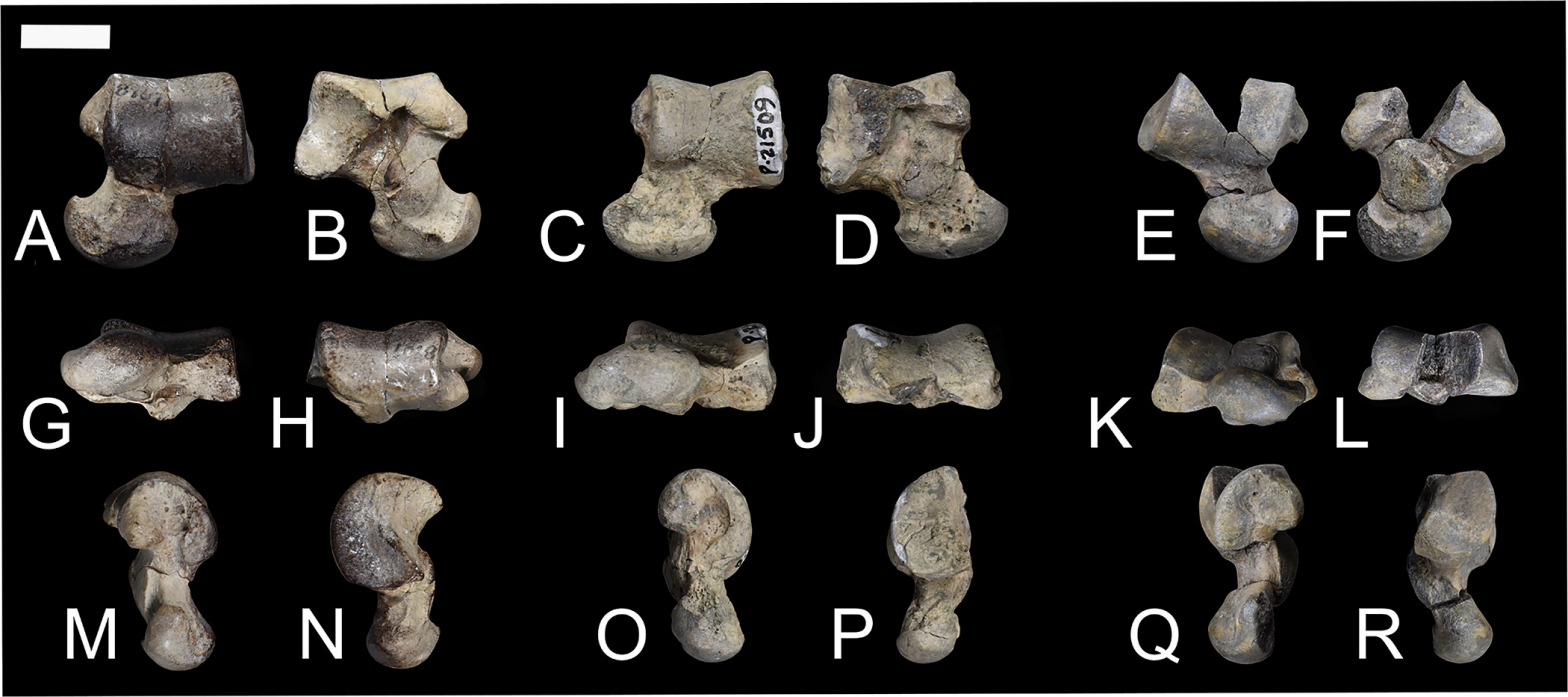
Astragali of *Conoryctes comma*, including NMMNH P-48198 (A, B, G, H, M, N), NMMNH P-21509 (C, D, I, J, O, P), NMMNH P-48052 (E, F, K, L, Q, R) in dorsal (A, C, E) and plantar (B, D, F), anterior (G, I, K) and posterior (H, J, L), and medial (M, O, Q) and lateral (N, P, R) views. Scale bar is 1cm.

**Fig 25:**
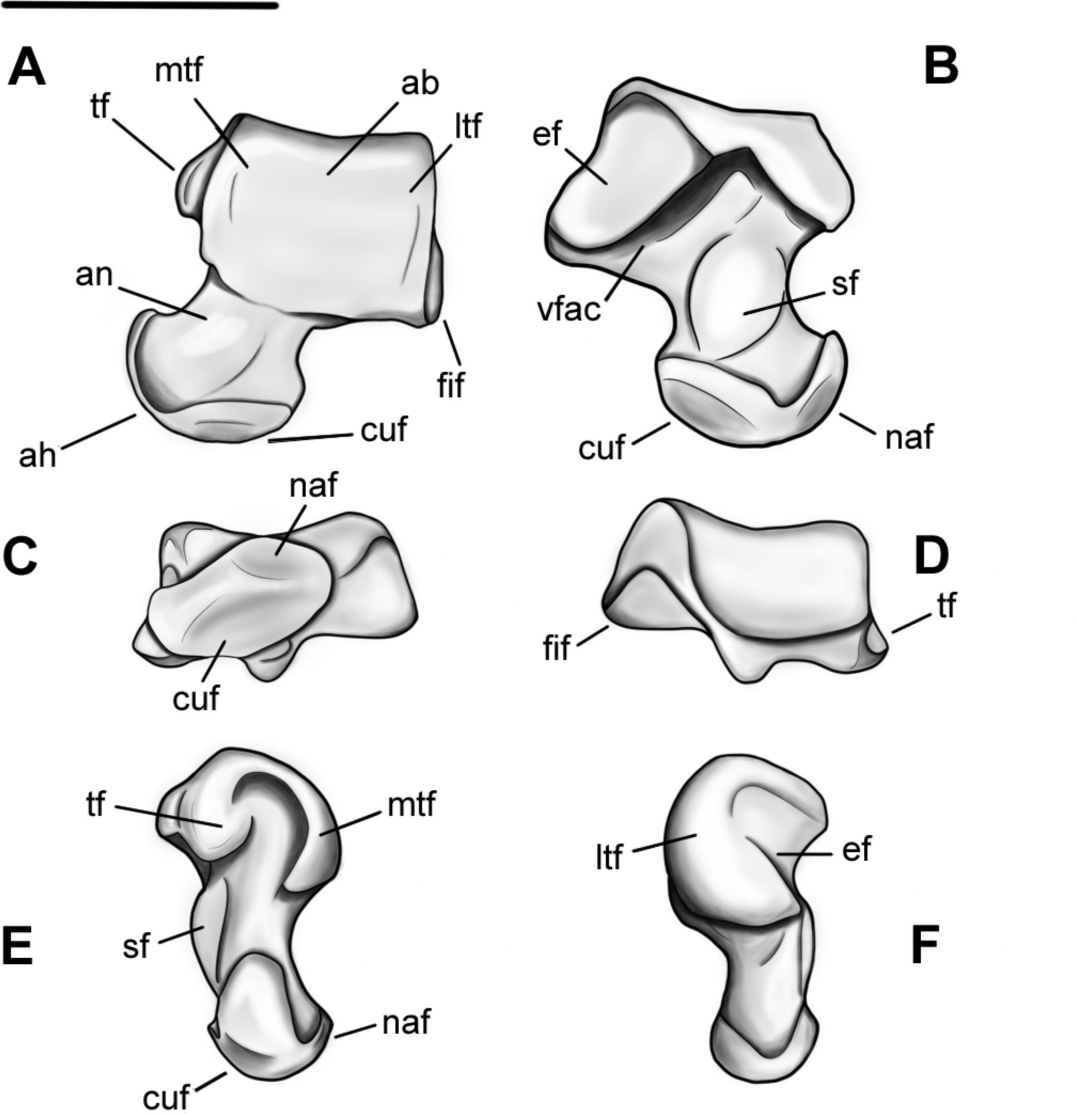
Drawing of the left astragalus of *Conoryctes comma* based on NMMNH P-48198 in dorsal (A), plantar (B), anterior (C), posterior (D), medial (E) and lateral (F) views. ab: astragalar body, ah: astragalar head, an: astragalar neck, cuf: cuboid facet, ef: ectal facet, fif: fibular facet, ltf: lateral tibial facet, mtf: medial tibial facet, naf: navicular facet, sf: sustentacular facet, tf: tibial facet, vfac: ventral foramen of the astragalar canal (sulcus astragali). Scale bar is 1cm.

In general, the astragalus of *Conoryctes* is anteroposteriorly elongate with a rectangular body, a moderately long and slender neck, and a broad head (NMMNH P-48052, NMMNH P-21509, NMMNH P-48198, Figs 24 and 25). The body of the astragalus, in dorsal view, is mediolaterally-wide and anteroposteriorly short. The trochlea is broad and moderately grooved (U-shaped) with subequal lateral and medial rims in anteroposterior length. The medial tibial facet is larger and more prominent than the lateral tibial facet. Distally, the trochlea does not extend to the astragalar neck, and there is no pit for the anterior process of the tibia. In posterior view, the lateral rim of the astragalar trochlea is less elevated than the medial rim of the medial tibial facet, providing *Conoryctes* with a degree of stability at the ankle joint during flexion and extension of the foot. The posterior border of the trochlea expands plantarly, allowing more movement of the tibia during flexion of the foot. *Conoryctes* lacks a dorsal foramen of the astragalar canal (Fig 24H and 24J). In plantar view, there is a broad and deep sulcus astragali where the tendons of the tibialis posterior muscle attached [39]. The ectal facet has a deep U-shape and is broader posteriorly with a narrower ending anteriorly. The fibular facet of the astragalus is almost flat and adjacent to the anterior border of the ectal facet. The tibial facet of the astragalus protrudes medially and interlocks well with the prominent medial malleolus of the distal tibia. In medial aspect, the tibial facet is separated from the medial rim of the trochlea by a deep groove.

The astragalar neck of *Conoryctes* is long (28% of the total anteroposterior length) and medially-inclined (76°) relative to the astragalar body. Anteriorly, the neck broadens mediolaterally as it reaches the astragalar head. In plantar view, there is a well-defined sustentacular facet, making the neck dorsoplantarly-broad. The sustentacular facet is oval, anteroposteriorly-elongate, and is almost in contact with the cuboid facet (Fig 24B and 24D). The sustentacular facet is relatively smaller than the ectal facet. Medially and laterally, there are grooves next to the sustentacular facet, where the deltoid and talofibular ligaments continued, respectively [39].

The astragalar head of *Conoryctes* is robust, almost equally broad mediolaterally as dorsoplantarly deep (S12 Table). The navicular facet is convex and comes smoothly in contact with the groove of the talofibular ligament laterally. In medial view, the navicular facet is narrow mediolaterally with a sharp medial point, and well-separated from the dorsal deltoid groove (Fig 24M, 24O and 24Q). In plantar view, the cuboid facet is in contact with the navicular facet anteriorly and close to the sustentacular facet posteriorly. In anterior aspect, the cuboid and navicular facets contact each other (Fig 24G, 24I and 24K).

The astragalus of *Conoryctes* is similar to *Onychodectes* (AMNH 3405, AMNH 3576a and AMNH 27679) and less robust than the astragali of *Psittacotherium* (NMMNH P-19713, TMM 41364–1) and cf. *Stylinodon mirus* (USNM 18425) [6, 7, 13]. Regardless of the size difference, there are many anatomical similarities, i.e., a U-shaped astragalar trochlea, with subequal astragalar trochlear rims, a deep plantar sulcus astragali, the lack of a dorsal foramen for the astragalar canal, and a very slender neck. The trochlea of the astragalar body is concave in *Conoryctes*, but less so in *Onychodectes*, *Psittacotherium* and *Stylinodon*. On the trochlea, the rims are mostly anteroposteriorly subequal in *Conoryctes*, unlike *Stylinodon* that has an anteroposteriorly longer and dorsoplantarly taller lateral trochlear rim. *Onychodectes* has a wider neck mediolaterally and is more laterally-inclined than in *Conoryctes*. The neck of *Psittacotherium* (TMM 41364–1, NMMNH P-19713) is short and the tibial facet extends more medially than in *Conoryctes*. In *Stylinodon* the neck is almost vertical to the mediolateral plane of the astragalar body. The astragalar head, for all studied taeniodonts, has facets for the cuboid and the navicular. The cuboid facet is posteriorly extended in *Conoryctes*, whereas it extends less so in *Onychodectes*. Unlike *Conoryctes*, *Onychodectes* has a more robust neck dorsoplantarly.

There are basic differences between the astragalus of *Conoryctes* and *Procerberus* (AMNH 117454) [51]. *Procerberus* has a flatter astragalar trochlea than *Conoryctes*. In posterior view, the trochlear rims of *Procerberus* are more laterally- inclined to the body of the astragalus. This feature is characteristic of *Procerberus* and is not seen in any of the taeniodonts studied. A dorsal foramen of the astragalar canal is not present in *Conoryctes*, and, as suggested by Szalay and Decker [51], *Procerberus* has no astragalar canal either. Unlike *Conoryctes*, the astragalar neck of *Procerberus* is wider mediolaterally and less medially-inclined. Compared to *Conoryctes*, *Procerberus* has a shallower sulcus astragali, and the sustentacular facet is more circular than elongate. On the head of the astragalus, the navicular facet of *Procerberus* is not as evident as in *Conoryctes*.

The astragalus of *Conoryctes* is similar to the astragali of *Escavadodon* (NMMNH P-22051), leptictids, and palaeanodonts [30, 34] as they are more anteroposteriorly elongate than mediolaterally wide. *Conoryctes*, *Escavadodon* and *Palaeanodon* have no evidence of a dorsal foramen on the astragalar body. *Conoryctes* has a more mediolaterally-broad astragalus compared to the more anteroposteriorly-elongate astragali of *Escavadodon* and other Leptictidae [30, 31, 34]. *Conoryctes* and *Escavadodon* have subequal trochlear rims in anteroposterior length and subequal elevation dorsoplantarly. In leptictids, however, the lateral trochlear rim is longer than the medial rim. *Conoryctes* and leptictids have protruding medial and fibular tibial facets, whereas only the fibular facet of *Escavadodon* is moderately noticeable. In *Conoryctes* and *Escavadodon* the neck is shorter than the mediolateral width of the medial trochlea. However, in leptictids the astragalar neck is longer than the mediolateral width of the medial trochlea. The length of the astragalar neck is less than 30% of the total astragalar length in *Conoryctes* and *Leptictis* but more than 40% in *Escavadodon*. The sustentacular facet is closer to the navicular and cuboid facets in *Conoryctes*, whereas these are more separated in *Escavadodon* and leptictids. The astragalar head of leptictids is less mediolaterally wide than in *Conoryctes*.

The astragalus of *Conoryctes* is more similar to that of *Periptychus* (NMMNH P-47693) [35] than to *Pantolambda* (AMNH 16663) [16, 35, 49]; *Pantolambda* has a unique and distinct astragalus. The astragalar head of *Conoryctes* and *Periptychus* are similar because the rims are subequal in size and parallel, whereas *Pantolambda* has oblique rims, with the lateral rim being longer anteroposteriorly. The medial tibial facet is more prominent in *Conoryctes* than in *Periptychus* and *Pantolambda*. In *Conoryctes* the fibular facet extends less laterally than in *Periptychus*. Both *Periptychus* and *Pantolambda* have deep sulcus astragali that lead to the dorsal foramen of the astragalar canal; this is missing from *Conoryctes*. In plantar aspect, the ectal facet is almost triangular in *Conoryctes* and *Pantolambda*, whereas it is more rectangular in *Periptychus*. The neck is more slender in *Conoryctes* than in *Periptychus*, and very different to the uniquely-shaped astragalar neck of *Pantolambda*. In plantar aspect, the sustentacular facet is anteroposteriorly-elongate in *Conoryctes* and almost circular in *Periptychus* and *Pantolambda*. The head of the astragalus in *Conoryctes* is convex, with facets for the cuboid and the navicular, with the latter expanding posteriorly in medial aspect, similar to that of *Periptychus*.

### Calcaneum

**Fig 26:**
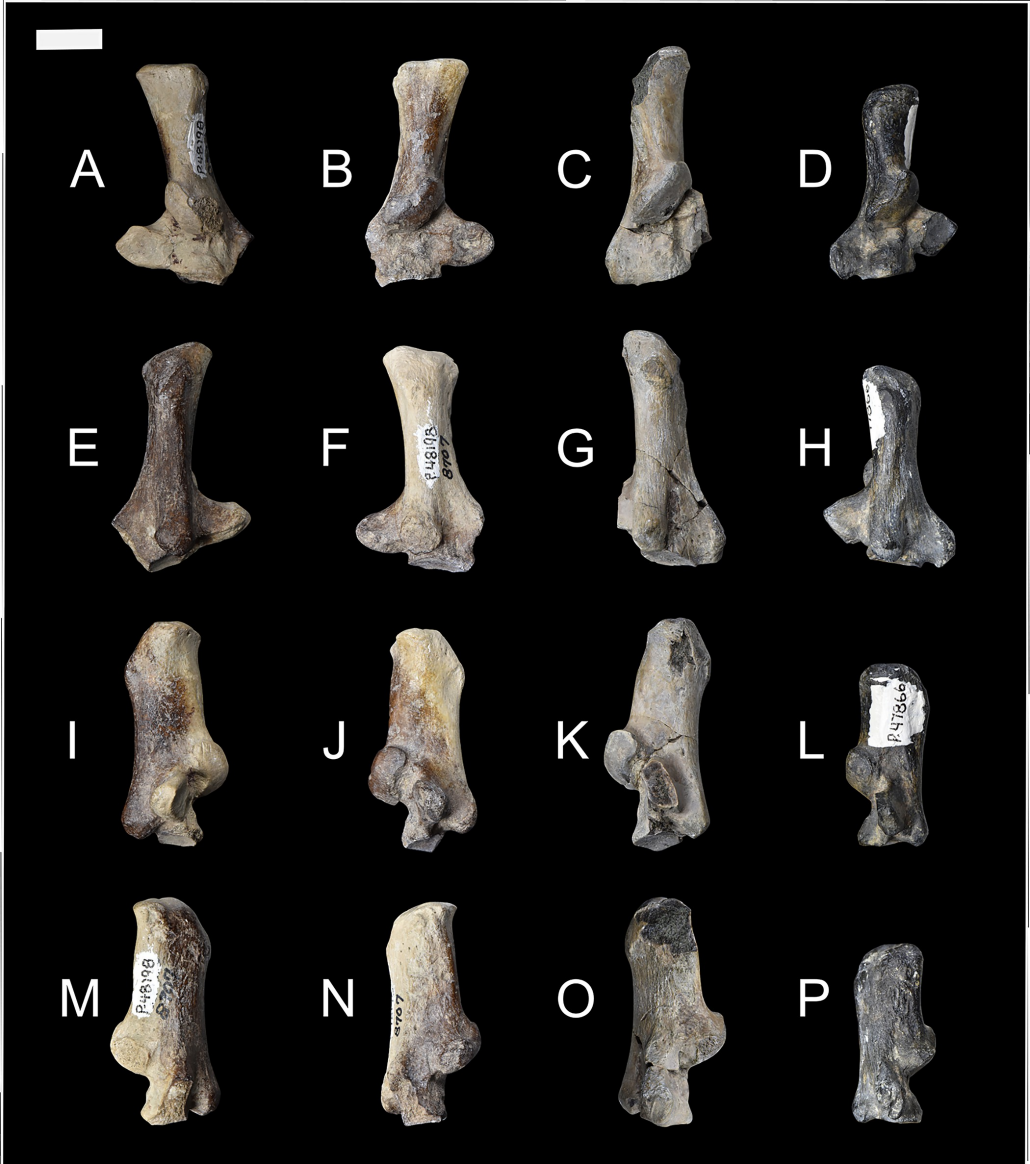
Calcanei of *Conoryctes comma* [NMMNH P-48198 (A, B, E, F, I, J, M, N), NMMNH P-48052 (C, G, K, O) and NMMNH P-47866 (D, H, L, P)] in dorsal (A–D), plantar (E–H), medial (I–L), and lateral (M–P) views. Scale bar is 1cm.

**Fig 27:**
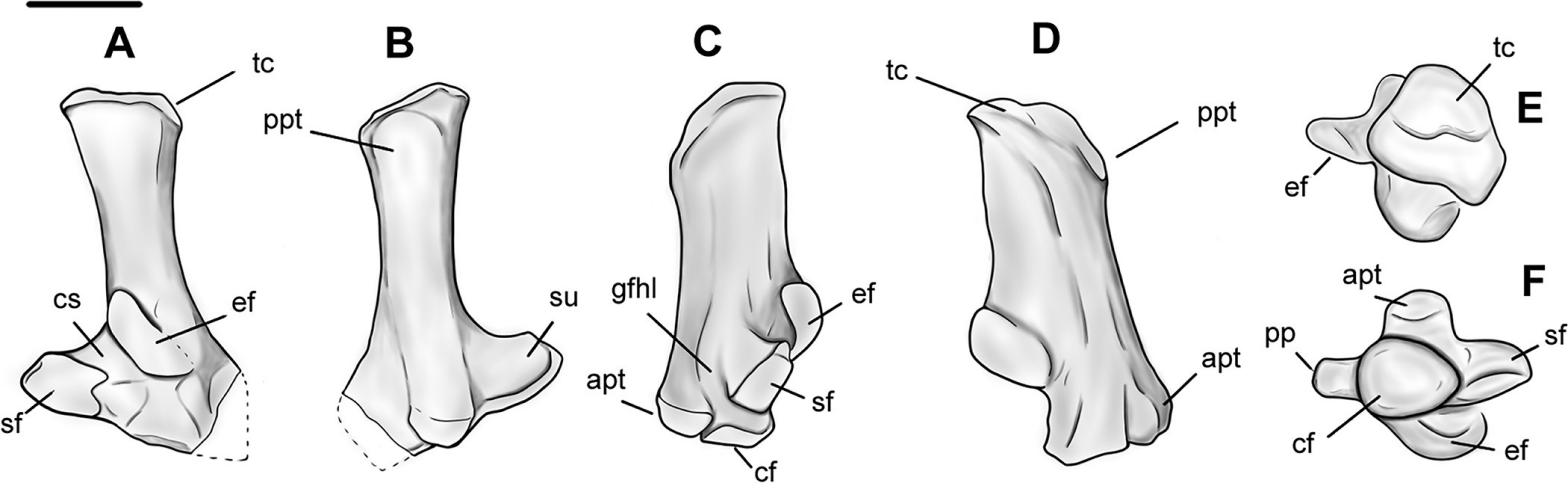
Drawing of the left calcaneum, *Conoryctes comma*, based on NMMNH P-48198 and NMMNH P-47866, in dorsal (A), plantar (B), medial (C), lateral (D), distal (E) and anterior (F) views. apt: anterior plantar tubercle, cf: cuboid facet, cs: calcaneal sulcus, ef: ectal facet, gfhl: groove for the flexor halluces longus tendon, pp: peroneal process, ppt: posterior plantar tubercle, sf: sustentacular facet, su: sustentaculum, tc: tuber calcanei. Scale bar is 1cm.

The calcaneum of *Conoryctes* is generally elongate and slender, with prominent facets anteriorly for articulation with the astragalus, fibula and cuboid (NMMNH P-48198, NMMNH P-48052, NMMNH P-47866, Figs 26 and 27). On the anterior part of the calcaneum, the articular facets are expanded mediolaterally. The cuboid facet is concave and is on the most anterior part of the calcaneum. In anterior view, the cuboid is separated from the other calcaneal facets. Plantarly the cuboid facet is separated from the anterior plantar tubercle by a smaller sulcus. The sustentaculum of *Conoryctes* extends medially, is oval, and mediolaterally-elongate. The sustentaculum is thick dorsoplantarly and is demarcated distally from the cuboid facet by a narrow groove, which continues plantarly. The flexor hallucis longus tendon passed through this groove and was involved in the flexion of the pedal digit I [39]. The sustentaculum is well separated from the ectal facet by the deep calcaneal sulcus, through which the interosseous astragalocalcaneal ligament and nerves passed (Fig 27A) [39]. Dorsally, there is an ovoid and shallowly concave sustentacular facet that connects with the plantar-medial part of the astragalar sustentacular facet. The peroneal process of *Conoryctes* is situated laterally; it originates more anteriorly than the sustentacular process and does not protrude laterally (Fig 26, NMMNH P-47866). The peroneal process is more than 25% of the total calcaneal length. In dorsal aspect, the peroneal process extends to the same level as the most anterolateral boundary of the cuboid facet. In *Conoryctes*, there is a wide and narrow groove that separates the peroneal process and the ectal facet. In anterior view, the peroneal process is next to the cuboid facet, but the two features do not meet. Plantarly, the peroneal process and the anterior plantar tubercle are separated by a wide and shallow canal. Through this groove passed the abductor digit V muscle, responsible for the abduction of the fifth metatarsal, and other ligaments, i.e., the calcaneoquartal and the lateral collateral ligaments [39].

The ectal facet is anterolaterally- to posteromedially-oriented, forming a 145° angle with the anteroposterior calcaneal axis; the facet is also mediolaterally elongate and rather convex (S13 Table). In dorsal aspect, the ectal facet reaches the medial border of the calcaneum but is separated from the tuber calcanei. The most anterior border of the ectal facet extends to the same level as the posterior borders of the sustentaculum and of the peroneal process. In medial aspect, between the ectal facet and the sustentaculum there is the calcaneal sulcus (Fig 27A). In lateral view, the ectal facet is separated from the peroneal process by a narrow canal, possibly for the medial collateral ligament, the extensor digitorum lateralis and the fibularis (peroneus) brevis muscles [39]. The fibular facet is small, convex and circular and is located almost vertically to the anterolateral border of the ectal facet (NMMNH P-48198).

*Conoryctes* has a distinctively protruding anterior plantar tubercle, which is long and cylindrical (Fig 26I–26P). In anterior aspect, it is medially-placed, closer to the sustentacular than the peroneal process, separated from these two processes by two grooves for the passing of muscles, nerves and ligaments. In medial view, the anterior plantar tubercle extends straight anteriorly and is not medioplantarly oblique like the cuboid facet. The anterior plantar tubercle and the cuboid facet are separated by a small fissure, and the cuboid facet extends slightly more anteriorly than the anterior plantar tubercle.

In *Conoryctes* the tuber calcanei is elongate, representing approximately 56% of the maximum anteroposterior calcaneal length, and forms a medially-inclined posterior plantar tubercle. The gastrocnemius muscle was attached to the calcaneal tuber and was responsible for the extension and flexion of the pes [39]. The flexor digitorum superficialis muscle, which flexes the II, III, IV and V digits, passed by the tuber calcanei [39]. *Conoryctes* has a flat and textured lateral side of the tuber calcanei. This could be due to the connection of the calcaneoquartal ligament that starts from the plantar-lateral surface of the calcaneum and attaches to the tarsal IV and the base of metatarsal V [39]. In distal aspect, the tuber calcanei has a shallow groove, mediolaterally-oriented for the calcaneal tendon.

*Conoryctes* has a similar calcaneum to *Onychodectes* (AMNH 27679), but less so to *Ectoganus* (USGS 3838) and cf. *Stylinodon mirus* (USNM 18425) [6, 7]. Proximally, the ectal facet is large and concave in all studied taeniodonts and is more mediolaterally-oriented in *Conoryctes* and *Onychodectes* than in *Ectoganus* and *Stylinodon*. In *Conoryctes*, the cuboid facet is medioplantarly oblique to the calcaneal long axis, whereas both the cuboid facet and the anterior plantar tubercle in *Onychodectes* are medioplantarly oblique to the calcaneal long axis. Szalay and Decker [51] considered this as a primitive eutherian condition and it is to some degree evident in *Procerberus* as well. The peroneal process of *Stylinodon* reaches the cuboid facet anteriorly, yet it is not as prominent as in *Conoryctes*. In *Conoryctes* and *Onychodectes* the tuber calcanei are wider dorsoplantarly than mediolaterally. The tuber calcanei of *Conoryctes* is slender and expanded more mediolaterally compared to *Onychodectes*. In *Conoryctes*, *Onychodectes* and *Stylinodon* the tuber calcanei shaft is almost 50% of the maximum anteroposterior calcaneal length (S13 Table). In medial aspect, *Onychodectes* has a more prominent posterior plantar tubercle at the distal end of the tuber calcanei than does *Conoryctes*.

The calcaneum of *Conoryctes* shares few similarities with *Procerberus* (AMNH 117455) [51]. In *Conoryctes*, and in other taeniodonts, the sustentaculum and peroneal processes are subequal, whereas in *Procerberus* the ectal facet is larger. The sustentaculum of *Procerberus* is less pronounced and is more circular than the oval-shaped sustentaculum of *Conoryctes*, and the peroneal process is proportionally larger [51]. In *Procerberus* the cuboid facet is shallowly concave, almost circular in profile and more anteriorly placed than in *Conoryctes* and other taeniodonts. In dorsal view, there is also a wide and deep calcaneal sulcus. The cuboid facet and the anterior plantar tubercle are also medioplantarly-oriented to some extent, similar to *Conoryctes*. The peroneal tubercle of *Conoryctes* is more prominent and reaches the same level anteriorly as the cuboid facet, unlike in *Procerberus*. In contrast to *Conoryctes*, the tuber calcanei of *Procerberus* is less than 50% of the total calcaneal length.

The calcaneum of *Conoryctes* is similar to *Escavadodon* (NMMNH P-22051), with a thick and prominent sustentacular facet that is closer to the cuboid facet anteroposteriorly [34]. Leptictids, however, have a less medially-protruding sustentacular facet, almost circularly-shaped, more posteriorly-located relative to the cuboid facet, and a less pronounced anterior plantar tubercle [30]. In leptictids, the cuboid facet tapers medioplantarly towards the planter tubercle more so than in specimens of *Conoryctes*. In dorsal aspect, the posterior apex of the tuber calcanei in *Conoryctes*, *Escavadodon* and leptictids is inclined medially [34]. Unlike *Conoryctes* and other taeniodonts, the tuber calcanei in *Escavadodon* and leptictids is less than half the total calcaneal length, similar to *Procerberus* [51].

Comparing the calcaneum of *Conoryctes* to *Periptychus* (NMMNH P-47693) [35] and *Pantolambda* (AMNH 16663) [16, 35, 49], there are a few differences. Anteriorly, the ectal facet is more elongate in *Conoryctes*, less so in *Periptychus* and more oval in *Pantolambda*. In all three genera though, the ectal facet is more mediolaterally-oriented than anteroposteriorly. The sustentacular facet extends less medially in *Conoryctes* and *Periptychus* than in *Pantolambda*. The cuboid facet bulges anterodorsally and tapers medioplantarly in both genera of *Conoryctes* and *Periptychus*. Unlike *Conoryctes*, the anterior plantar tubercle does not reach the cuboid facet anteriorly in *Periptychus*. However, the peroneal process is more robust in *Pantolambda* than in *Periptychus*, while it is much larger in *Conoryctes*. The posterior plantar tubercle is more prominent in *Conoryctes* and *Periptychus*.

### Other pedal elements

**Fig 28:**
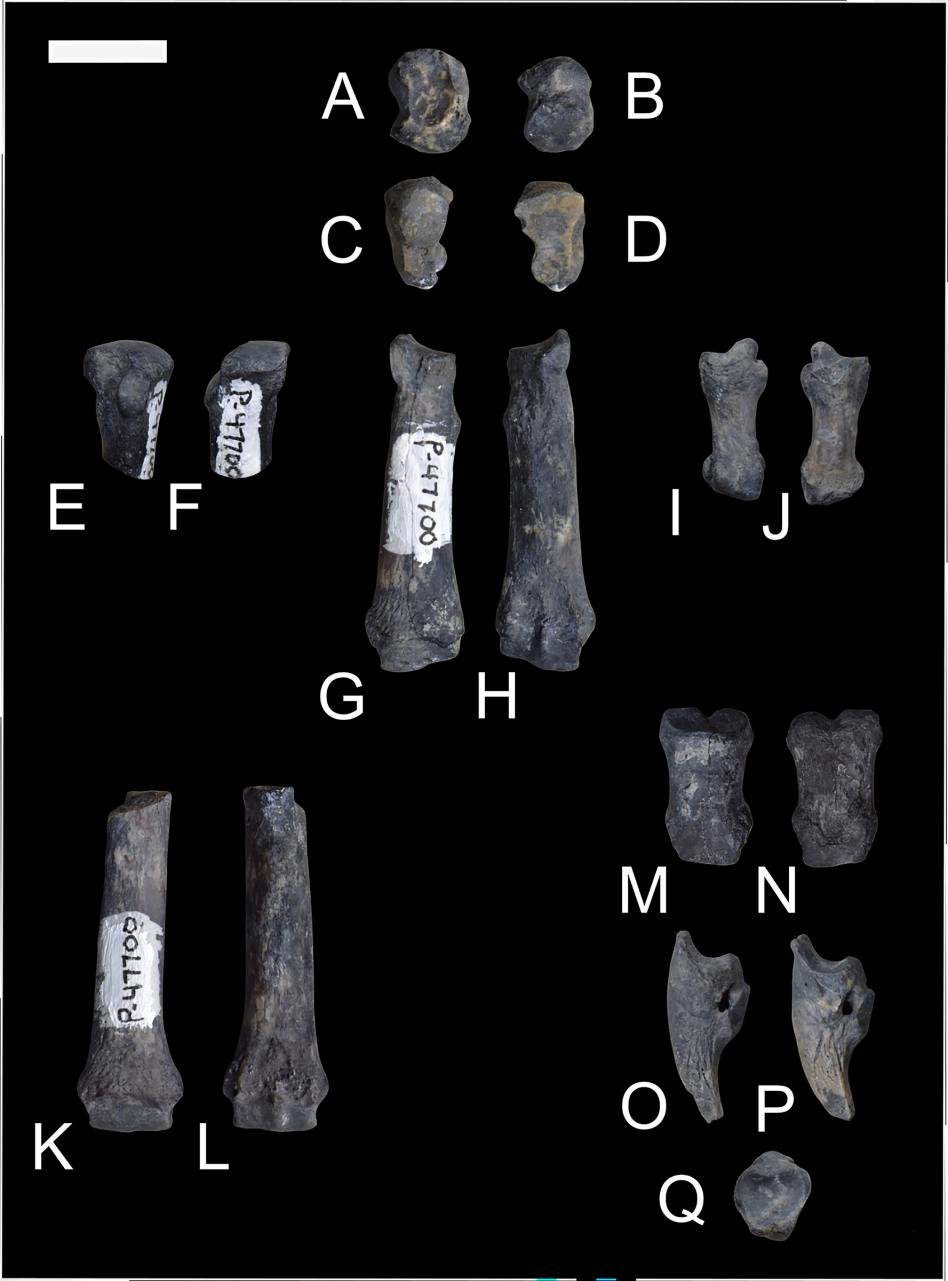
Part of the pes of *Conoryctes comma* (NMMNH P-47700). Navicular (A, B) in proximal (A) and distal (B) views, ectocuneiform (C, D) in proximal (C) and distal (D) views; proximal metatarsal III in dorsal (E) and ventral (F) views; complete metatarsal IV in dorsal (G) and ventral (H) views; metatarsal V in dorsal (I) and ventral (J) views; partial metatarsal in dorsal (K) and ventral (L) views; phalanges in dorsal (M) and ventral (N) views; ungual in medial (O, and proximal (Q) views; ungual in medial view (P). Scale bar is 1cm.

Part of the pes of *Conoryctes* is known from NMMNH P-47700 (Fig 28). This specimen includes parts of the tarsals (with ectocuneiform and navicular), the proximal part of metatarsal III, complete metatarsals IV and V, and a partial metatarsal, as well as two distal phalanges. In general, the metatarsals of *Conoryctes* are more slender than the elements of the manus and have less prominent flexor tubercles (S14 Table). The ectocuneiform (Fig 28A and 28B) and navicular (Fig 28C and 28D) are complete. The navicular is proximodistally short with a deeply concave navicular facet. Distally there are three distinct facets for the articulation with the cuneiform. The ectocuneiform is longer plantodorsally than mediolaterally. It has two facets where it articulates with the navicular and there is a lateral facet for the cuboid. Distally the ectocuneiform is curved and articulates with a concave proximal facet of metatarsal III.

The metatarsals are proximodistally long and are concave on the plantar aspect. Distally the metatarsals have a similar ridge dorsoplantarly as the metacarpals. There is a groove on the proximal end of the first phalanges of the pes (Fig 28G–28J) providing extra stability to the metatarsal-phalanx joint [50]. The unguals of the pes are morphologically similar to the manual unguals, having a deep curvature proximally with a “bony stop” and plantar flexor tubercles and being recurved and laterally compressed (Fig 28M–28Q).

Compared with other taeniodonts the pes of *Conoryctes* is similar to *Onychodectes* (AMNH 16528) in shape and size [6]. The dorsoplantar ridges on the distal articulation of the metatarsals are less prominent in *Onychodectes* than in *Conoryctes*. The pes of *Psittacotherium* (AMNH 16560) and *Stylinodon* (UW 2270) have more robust and mediolaterally-wide metatarsals, with longer and more curved unguals [6].

The metatarsals of *Conoryctes* resemble those of *Escavadodon* (NMMNH P-22051) [34] in being concave on the plantar aspect. *Escavadodon*, as well as *Periptychus* (AMNH 17075) [35] and *Pantolambda* (AMNH 16663) [35, 49], differ from *Conoryctes* because they lack the dorsoplantar ridges on the distal articulation of the metatarsals. *Conoryctes* has laterally-compressed, curved unguals compared to the hoof-like rounded unguals with a median fissure of *Periptychus* [35] and the mediolaterally-broad unguals of *Pantolambda* [35, 49].

## Discussion

The new fossils of *Conoryctes comma* from the San Juan Basin, USA shed detailed light for the first time on its postcranial skeleton. *Conoryctes* was a medium-sized mammal, with a robust humerus, radius, and femur, unfused radius-ulna and tibia-fibula, and sharp claws. Postcranial elements are rare for taeniodonts, but the present study has indicated that there are many anatomical features that are shared among all known members of the group. All taeniodonts, for which reasonable postcranial skeletons are known, share these common features:

- The greater tubercle of the humerus extends to the same level as the humeral head, and in the deltopectoral region of the humerus, the distal part is mediolaterally-expanded [6, 7].
- The distal radial diaphysis is mediolaterally-broad [3, 6, 7].
- The femoral head extends more proximally than the greater trochanter, and the femoral trochlea reaches to the same level as the posterior part of the femoral condyles [3, 6, 7, 15].
- The medial malleolus of the tibia is well-developed and expands distally with a deep, well-defined sulcus for the digital flexor muscles [3, 6, 7, 13].
- The astragalus lacks a dorsal astragalar foramen, and the astragalar trochlea is U-shaped with subequal, prominent astragalar trochlear rims, while the astragalar neck is mediolaterally-narrow [6, 7, 13].
- The sustentacular facet of the calcaneum is mediolaterally broad [6, 7].
- The unguals of the manus and pes, are laterally compressed to form sharp claws and have well-developed flexor tubercles [3, 6, 7].

When compared to other Paleocene mammals, the postcranial skeleton of *Conoryctes* bears notable similarities with *Procerberus*, *Escavadodon* and leptictids [1, 30, 34, 51]. Both *Conoryctes* and *Escavadodon* possess a robustly built humerus; the greater tuberosity and the humeral head are almost on the same level and there is a well-developed deltopectoral region for attachment of large pectoralis and deltoid muscles. The ulnar radial notch of *Conoryctes* and palaeanodonts is placed laterally, and the flexor fossa is deep in both *Conoryctes* and *Escavadodon*. The distal shaft of the radius is also similar between *Conoryctes*, *Escavadodon* and primitive palaeanodonts, having a prominent pronator crest for the attachment of the pronator teres and pronator quadratus muscles, assisting in the pronation of the forearm [39]. In distal view, the carpal facets are not well-defined in *Conoryctes*, *Escavadodon*, leptictids and palaeanodonts. The femora of *Conoryctes* and palaeanodonts have a large fovea capitis with a posteromedial groove, and the third trochanter of *Conoryctes* and *Escavadodon* has a crest-like shape. The tibial shaft of *Conoryctes*, *Escavadodon*, *Prodiacodon* and *Leptictis* is slender with a well-developed flexor sulcus posteriorly for the digital flexor muscles. On the astragalar head, *Conoryctes*, *Procerberus*, *Escavadodon* and *Palaeanodon* have no dorsal foramen of the astragalar canal, potentially enabling unrestricted movement of the ankle during flexion [51]. The metatarsals of *Conoryctes* and *Escavadodon* are concave in planter aspect. Based on limb proportions and the robustness of the forelimb, Rose and Lucas [34] suggested that *Escavadodon* was a fossorial animal. Similarly, leptictids and palaeanodonts show morphological evidence for fossorial and saltatorial locomotion [30, 33, 34].

*Conoryctes* shares fewer similarities with *Periptychus* and *Pantolambda* in their postcranial anatomy [16, 35, 49], reinforcing the diversity of postcranial anatomy in Paleogene ‘archaic’ mammals. The humeral deltopectoral region is prominent in both *Conoryctes* and *Pantolambda*. The radius of *Conoryctes*, *Periptychus* and *Pantolambda* is mediolaterally-broader and has a laterally-expanding capitular eminence. Comparing the innominate of *Conoryctes* and *Pantolambda*, the ilium and ischium are approximately on the same plane. In *Conoryctes* and *Periptychus*, the fovea capitis has a groove that excavates the articular surface of the femoral head, and the lesser trochanter protrudes posteromedially and is triangular. The femoral trochlea ends at the most proximal end of the condyles in both *Conoryctes* and *Pantolambda*. In *Conoryctes*, *Periptychus* and *Pantolambda* the tibia and fibula are unfused proximally and distally. The astragalar head of *Conoryctes* and *Periptychus* have rims that are parallel and subequal in size. Shelley *et al*. [35] suggested that *Periptychus* was a terrestrial animal based on its postcranial anatomy.

### Functional morphology

Many taeniodonts, especially the Paleogene stylinodontids, have anatomical adaptations that point to fossoriality and scratch-digging behaviours [3, 6, 7, 52]. Modern digging mammals have osteological specialisations, such as robustness of the forelimb for the attachment of powerful muscles, related to various fossorial adaptations in order to loosen the substrate, to move the soil, to stabilize the body and prevent hyperextension of the joints while digging [e.g.,^10^, ^30^, ^50^, ^53^–^63^]. Animals can have different types of fossorial lifestyles and behaviours, depending on how they use different parts of their bodies to loosen the soil, flex their limbs against the resistance of the substrate, and move the soil [50]. Based on the muscle structure and the osteological characteristics, Hildebrand [50] proposed six categories of digging; scratch-digging, chisel-tooth digging, head-lift digging, hook-and-pull digging, humeral-rotation digging, and hind-feet-first digging. For the first four categories, animals have strong muscles for flexing and extending the forelimb and sometimes the hind limb. Additionally, chisel-tooth digging as well as head-lift digging requires strong neck muscles, whereas scratch-digging and hook-and-pull digging requires strong flexor muscles for the claws [50]. The humeral-rotation digging style is used by highly specialized digging animals, like moles and echidnas, with long scapulae for the shoulder medial rotators, and very mediolaterally-broad humeri for powerful teres major muscles and elbow adductors [50, 63]. During hind-feet-first digging the hindlimb is extended and the tarsus is flexed [50].

For scratch-digging mammals, these adaptations are mostly seen in the forelimb for stabilizing the shoulder, extending the elbow, pronating and supinating the forearm, flexing the wrist and the digits, and extending the elbow [50, 54]. The musculoskeletal adaptations to assist a scratch-digging mode of life are associated with large muscle masses and attachments for the origins and insertions for powering the forelimb, as well as anteroposteriorly-shorter distal elements of the manus and deep attachments to prevent hyperextension and dislocation of the distal phalanges [50, 54, 55]. Particularly, the retraction of the arm during digging is important for scratch-digging animals, thus the shoulder joint is characterized by large attachment areas for the teres major and latissimus dorsi muscles [50, 64]. At the elbow joint there are attachments for large forearm pronator teres muscles, which assist in the pronation and flexion of the forearm. There are also attachments for large carpal and digital flexor muscles, which are essential for scratch-digging animals that draw their claws when digging soil [50, 64]. To enhance the extension of the forearm, there are also powerful triceps brachii muscles at the elbow joint [50, 64].

These features are reflected in the osteological anatomy of scratch-diggers which have large areas for muscle attachments on the scapula and humerus, a narrow and elongate scapula, a robust humerus with a medially broad medial epicondyle and a strong deltopectoral crest, a relatively long ulnar olecranon, relatively short forearms, robust metacarpals, proximal and medial phalanges with bony stops, and either sharp and mediolaterally narrow claws to dig through hard substrate or wider claws for softer soil [50, 53–55, 58, 62, 65]. As a mechanical enhancement for applying high out-force, these animals have an elongate ulnar olecranon. Large palmar sesamoids protect the flexor tendons which are important for scratch-digging [50, 65].

*Conoryctes* and other conoryctids were believed to lack postcranial specializations for digging or any other distinctive lifestyle and were instead thought to be “generalised” [1, 6, 8]. Other researchers later suggested that conoryctids could have some level of fossoriality for feeding purposes [9]. Williamson and Brusatte [3] described the digging adaptations in *Wortmania*, some of which are seen in *Onychodectes*, and thus hypothesized that all conoryctids, and possibly all taeniodonts, were well-adapted for a digging lifestyle. The new specimens from the San Juan Basin provide evidence that *Conoryctes* was a digging animal, although not as highly specialized for fossoriality as later derived taeniodonts like *Stylinodon*.

*Conoryctes* had a short neck with anteroposteriorly-short proximal cervical vertebrae (S3 Table). Many fossorial animals like hedgehogs, have short necks to strengthen their vertebral column [53] making their body stouter and more robust. The tail of *Conoryctes* is long and robust proximally, which is well-suited to assist with stabilizing its hind body while digging with its forelimbs [50]. Although a short tail is usually seen in digging animals, there are semi-fossorial mammals, such as aardvarks, that have long tails [53]. Similarly, the sacrum of digging animals usually consists of more than four fused sacral vertebrae, since a longer sacrum assists in the stability of the pelvis [50, 66]. The sacrum of *Conoryctes* consists of only three fused vertebrae, but it is elongate.

The forelimb of *Conoryctes*, known from the humerus, radius, and proximal ulna, is short and stout, similar to other fossorial animals [54, 59]. Based on the newly referred proximal humerus (NMMNH P-48052) and isolated humeri fragments (NMMNH P-61789 and NMMNH P-77896), the humeral head is hemispherical, and the humeral index (humeral head mediolateral width/proximodistal length) is high (91.9%), indicating a more mobile glenohumeral joint [59]. The greater tuberosity is well-developed for the attachment of infraspinatus and supraspinatus muscles, which are responsible for the retraction and protraction of the humerus [39]. The subscapularis muscle, which attached to the lesser tuberosity, was responsible for the adduction of the shoulder joint and for assisting in the medial rotation of the humerus [39]. The humeral shaft has a long distally-extended and mediolaterally-broad deltopectoral area where powerful deltoid and pectoral muscles attached for the rotation and abduction, and the adduction and retraction of the humerus, respectively [39]. The distal end of the humerus is broad, and the medial epicondyle is very prominent indicating strong digital and carpal flexors and strong extensors muscles on the lateral epicondyle. The robust deltopectoral area and the medially-broad distal humerus are also seen in many modern fossorial animals, such as armadillos [62], pangolins [63] and burrowing squirrels [64]. The anteriorly-projecting deltopectoral crest and the mediolaterally-broad distal epiphysis with a moderate entepicondylar foramen of *Conoryctes* match the description of the “primitive fossorial type” proposed by Gregory [67]. These humeral features of *Conoryctes* are also seen in more derived taeniodonts, such as *Psittacotherium*, *Ectoganus* and *Stylinodon*, that were interpreted as adapted for digging [6, 7].

The olecranon of *Conoryctes* is elongate and medially-curved, and a deep flexor fossa indicates powerful digital flexors, such as the flexor digitorum profundus muscle and forearm extensors, such as the triceps brachii muscle [39]. On the lateral side, the ulna of *Conoryctes* has a deep fossa for well-developed abductors. The flat ulnar facet on the radius of *Conoryctes* allowed limited supination of the forelimb and is a feature seen in other taeniodonts including *Onychodectes* and *Wortmania*. A flat ulnar facet has been associated with terrestrial or digging behaviours [30]. The radius is very stout and is anteriorly-curved with a strong pronator crest, a moderate styloid process, providing additional support in the wrist joint, and separated facets distally for the lunar and scaphoid facets. The pronator crest is where the pronator teres and the pronator quadratus muscles, responsible for assisting in the pronation of the forearm, attached [39].

The metacarpals of *Conoryctes* have large extensor tubercles on the dorsal surface for the stabilization of the metacarpal-proximal phalanx joints. The metacarpals and phalanges are curved distally and have a prominent ridge on the plantar surface to prevent the dislocation and hyperextension of the phalanges [50]. This anatomy of the metacarpals and phalanges is also seen in other plantigrade animals, as well as in *Prodiacodon*, *Escavadodon* and all taeniodonts with known postcranial elements [3, 34]. The unguals of *Conoryctes* are enlarged, laterally-compressed, bear large flexor tubercles, and have a curved articular surface for the intermediate phalanx. The dorsoventral depth and the curvature of the unguals are also similar to those of fossorial animals [57]. Moreover, in *Conoryctes* the most proximal and intermediate phalanges are short while the unguals are elongate, a common feature observed in extant fossorial mammals [59, 64].

The tibial crest of *Conoryctes* is long (Figs 21–23) relative to the total length of the tibia, indicative of powerful knee flexion seen in digging animals [59]. The anteroposteriorly-short astragalar neck relative to the total length of the astragalus (S12 Table) and the elongate tuber calcanei (measurement 10, S6 Fig) are anatomical features seen in many taeniodonts, including *Conoryctes* and in digging animals [59]. The metatarsals of *Conoryctes* are similar to the metacarpals, having flexor tubercles and being curved posteriorly. The unguals of the pes are also similar to the unguals of the manus, having enlarged flexor tubercles.

Following Hildebrand’s [50] categories of digging behaviour, we interpret *Conoryctes* as a fossorial animal with specializations for forelimb digging, without any extreme rotation of the forearm as in humeral-rotation diggers. The lack of strong dorsal neck muscles and enlarged incisors indicate that *Conoryctes* was not a chisel-tooth or head-lift digger [50], although its enlarged canines may have had some use in loosening soil. The powerful forearm muscles for the flexion and extension of the humerus and the elbow, and strong and well-stabilised digits, indicate that it was probably a scratch-digging or hook-and-pull digging animal [50]. Both digging styles rely on strong shoulder retractors, with hook-and-pull diggers relying more on elbow flexors and scratch-diggers on elbow extensors [50, 63]. *Conoryctes* has a much deeper ulnar fossa for the abductor pollicis longus muscle and a relatively wide area for the triceps brachii muscle on the olecranon, compared to the fossae for the biceps brachii and brachialis muscles (Fig 10). Therefore, it is more likely that *Conoryctes* was extending its forelimb using these powerful elbow extensor muscles and then using its claws to dig into the soil or substrate [50, 63].

As demonstrated in this study, there are many similarities between the postcranial skeleton of *Conoryctes* and *Onychodectes*, i.e., the robust deltopectoral crest area, the medial extension of the distal humerus, the medially-oriented and long olecranon process of the ulna and the laterally-compressed recurved unguals. All these anatomical features indicate digging abilities for *Onychodectes*, as suggested by previous studies [3]. However, *Conoryctes* has less extreme digging adaptations than in stylinodontids such as *Wortmania* and *Psittacotherium*. Therefore, this study strengthens the suggestions proposed by Williamson and Brusatte [3] that all taeniodonts have adaptations for at least some degree of fossoriality, from the most basal taxa like *Onychodectes* and the members of Conoryctidae, to Stylinodontidae and the most derived taxa like *Stylinodon*. Taking into account the phylogeny proposed by the same study [3], it is possible that these adaptations were present in the most recent common ancestor of the clade and potentially assisted in the radiation of taeniodonts after the end-Cretaceous extinction.

Schoch pointed out functional similarities between Stylinodontidae and *Orycteropus*, a scratch-digging animal, and proposed a similar palaeobiology for *Stylinodon* [6]. Some extant fossorial animals that exhibit a scratch-digging behaviour are armadillos [50, 62, 63], pangolins [50, 63], ground squirrels (*Spermophilus citellus*) [50, 64], aardvarks [50, 63] and badgers [63, 68]. The digging ability of animals from these groups varies, and studies have tended to focus on the shape and the muscle mechanisms of the forearm [62–64]. Comparing the anatomy of the forelimb, *Conoryctes* is more morphologically similar to moderately-specialized scratch-digging animals than to the previously proposed highly specialized aardvark [6, 50, 69]. The flexor fossa, on the lateral side of the ulnar shaft, is deep in *Conoryctes*, similar to the aardvark and the badger *Mellivora* [70]. The deltopectoral crest expands more distally close to the entepicondylar foramen in *Conoryctes*, pangolins, and some armadillos, but is distinct from aardvarks and the less robust humerus of *Mellivora* [50, 62, 63, 69, 70]. The medial epicondyle flares significantly more than the lateral one, leading to more effective force of the pronator teres muscle in pangolins, aardvarks and some armadillos [50, 62, 69], whereas it is less medially-protruding in *Conoryctes*. A similarity between pangolins and *Conoryctes* that aardvarks and *Mellivora* lack is a robust anteriorly-expanding shaft of the radius, which increases the area for attachment of the extensor metacarpi pollicis muscle [39]. Furthermore, in *Conoryctes* and pangolins the manus has mechanisms against the hyperextension and dislocation of the digits: the phalanges have a greater radius of curvature proximally and distally large median keels only on the plantar surface, and the unguals are deeply-grooved with bony stops [50, 57, 63].

Therefore, we infer that *Conoryctes* was a fossorial animal which removed soil close to the surface of the ground, probably mostly to procure food, using its strong extensor muscles, but it was not as powerful a digger as some other scratch-digging animals, like the aardvark [69].

## Conclusion

New fossils from the Paleocene of the San Juan Basin of New Mexico, USA provide important anatomical information about the postcranial skeleton of the taeniodont mammal *Conoryctes comma*. Regarding functional morphology, there are many indications that *Conoryctes* was not an animal with a generalised, primitive body plan, as previously thought for all conoryctids [8]. On the contrary, *Conoryctes* was larger in size than other conoryctids and had a high level of fossoriality. Notably, we identify anatomical features which strongly indicate that *Conoryctes* was a scratch-digging animal [50, 63] with powerful forearm muscles and well-stabilized digits, able to dig and survive in the subtropical forest of western North America approximately 63 million years ago. This corroborates the study by Williamson and Brusatte [3] which suggested that digging adaptations are present in all members of Taeniodonta for which the postcranial elements are known.

## Supporting information

S1 Table A list of the new specimens studied from the San Juan Basin of *Conoryctes comma*, the localities they were found and their biozones. (DOCX)

S2 Table Teeth measurements of NMMNH P-19494, *Conoryctes comma*, from the San Juan Basin. (DOCX)

S3 Table Measurements of the vertebrae of *Conoryctes comma*. (DOCX)

S4 Table Measurements of the humerus from the new specimens of *Conoryctes comma*. (DOCX)

S5 Table Measurements of the ulna of *Conoryctes comma*. (DOCX)

S6 Table Measurements of the radius of *Conoryctes comma*. Numbers are referring to the measurements as seen in S1 Fig.
(DOCX)

S7 Table Measurements of the metacarpals of *Conoryctes comma*. (DOCX)

S8 Table Measurements of the pelvis of *Conoryctes comma*. Numbers are referring to the measurements as seen in S2 Fig.
(DOCX)

S9 Table Measurements of the femur of *Conoryctes comma*. Numbers are referring to the measurements as seen in S3 Fig.
(DOCX)

S10 Table Measurements of the patella of *Conoryctes comma*. (DOCX)

S11 Table Measurements of the tibia of *Conoryctes comma*. Numbers are referring to the measurements as seen in S4 Fig.
(DOCX)

S12 Table Measurements of the astragalus of *Conoryctes comma*. Numbers are referring to the measurements as seen in S5 Fig.
(DOCX)

S13 Table Measurements of the calcaneum of *Conoryctes comma*. Numbers are referring to the measurements as seen in S6 Fig.
(DOCX)

S14 Table Measurements of the metatarsals of *Conoryctes comma*. (DOCX)

S1 Fig Drawing of the measurements taken of the radius in posterior (A) and lateral (B) views. Total proximodistal length (1), mediolateral width of the proximal epiphysis (2), mediolateral width of the distal epiphysis (3), mediolateral width at the middle of the shaft (4), anteroposterior width of the proximal epiphysis (5), anteroposterior width at the middle of the shaft (6), anteroposterior width of the distal epiphysis (7), anteroposterior width of the distal articular fovea (8).
(TIFF)

S2 Fig Drawing of the measurements taken of the pelvis in lateral (A) and medial (B) views. Length of the lunate surface (1), width of the lunate surface (2), length from the tip of the ischiatic tuberosity to the iliopectineal eminence (3), total length of the fossae attaching the sacrum on the ventral view of the ilium (4), length of the fossa closer to the greater ischiatic notch (5), length of the fossa on the wing of the ilium (6).
(TIFF)

S3 Fig Drawing of the measurements taken of the femur in anterior (A) and proximal (B) views. Total mediolateral width of the proximal epiphysis (1), femoral head anteroposterior length (2), femoral head mediolateral width (3), femoral head proximodistal length (4).
(TIFF)

S4 Fig Drawing of the measurements taken of the tibia in anterior (A), proximal (B) and distal (C) views. Total proximodistal length (1), proximal tibia mediolateral width (2), distal tibia mediolateral width (3), proximal fibular facet mediolateral width (4), proximal fibular facet anteroposterior length (5), distal tibia anteroposterior total length (6), proximal tibia anteroposterior total length (7).
(TIFF)

S5 Fig Drawing of measurements of the astragalus in dorsal (A), plantar (B), medial (C) and lateral (D) views. Mediolateral total width of the astragalar body (1), mediolateral total width of the astragalar head (2), anteroposterior length of the astragalar neck and head (3), mediolateral total width of the astragalar neck (4), mediolateral total width of the anterior most edge of the astragalar body (5), mediolateral total width of the posterior most edge of the astragalar body (6), anteroposterior total length of the astragalus (7), anteroposterior length of the ectal facet (8), mediolateral width of the ectal facet (9), anteroposterior length of the sustentacular facet (10), mediolateral width of the sustentacular facet (11), anteroposterior length of the medial tibial facet (12), dorsoplantar width of the medial tibial facet (13), dorsoplantar width of the astragalar head (14), anteroposterior length of the lateral tibial facet (15), dorsoplantar width of the lateral tibial facet (16).
(TIFF)

S6 Fig Drawing of the measurements taken on the calcaneum in dorsal (A), medial (B) and anterior (C) views. Total anteroposterior length (1), total mediolateral width between the sustentacular facet and the peroneal process (2), anteroposterior length of the ectal facet (3), mediolateral width of the ectal facet (4), anteroposterior length of the sustentacular facet (5), mediolateral width of the sustentacular facet (6), mediolateral width of the cuboid facet (7), dorsoplantar length of the cuboid facet (8), anteroposterior length of the peroneal process (9), distance the tuber calcanei to the most posterior edge of the ectal facet (10), distance between the most anterior edge of the ectal facet and the most anterior part of the calceneum (11), distance between the most posterior edge of the ectal facet and the most anterior part of the calceneum (12), mediolateral width of the tuber calcaneum at the middle point (13), dorsoplantar length of the anterior edge of the calcaneum (14), dorsoplantar length at the middle point of the calcaneum (15), dorsoplantar length of the tubercle calcanei (16), mediolateral width of the anterior plantar tubercle (17).
(TIFF)
