# Supplementary material for: The post-cranial anatomy and functional morphology of Conoryctes comma (Mammalia: Taeniodonta) from the Paleocene of North America
Source: PLoS One. 2024 Oct 25;19(10):e0311053. doi: 10.1371/journal.pone.0311053 (PMC11508153; doi:10.1371/journal.pone.0311053)
Supplement: S1 Table — (DOCX) [file pone.0311053.s001.docx]

**S1 Table.**

| **Specimen number** | **Associated elements** | **Locality** | **Biozone** |
| --- | --- | --- | --- |
| **NMMNH P-19494** | M1, M2, p4, distal tibia, partial femur | Upper horizon of West Flank of Torreon Wash | Tj6 |
| **NMMNH P-48198** | Vertebrae, os coxae, tibia, astragalus, calcaneum | Upper horizon of West Flank of Torreon Wash | Tj6 |
| **NMMNH P-48052** | Atlas, vertebrae, ribs, proximal ulna, metacarpals, phalanges and ungual, proximal and distal tibia, parts of proximal and distal femur, patella, astragalus, calcaneum | Upper horizon of West Flank of Torreon Wash | Tj6 |
| **NMMNH P-61789** | Partial humerus, partial os coxae | Upper horizon of West Flank of Torreon Wash | Tj6 |
| **NMMNH P-77896** | Distal humerus, partial os coxae | Upper horizon of West Flank of Torreon Wash | Tj6 |
| **NMMNH P-21509** | Vertebrae, proximal tibia, astragalus | Upper horizon of East Flank of Torreon Wash | Tj6 |
| **NMMNH P-79457** | vertebrae, sacrum, radius, metacarpals, phalanges and ungual, proximal femur | Red Mesa | Tj5 |
| **NMMNH P-47700** | Vertebrae, partial humerus, partial os coxiae, partial tibia, metatarsals, phalanges and unguals | Angel Peak, Kutz Canyon | Tj4 |
| **NMMNH P-47866** | calcaneum | Angel Peak, Kutz Canyon | Tj4 |
