# Supplementary material for: The post-cranial anatomy and functional morphology of Conoryctes comma (Mammalia: Taeniodonta) from the Paleocene of North America
Source: PLoS One. 2024 Oct 25;19(10):e0311053. doi: 10.1371/journal.pone.0311053 (PMC11508153; doi:10.1371/journal.pone.0311053)
Supplement: S2 Table — (DOCX) [file pone.0311053.s002.docx]

**S2 Table.**

| **Teeth** | **Buccallingual width (mm)** | **Anteroposterior length (mm)** |
| --- | --- | --- |
| **M1** | 13.82 | 8.31 |
| **M2** | 13.34 | 7.61 |
| **p4** | 8.21 | 5.91 |
