# Supplementary material for: The post-cranial anatomy and functional morphology of Conoryctes comma (Mammalia: Taeniodonta) from the Paleocene of North America
Source: PLoS One. 2024 Oct 25;19(10):e0311053. doi: 10.1371/journal.pone.0311053 (PMC11508153; doi:10.1371/journal.pone.0311053)
Supplement: S3 Table — (DOCX) [file pone.0311053.s003.docx]

**S3 Table.**

| **Specimen** |  | **mm** |
| --- | --- | --- |
| **NMMNH P-21509** | Caudal vertebra body anteroposterior length | 21.31 |
|  | Caudal vertebra body mediolateral width | 15.61 |
| **NMMNH P-48052** | Axis body anteroposterior length | 12.57 |
|  | Axis body mediolateral width | 16.33 |
|  | Caudal body anteroposterior length | 26.24 |
|  | Caudal body mediolateral width | 16.94 |
| **NMMNH P-79457** | Cervical body mediolateral width | 12.57 |
|  | Cervical body anteroposterior length | 7.71 |
|  | Thoracic body mediolateral width | 13.59 |
|  | Thoracic body anteroposterior length | 10.61 |
|  | Lumbar body mediolateral width | 17.48 |
|  | Lumbar body anteroposterior length | 19.06 |
|  | Sacral vertebra body mediolateral width | 54.41 |
|  | Sacral vertebra body anteroposterior length | 41.35 |
| **NMMNH P-48198** | Lumbar body mediolateral width | 21.75 |
|  | Lumbar body anteroposterior length | 20.76 |
|  | Caudal body mediolateral width | 15.12 |
|  | Caudal body anteroposterior length | 18.42 |
| **NMMNH P-47700** | Cervical body mediolateral width | 14.6 |
|  | Cervical body anteroposterior length | 8.04 |
|  | Lumbar body mediolateral width | 15.14 |
|  | Lumbar body anteroposterior length | 16.64 |
|  | Caudal body mediolateral width | 14.3 |
|  | Caudal body anteroposterior length | 25.38 |
