# Supplementary material for: The post-cranial anatomy and functional morphology of Conoryctes comma (Mammalia: Taeniodonta) from the Paleocene of North America
Source: PLoS One. 2024 Oct 25;19(10):e0311053. doi: 10.1371/journal.pone.0311053 (PMC11508153; doi:10.1371/journal.pone.0311053)
Supplement: S4 Table — (DOCX) [file pone.0311053.s004.docx]

**S4 Table.**

| **Specimen** |  | **mm** |
| --- | --- | --- |
| **NMMNH P-48052** | Humeral head mediolateral width | 13.22 |
|  | Humeral head proximodistal length | 14.39 |
| **NMMNH P-77896** | Maximum distal mediolateral width | 53.31 |
|  | Maximum distal humeral  trochlea width | 9.8 |
