# Supplementary material for: The post-cranial anatomy and functional morphology of Conoryctes comma (Mammalia: Taeniodonta) from the Paleocene of North America
Source: PLoS One. 2024 Oct 25;19(10):e0311053. doi: 10.1371/journal.pone.0311053 (PMC11508153; doi:10.1371/journal.pone.0311053)
Supplement: S6 Table — Numbers are referring to the measurements as seen in S1 Fig. (DOCX) [file pone.0311053.s006.docx]

**S6 Table.**

| **Specimen** |  | **mm** |
| --- | --- | --- |
| **NMMNH P-79457** | Total proximodistal length (1) | 56.12* |
|  | Mediolateral width of the proximal epiphysis (2) | 10.27 |
|  | Mediolateral width of the distal epiphysis (3) | 11.18 |
|  | Mediolateral width at the middle of the shaft (4) | 7.97* |
|  | Anteroposterior width of the proximal epiphysis (5) | 7.56 |
|  | Anteroposterior width at the middle of the shaft (6) | 4.83* |
|  | Anteroposterior width of the distal epiphysis (7) | 10.89 |
|  | Anteroposterior width of the distal articular fovea (8) | 6.93 |
