# Supplementary material for: The post-cranial anatomy and functional morphology of Conoryctes comma (Mammalia: Taeniodonta) from the Paleocene of North America
Source: PLoS One. 2024 Oct 25;19(10):e0311053. doi: 10.1371/journal.pone.0311053 (PMC11508153; doi:10.1371/journal.pone.0311053)
Supplement: S7 Table — (DOCX) [file pone.0311053.s007.docx]

**S7 Table.**

| **Specimen** |  | **mm** |
| --- | --- | --- |
| **NMMNH P-79457** |  |  |
| **II metacarpals** | Total length | 14.04 |
|  | Perimeter at midshaft | 18 |
|  | Mediolateral width of the proximal epiphysis | 5.24 |
|  | Mediolateral width of the distal epiphysis | 7.34 |
|  | Anteroposterior width of the proximal epiphysis | 6.19 |
|  | Anteroposterior width of the proximal epiphysis | 5.21 |
| **V metacarpals** | Total length | 13.84 |
|  | Perimeter at midshaft | 17 |
|  | Mediolateral width of the proximal epiphysis | 5.92 |
|  | Mediolateral width of the distal epiphysis | 6.09 |
|  | Anteroposterior width of the proximal epiphysis | 4.69 |
|  | Anteroposterior width of the proximal epiphysis | 4.80 |
| **NMMNH P-48052** |  |  |
| **II metacarpals** | Total length | 14.2 |
|  | Perimeter at midshaft | 19 |
|  | Mediolateral width of the proximal epiphysis | 5.21 |
|  | Mediolateral width of the distal epiphysis | 7.73 |
|  | Anteroposterior width of the proximal epiphysis | 6.25 |
|  | Anteroposterior width of proximal epiphysis | 5.67 |
