# Supplementary material for: The post-cranial anatomy and functional morphology of Conoryctes comma (Mammalia: Taeniodonta) from the Paleocene of North America
Source: PLoS One. 2024 Oct 25;19(10):e0311053. doi: 10.1371/journal.pone.0311053 (PMC11508153; doi:10.1371/journal.pone.0311053)
Supplement: S8 Table — Numbers are referring to the measurements as seen in S2 Fig. (DOCX) [file pone.0311053.s008.docx]

**S8 Table.**

| **Specimen** |  | **mm** |
| --- | --- | --- |
| **NMMNH P-48198** | Length of the lunate surface (1) | 22.10 |
|  | Width of the lunate surface (2) | 20.57 |
|  | Length from the tip of the ischiatic tuberosity to the iliopectineal eminence (3) | 44.41 |
|  | Total length of the fossae attaching the sacrum on the ventral view of the ilium (4) | 38.55 |
|  | Length of the fossa closer to the greater ischiatic notch (5) | 16.49 |
|  | Length of the fossa on the wing of the ilium (6) | 21.88 |
| **NMMNH P-47700** | Length of the lunate surface (1) | 20.29 |
|  | Length from the tip of the ischiatic tuberosity to the iliopectineal eminence (3) | 41.46 |
