# Supplementary material for: The post-cranial anatomy and functional morphology of Conoryctes comma (Mammalia: Taeniodonta) from the Paleocene of North America
Source: PLoS One. 2024 Oct 25;19(10):e0311053. doi: 10.1371/journal.pone.0311053 (PMC11508153; doi:10.1371/journal.pone.0311053)
Supplement: S9 Table — Numbers are referring to the measurements as seen in S3 Fig. (DOCX) [file pone.0311053.s009.docx]

**S9 Table.**

| **Specimen** |  | **mm** |
| --- | --- | --- |
| **NMMNH P-48052** | Femoral head mediolateral width (3) | 14.31 |
|  | Femoral head proximodistal length (4) | 16.31 |
| **NMMNH P-79457** | Total mediolateral width of the proximal epiphysis(1) | 31.34 |
|  | Femoral head anteroposterior length (2) | 15.89 |
|  | Femoral head mediolateral width (3) | 15.40 |
|  | Femoral head proximodistal length (4) | 11.96 |
