# Supplementary material for: The post-cranial anatomy and functional morphology of Conoryctes comma (Mammalia: Taeniodonta) from the Paleocene of North America
Source: PLoS One. 2024 Oct 25;19(10):e0311053. doi: 10.1371/journal.pone.0311053 (PMC11508153; doi:10.1371/journal.pone.0311053)
Supplement: S10 Table — (DOCX) [file pone.0311053.s010.docx]

**S10 Table.**

| **Specimen** |  | **mm** |
| --- | --- | --- |
| **NMMNH P-48052** | Anteroposterior length | 7.45 |
|  | Mediolateral width | 12.57 |
|  | Proximodistal length | 14.93 |
