# Supplementary material for: The post-cranial anatomy and functional morphology of Conoryctes comma (Mammalia: Taeniodonta) from the Paleocene of North America
Source: PLoS One. 2024 Oct 25;19(10):e0311053. doi: 10.1371/journal.pone.0311053 (PMC11508153; doi:10.1371/journal.pone.0311053)
Supplement: S11 Table — Numbers are referring to the measurements as seen in S4 Fig. (DOCX) [file pone.0311053.s011.docx]

**S11 Table.**

| **Specimen** |  | **mm** |
| --- | --- | --- |
| **NMMNH P-19494** | Distal tibia mediolateral width (3) | 19.62 |
|  | Distal tibia anteroposterior total length (6) | 16.33 |
| **NMMNH P-21509** | Proximal tibia mediolateral width (2) | 21.45 |
|  | Proximal fibular facet mediolateral width (4) | 5.62 |
|  | Proximal fibular facet anteroposterior length (5) | 7.57 |
|  | Proximal tibia anteroposterior total length (7) | 23.03* |
| **NMMNH P-48052** | Distal tibia mediolateral width (3) | 20.39 |
|  | Proximal fibular facet mediolateral width (4) | 5.61 |
|  | Proximal fibular facet anteroposterior length (5) | 7.07 |
|  | Distal tibia anteroposterior total length (6) | 14.79 |
|  | Proximal tibia anteroposterior total length (7) | 22.23* |
| **NMMNH P-48198** | Total proximodistal length (1) | 50.80 |
|  | Proximal tibia mediolateral width (2) | 27.68 |
|  | Distal tibia mediolateral width (3) | 21.52 |
|  | Proximal fibular facet mediolateral width (4) | 6.32 |
|  | Proximal fibular facet anteroposterior length (5) | 7.62 |
|  | Distal tibia anteroposterior total length (6) | 14.41 |
|  | Proximal tibia anteroposterior total length (7) | 17.87 |
| **NMMNH P-47700** | Proximal tibia mediolateral width (2) | 29.06* |
|  | Proximal fibular facet mediolateral width (4) | 3.97* |
|  | Proximal fibular facet anteroposterior length (5) | 6.30* |
|  | Proximal tibia anteroposterior total length (7) | 20.27* |
