# Supplementary material for: The post-cranial anatomy and functional morphology of Conoryctes comma (Mammalia: Taeniodonta) from the Paleocene of North America
Source: PLoS One. 2024 Oct 25;19(10):e0311053. doi: 10.1371/journal.pone.0311053 (PMC11508153; doi:10.1371/journal.pone.0311053)
Supplement: S12 Table — Numbers are referring to the measurements as seen in S5 Fig. (DOCX) [file pone.0311053.s012.docx]

**S12 Table.**

| **Specimen** |  | **mm** |
| --- | --- | --- |
| **NMMNH P-21509** | Mediolateral total width of the astragalar body (1) | 16.62 |
|  | Mediolateral total width of the astragalar head (2) | 12.33 |
|  | Anteroposterior length of the astragalar neck and head (3) | 11.71 |
|  | Mediolateral total width of the astragalar neck (4) | 9.13 |
|  | Mediolateral total width of the anteriormost edge of the astragalar body (5) | 13.62 |
|  | Mediolateral total width of the posteriormost edge of the astragalar body (6) | 13.44* |
|  | The anteroposterior total length of the astragalus (7) | 24.5 |
|  | Anteroposterior length of the ectal facet (8) | 11.53* |
|  | Mediolateral width of the ectal facet (9) | 7.5* |
|  | Anteroposterior length of the sustentacular facet (10) | 5.5* |
|  | Mediolateral width of the sustentacular facet (11) | 5.89* |
|  | Anteroposterior length of the medial tibial facet (12) | 12.06* |
|  | Dorsoplantar width of the medial tibial facet (13) | 8.08 |
|  | Dorsoplantar width of the astragalar head (14) | 6.72 |
|  | Anteroposterior length of the lateral tibial facet (15) | 11.28 |
|  | Dorsoplantar width of the lateral tibial facet (16) | 10.32 |
| **NMMNH P-48052** | Mediolateral total width of the astragalar body (1) | 18.68 |
|  | Mediolateral total width of the astragalar head (2) | 10.37 |
|  | Anteroposterior length of the astragalar neck and head (3) | 9.89 |
|  | Mediolateral total width of the astragalar neck (4) | 7.67* |
|  | Mediolateral total width of the anteriormost edge of the astragalar body (5) | 12.20 |
|  | Mediolateral total width of the posteriormost edge of the astragalar body (6) | 13.01 |
|  | Anteroposterior total length of the astragalus (7) | 22.63* |
|  | Anteroposterior length of the ectal facet (8) | 6.33* |
|  | Anteroposterior length of the sustentacular facet (10) | 7.94* |
|  | Mediolateral width of the sustentacular facet (11) | 6.44* |
|  | Anteroposterior length of the medial tibial facet (12) | 10.42* |
|  | Dorsoplantar width of the medial tibial facet (13) | 8.52* |
|  | Dorsoplantar width of the astragalar head (14) | 6.36 |
|  | Anteroposterior length of the lateral tibial facet (15) | 9.95* |
|  | Dorsoplantar width of the lateral tibial facet (16) | 8.11* |
| **NMMNH P-48198** | Mediolateral total width of the astragalar body (1) | 19.45 |
|  | Mediolateral total width of the astragalar head (2) | 12.53 |
|  | Anteroposterior length of the astragalar neck and head (3) | 11.89 |
|  | Mediolateral total width of the astragalar neck (4) | 8.73* |
|  | Mediolateral total width of the anteriormost edge of the astragalar body (5) | 15.15 |
|  | Mediolateral total width of the posterior most edge of the astragalar body (6) | 13.78 |
|  | Anteroposterior total length of the astragalus (7) | 23.97 |
|  | Anteroposterior length of the ectal facet (8) | 11.6 |
|  | Mediolateral width of the ectal facet (9) | 7.96 |
|  | Anteroposterior length of the sustentacular facet (10) | 8.12 |
|  | Mediolateral width of the sustentacular facet (11) | 6.44 |
|  | Anteroposterior length of the medial tibial facet (12) | 11.12 |
|  | Dorsoplantar width of the medial tibial facet (13) | 10.35 |
|  | Dorsoplantar width of the astragalar head (14) | 7.72 |
|  | Anteroposterior length of the lateral tibial facet (15) | 12.13 |
|  | Dorsoplantar width of the lateral tibial facet (16) | 11.73 |
