# Supplementary material for: The post-cranial anatomy and functional morphology of Conoryctes comma (Mammalia: Taeniodonta) from the Paleocene of North America
Source: PLoS One. 2024 Oct 25;19(10):e0311053. doi: 10.1371/journal.pone.0311053 (PMC11508153; doi:10.1371/journal.pone.0311053)
Supplement: S13 Table — Numbers are referring to the measurements as seen in S6 Fig. (DOCX) [file pone.0311053.s013.docx]

**S13 Table.**

| **Specimen** |  | **mm** |
| --- | --- | --- |
| **NMMNH P-47866** | Total anteroposterior length (1) | 27.78 |
|  | Total mediolateral width between the sustentacular facet and the peroneal process (2) | 18.73 |
|  | Anteroposterior length of the ectal facet (3) | 9.85 |
|  | Mediolateral width of the ectal facet (4) | 6.96 |
|  | Anteroposterio length of the sustentacular facet (5) | 6.77 |
|  | Mediolateral width of the sustentacular facet (6) | 6.56 |
|  | Mediolateral width of the cuboid facet (7) | 7.72 |
|  | Dorsoplantar length of the cuboid facet (8) | 6.77 |
|  | Anteroposterior length of the peroneal process (9) | 5.69 |
|  | Distance between the tuber calcanei and the most posterior edge of the ectal facet (10) | 12.10 |
|  | Distance between the most anterior edge of the ectal facet and the most anterior part of the calceneum (11) | 7.21 |
|  | Distance between the most posterior edge of the ectal facet and the most anterior part of the calceneum (12) | 15.65 |
|  | Mediolateral width of the tuber calcaneum at the middle point (13) | 6.86 |
|  | Dorsoplantar length of the anterior edge of the calcaneum (14) | 11.68 |
|  | Dorsoplantar length at the middle point of the calcaneum (15) | 11.55 |
|  | Dorsoplantar length of the tubercle calcanei (16) | 10.13 |
|  | Mediolateral width of the anterior plantar tubercle (17) | 4.51 |
| **NMMNH P-48052** | Total anteroposterior length (1) | 33.98 |
|  | Anteroposterior length of the ectal facet (3) | 11.09 |
|  | Mediolateral width of the ectal facet (4) | 9.68 |
|  | Mediolateral width of the cuboid facet (7) | 8.66 |
|  | Dorsoplantar length of the cuboid facet (8) | 7.76 |
|  | Anteroposterior length of the peroneal process (9) | 7.04 |
|  | Distance between the tuber calcanei and the most posterior edge of the ectal facet (10) | 17.22 |
|  | Distance between the most anterior edge of the ectal facet and the most anterior part of the calcaneum (11) | 8.10 |
|  | Distance between the most posterior edge of the ectal facet and the most anterior part of the calcaneum (12) | 17.14 |
|  | Mediolateral width of the tuber calcaneum at the middle point (13) | 6.81 |
|  | Dorsoplantar length of the anterior edge of the calcaneum (14) | 11.67 |
|  | Dorsoplantar length at the middle point of the calcaneum (15) | 14.28 |
|  | Dorsoplantar length of the tubercle calcanei (16) | 10.57 |
|  | Mediolateral width of the anterior plantar tubercle (17) | 4.77 |
| **NMMNH P-48198 (right)** | Total anteroposterior length (1) | 34.12 |
|  | Anteroposterior length of the ectal facet (3) | 10.89 |
|  | Mediolateral width of the ectal facet (4) | 6.16 |
|  | Anteroposterio length of the sustentacular facet (5) | 6.90 |
|  | Mediolateral width of the sustentacular facet (6) | 7.31 |
|  | Mediolateral width of the cuboid facet (7) | 8.46 |
|  | Dorsoplantar length of the cuboid facet (8) | 6.46 |
|  | Distance between the tuber calcanei and the most posterior edge of the ectal facet (10) | 18.11 |
|  | Distance between the most anterior edge of the ectal facet and the most anterior part of the calcaneum (11) | 8.63 |
|  | Distance between the most posterior edge of the ectal facet and the most anterior part of the calcaneum (12) | 16.48 |
|  | Mediolateral width of the tuber calcaneum at the middle point (13) | 7.72 |
|  | Dorsoplantar length of the anterior edge of the calcaneum (14) | 13.08 |
|  | Dorsoplantar length at the middle point of the calcaneum (15) | 14.63 |
|  | Dorsoplantar length of the tubercle calcanei (16) | 10.65 |
| **NMMNH P-48198 (left)** | Total anteroposterior length (1) | 33.78 |
|  | Anteroposterior length of the ectal facet (3) | 9.95 |
|  | Mediolateral width of the ectal facet (4) | 5.54 |
|  | Mediolateral width of the cuboid facet (7) | 7.48* |
|  | Dorsoplantar length of the cuboid facet (8) | 6.48 |
|  | Distance between the tuber calcanei and the most posterior edge of the ectal facet (10) | 18.08 |
|  | Distance between the most anterior edge of the ectal facet and the most anterior part of the calcaneum (11) | 8.26 |
|  | Distance between the most posterior edge of the ectal facet and the most anterior part of the calcaneum (12) | 16.02 |
|  | Mediolateral width of the tuber calcaneum at the middle point (13) | 8.12 |
|  | Dorsoplantar length of the anterior edge of the calcaneum (14) | 13.18 |
|  | Dorsoplantar length at the middle point of the calcaneum (15) | 14.10 |
|  | Dorsoplantar length of the tubercle calcanei (16) | 11.52 |
