# Supplementary material for: The post-cranial anatomy and functional morphology of Conoryctes comma (Mammalia: Taeniodonta) from the Paleocene of North America
Source: PLoS One. 2024 Oct 25;19(10):e0311053. doi: 10.1371/journal.pone.0311053 (PMC11508153; doi:10.1371/journal.pone.0311053)
Supplement: S14 Table — (DOCX) [file pone.0311053.s014.docx]

**S14 Table.**

| **Specimen** |  | **mm** |
| --- | --- | --- |
| **NMMNH P-47700** |  |  |
| **IV metatarsals** | total length | 28.35 |
|  | perimeter at midshaft | 21 |
|  | mediolateral width of the proximal epiphysis | 5.68 |
|  | mediolateral width of the distal epiphysis | 8.39 |
|  | anteroposterior width of the proximal epiphysis | 8.23 |
|  | anteroposterior width of the proximal epiphysis | 5.83 |
| **V metatarsals** | total length | 14.27 |
|  | perimeter at midshaft | 1.5 |
|  | mediolateral width of the proximal epiphysis | 5.98 |
|  | mediolateral width of the distal epiphysis | 5.72 |
|  | anteroposterior width of the proximal epiphysis | 5.45 |
|  | anteroposterior width of the proximal epiphysis | 3.95 |
