# Supplementary figures and images for: The post-cranial anatomy and functional morphology of Conoryctes comma (Mammalia: Taeniodonta) from the Paleocene of North America
Source: PLoS One. 2024 Oct 25;19(10):e0311053. doi: 10.1371/journal.pone.0311053 (PMC11508153; doi:10.1371/journal.pone.0311053)

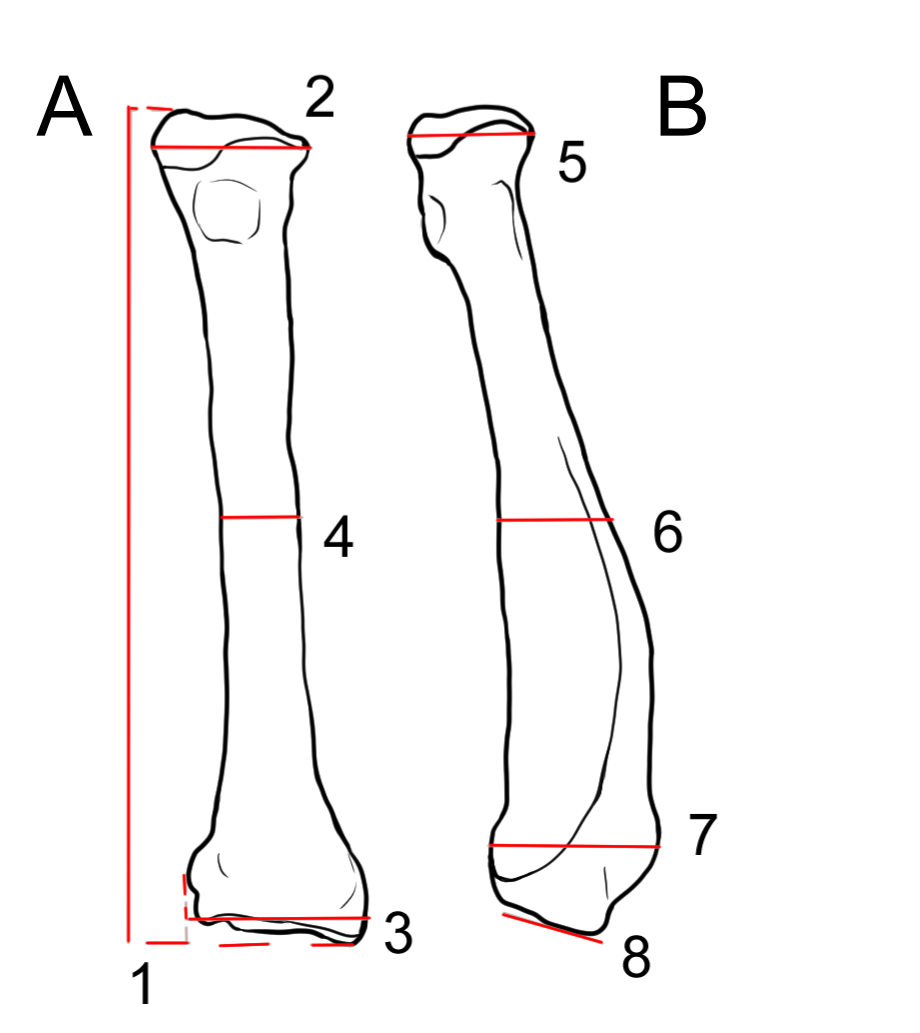

Supplement: S1 Fig — Drawing of the measurements taken of the radius in posterior (A) and lateral (B) views. Total proximodistal length (1), mediolateral width of the proximal epiphysis (2), mediolateral width of the distal epiphysis (3), mediolateral width at the middle of the shaft (4), anteroposterior width of the proximal epiphysis (5), anteroposterior width at the middle of the shaft (6), anteroposterior width of the distal epiphysis (7), anteroposterior width of the distal articular fovea (8). (TIFF) [file pone.0311053.s015.tiff]

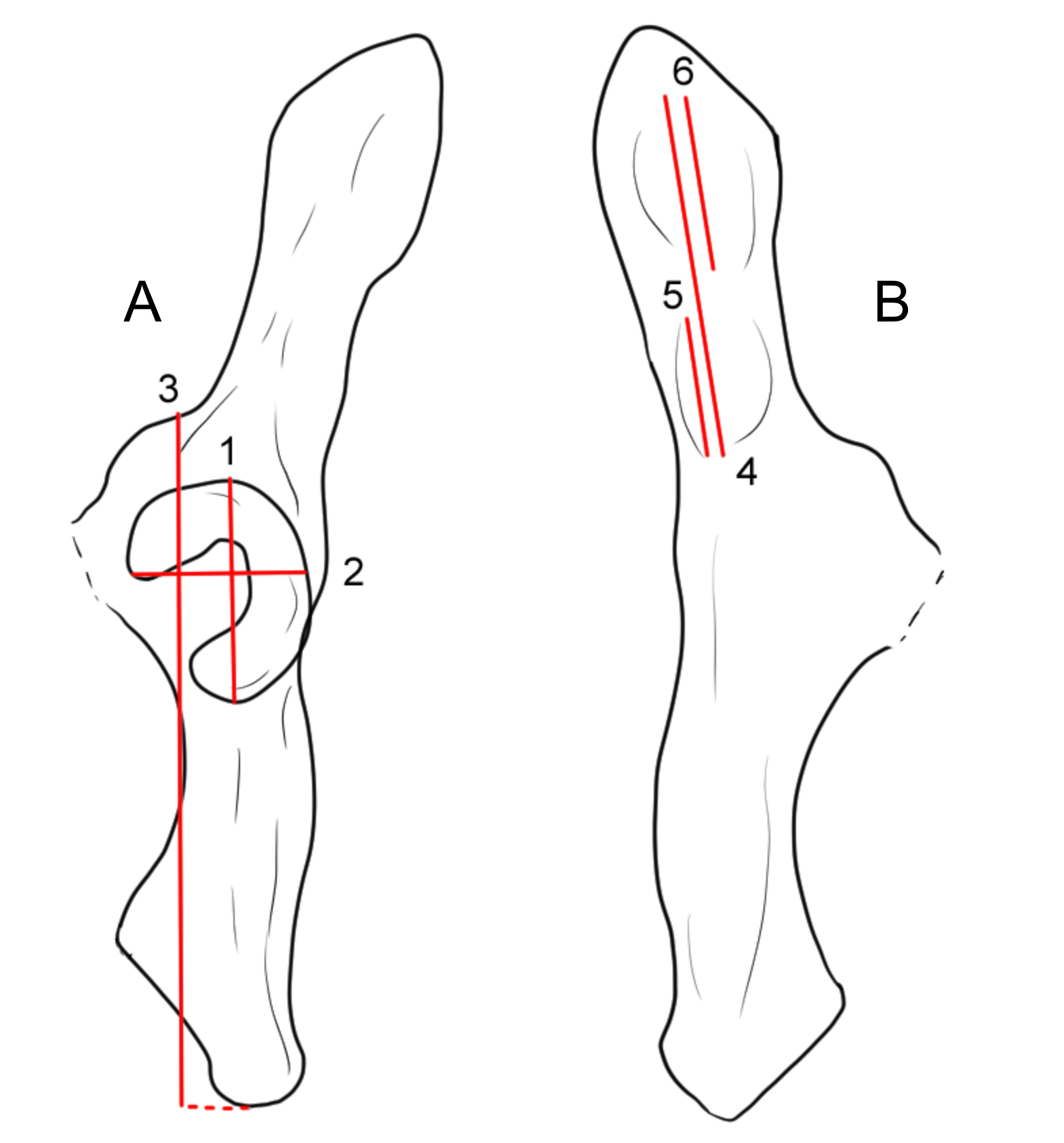

Supplement: S2 Fig — Drawing of the measurements taken of the pelvis in lateral (A) and medial (B) views. Length of the lunate surface (1), width of the lunate surface (2), length from the tip of the ischiatic tuberosity to the iliopectineal eminence (3), total length of the fossae attaching the sacrum on the ventral view of the ilium (4), length of the fossa closer to the greater ischiatic notch (5), length of the fossa on the wing of the ilium (6). (TIFF) [file pone.0311053.s016.tiff]

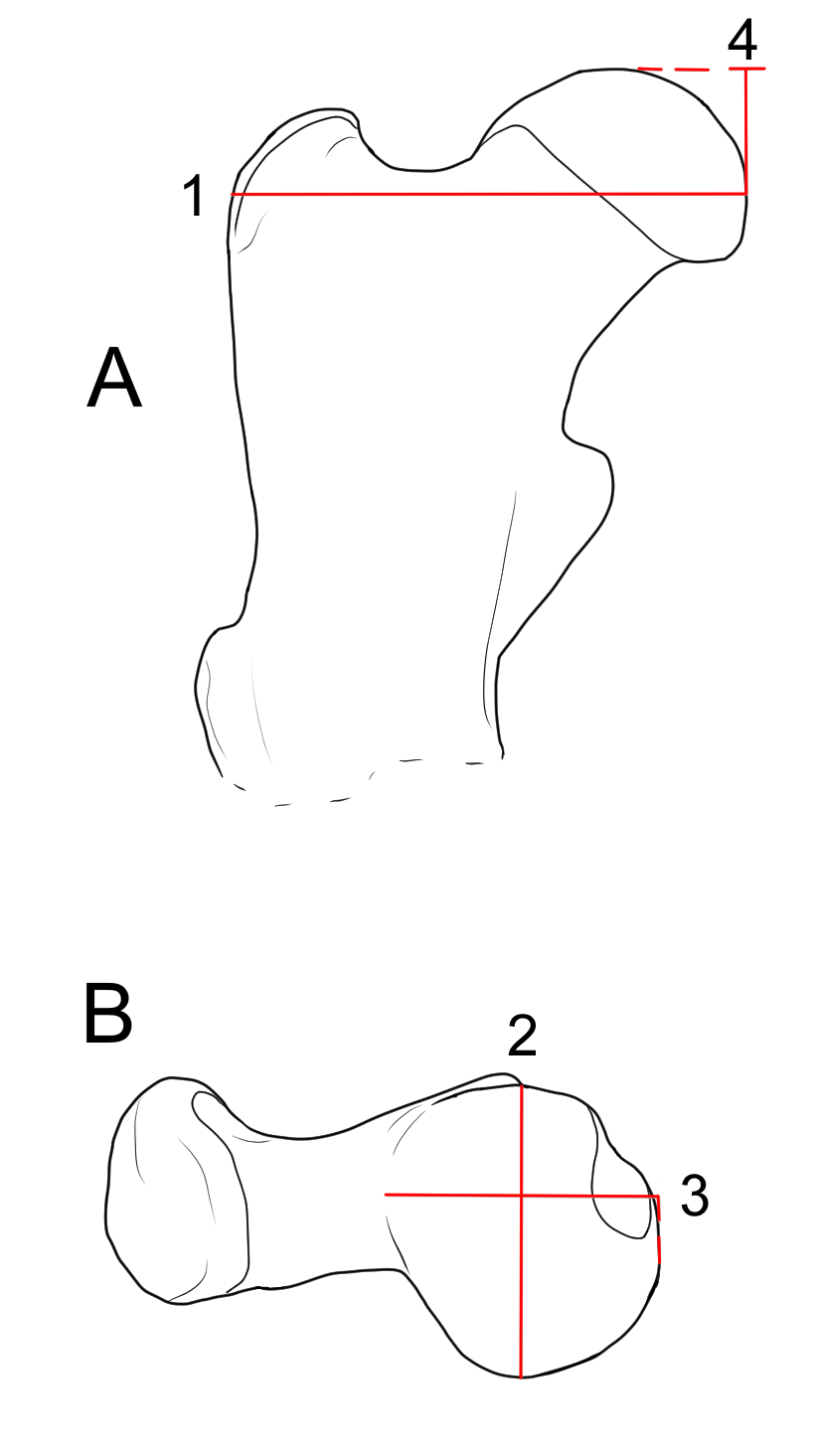

Supplement: S3 Fig — Drawing of the measurements taken of the femur in anterior (A) and proximal (B) views. Total mediolateral width of the proximal epiphysis (1), femoral head anteroposterior length (2), femoral head mediolateral width (3), femoral head proximodistal length (4). (TIFF) [file pone.0311053.s017.tiff]

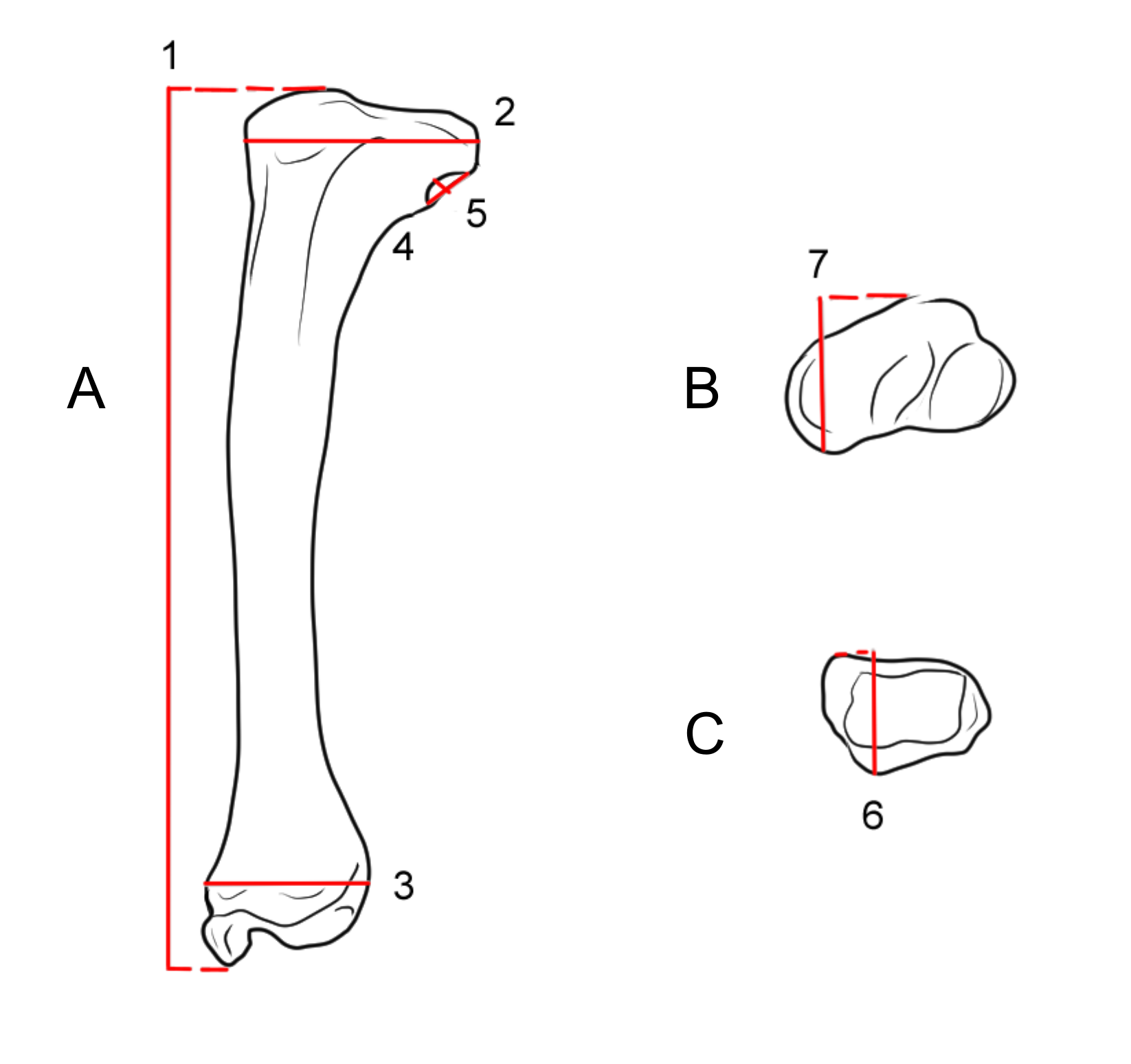

Supplement: S4 Fig — Drawing of the measurements taken of the tibia in anterior (A), proximal (B) and distal (C) views. Total proximodistal length (1), proximal tibia mediolateral width (2), distal tibia mediolateral width (3), proximal fibular facet mediolateral width (4), proximal fibular facet anteroposterior length (5), distal tibia anteroposterior total length (6), proximal tibia anteroposterior total length (7). (TIFF) [file pone.0311053.s018.tiff]

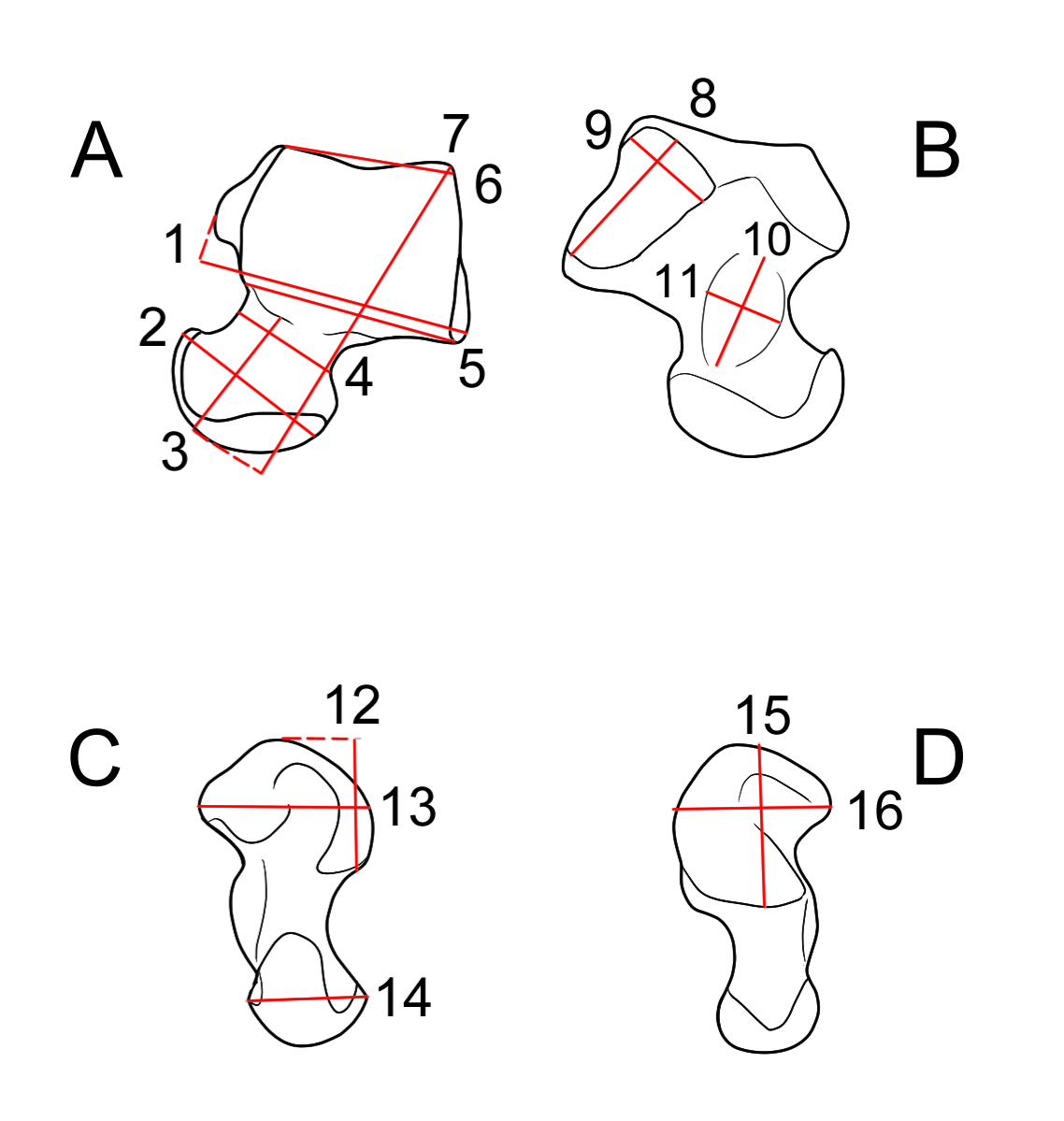

Supplement: S5 Fig — Drawing of measurements of the astragalus in dorsal (A), plantar (B), medial (C) and lateral (D) views. Mediolateral total width of the astragalar body (1), mediolateral total width of the astragalar head (2), anteroposterior length of the astragalar neck and head (3), mediolateral total width of the astragalar neck (4), mediolateral total width of the anterior most edge of the astragalar body (5), mediolateral total width of the posterior most edge of the astragalar body (6), anteroposterior total length of the astragalus (7), anteroposterior length of the ectal facet (8), mediolateral width of the ectal facet (9), anteroposterior length of the sustentacular facet (10), mediolateral width of the sustentacular facet (11), anteroposterior length of the medial tibial facet (12), dorsoplantar width of the medial tibial facet (13), dorsoplantar width of the astragalar head (14), anteroposterior length of the lateral tibial facet (15), dorsoplantar width of the lateral tibial facet (16). (TIFF) [file pone.0311053.s019.tiff]

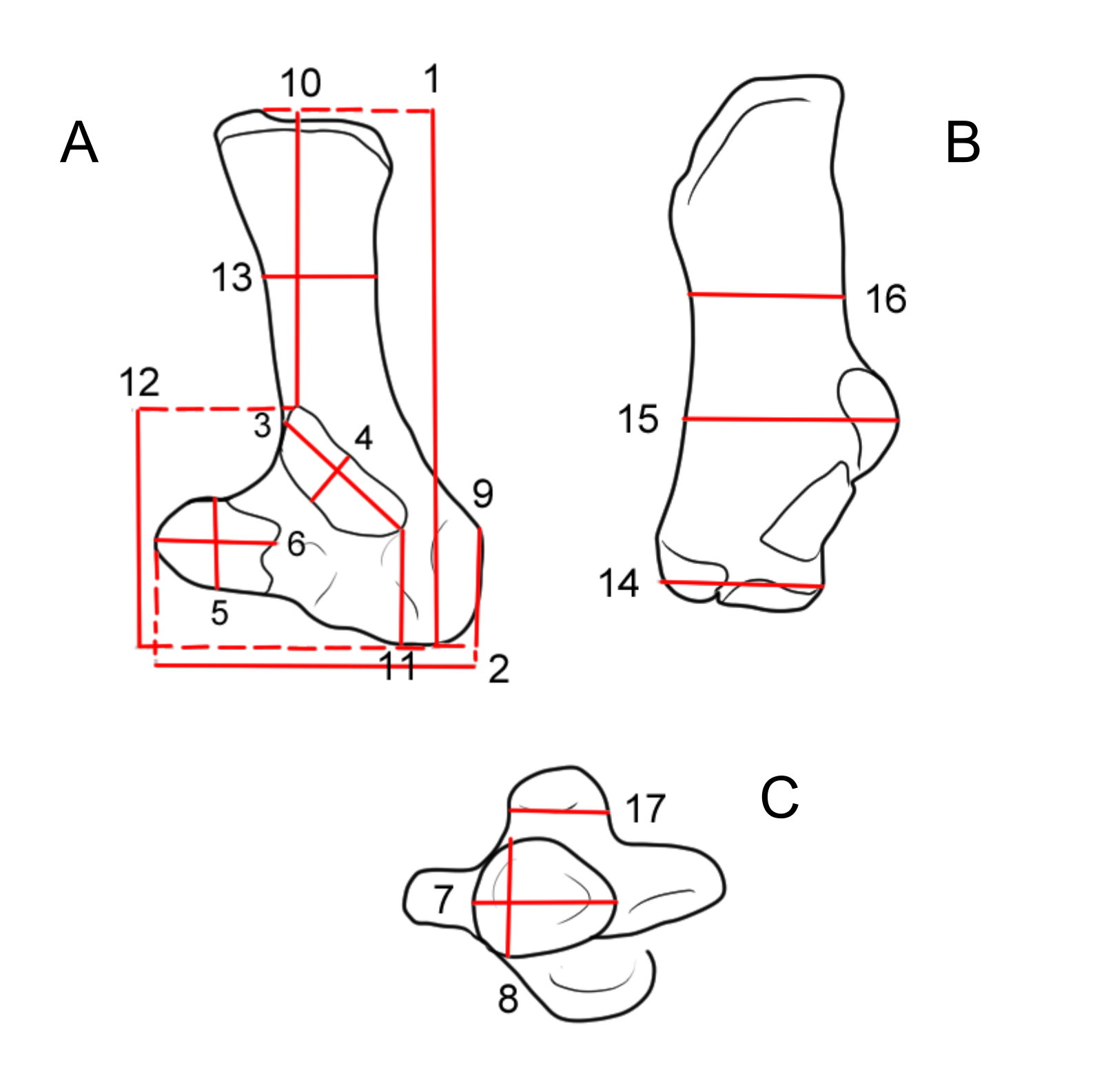

Supplement: S6 Fig — Drawing of the measurements taken on the calcaneum in dorsal (A), medial (B) and anterior (C) views. Total anteroposterior length (1), total mediolateral width between the sustentacular facet and the peroneal process (2), anteroposterior length of the ectal facet (3), mediolateral width of the ectal facet (4), anteroposterior length of the sustentacular facet (5), mediolateral width of the sustentacular facet (6), mediolateral width of the cuboid facet (7), dorsoplantar length of the cuboid facet (8), anteroposterior length of the peroneal process (9), distance the tuber calcanei to the most posterior edge of the ectal facet (10), distance between the most anterior edge of the ectal facet and the most anterior part of the calceneum (11), distance between the most posterior edge of the ectal facet and the most anterior part of the calceneum (12), mediolateral width of the tuber calcaneum at the middle point (13), dorsoplantar length of the anterior edge of the calcaneum (14), dorsoplantar length at the middle point of the calcaneum (15), dorsoplantar length of the tubercle calcanei (16), mediolateral width of the anterior plantar tubercle (17). (TIFF) [file pone.0311053.s020.tiff]
